# USP1 driven mitotic dysregulation and PLK1 stabilization confer Lenvatinib resistance in hepatocellular carcinoma

**DOI:** 10.1186/s13046-026-03683-w

**Published:** 2026-03-13

**Authors:** Yuwei Qiang, Saiyan Bian, Yun Tong, Weiting Chen, Zhangzhi Tang, Chengchen Dai, Mingyu Liu, Banglong Xu, Lihan Jiang, Kexin Ma, Xuyang He, Jinlong Li, Mingbing Xiao, Wenjie Zheng

**Affiliations:** 1 Department of Oncology, Research Center of Clinical Medicine, Affiliated Hospital of Nantong University, Medical School of Nantong University, Nantong, 226001 China; 2 Department of Gastroenterology, Affiliated Hospital of Nantong University, Medical School of Nantong University, Nantong, 226001 China; 3 College of Pharmacology, Nantong University, Nantong, China; 4 Department of Pathology, Medical School of Nantong University, Nantong, 226001 China

**Keywords:** HCC, Lenvatinib, Chromosome misalignment, USP1, PLK1, STUB1

## Abstract

**Background:**

Lenvatinib is a first-line therapeutic option for advanced hepatocellular carcinoma (HCC), while acquired resistance severely limits its clinical efficacy. The precise molecular targets reversing Lenvatinib resistance remain inadequately explored. This study aims to elucidate the role of ubiquitin-specific peptidase 1 (USP1) in mediating resistance and identify potential therapeutic targets to improve treatment outcomes in HCC.

**Methods:**

Comprehensive analyses employing genetic modulation (USP1 knockdown and overexpression), pharmacological inhibition, and a series of in vitro and in vivo assays were conducted to assess the effects of USP1 on HCC cell sensitivity to Lenvatinib. Mass spectrometry-based proteomics, integrated bioinformatics analysis, and subsequent molecular validation techniques were utilized to systematically identify key USP1 substrates and interacting E3 ubiquitin ligases. Additionally, virtual screening was conducted using the ChemDiv library to identify potential inhibitors, followed by validation of candidate compounds through in vitro and in vivo experiments.

**Results:**

Depletion of USP1 markedly enhanced sensitivity to Lenvatinib in HCC cells, while its overexpression induced resistance. Notably, USP1 knockdown led to obvious chromosome misalignment during metaphase in the presence of Lenvatinib. Mechanistically, Polo-like kinase 1 (PLK1) was identified as a critical substrate stabilized by USP1-mediated deubiquitination, essential for maintaining chromosome alignment and facilitating drug resistance. Additionally, we discovered that the E3 ubiquitin ligase STIP1 homology and U-box-containing protein 1 (STUB1) antagonized with USP1 to regulate PLK1 stability, further modulating resistance of HCC cells. c-Myc was identified as a transcriptional regulator of USP1, establishing a positive feedback loop as USP1/ PLK1/ c-Myc axis. Importantly, NTUZLB-001, a novel compound identified via in silico screening, effectively overcame resistance by promoting PLK1 destabilization.

**Conclusions:**

Our findings reveal a novel mechanism wherein USP1 promotes Lenvatinib resistance in HCC by regulating chromosome alignment through PLK1 deubiquitination. Targeting the USP1/PLK1 axis with NTUZLB-001 represents a promising therapeutic strategy to overcome drug resistance and enhance the clinical efficacy of Lenvatinib in HCC treatment.

**Supplementary Information:**

The online version contains supplementary material available at 10.1186/s13046-026-03683-w.

## Introduction

Hepatocellular carcinoma (HCC) ranks among the most prevalent malignancies and represents the fourth leading cause of cancer-related mortality globally. Its rising incidence poses a persistent challenge to public health worldwide [1]. Currently, conventional liver cancer treatments encompass surgical resection, hepatic artery embolization, radiotherapy, anhydrous alcohol injection, and biotherapy. In 2018, Lenvatinib, a multitarget inhibitor of VEGF receptors, FGF receptors, PDGF receptor-α, RET, and KIT, was approved by the Food and Drug Administration as a first-line treatment for advanced HCC. Compared to sorafenib, Lenvatinib demonstrated significantly improved overall survival and progression-free survival rates [2]. However, the clinical utility of Lenvatinib is often hindered by the emergence of acquired resistance [3]. Thus, elucidating the mechanisms of Lenvatinib resistance and identifying potential targeted strategies are of great importance.

Numerous studies have highlighted the critical role of deubiquitination modifications in tumor progression [4]. Seven families of deubiquitinating enzymes have been identified, including ubiquitin-specific proteases (USPs), ubiquitin C-terminal hydrolases (UCHs), ovarian tumor-related proteases (OTUs), Machado-Josephin domain-containing proteases (MJDs), JAB1/MPN/Mov34 metalloenzymes (JAMMs), newly characterized zinc finger proteases (ZUPs/ZUFSPs), and the MIU-containing novel DUB family (MINDs) [5]. Among them, the USP family represents the most abundant subfamily of deubiquitinating enzymes [6]. Emerging evidence has linked the USP family to drug resistance in cancer cells. For instance, USP12 has been shown to induce bortezomib resistance in multiple myeloma by activating autophagy through deubiquitination of the autophagy mediator HMGB1 [7]. The CDK-USP29 axis has been implicated in promoting progression and drug resistance in triple-negative breast cancer by deubiquitinating TWIST1 [8]. Moreover, USP11 promoted ovarian cancer Carboplatinum resistance by stabilizing BIP [9]. Recent studies have implicated USP1 in the malignant behaviors of HCC cells, including proliferation and metastasis [10–12]. However, its effects on Lenvatinib sensitivity and the underlying mechanisms remain largely unexplored.

Polo-like kinase 1 (PLK1) is a serine/threonine protein kinase ubiquitously expressed in eukaryotic cells and plays a critical role in the initiation, maintenance, and termination of mitosis [13–15]. A growing body of evidence has demonstrated that overexpression of PLK1 is essential for tumor growth and is associated with poor prognosis in various cancers [16, 17]. Several PLK1-targeting inhibitors have been evaluated in clinical trials [18]. The KD (kinase domain) inhibitor, volasertib, has demonstrated antitumor activity in patients with ovarian cancer. The ATP-competitive inhibitor Onvansertib, in combination with decitabine, significantly inhibited the progression of acute myeloid leukemia (AML) [19]. Moreover, recent studies have indicated that PLK1 contributes to drug resistance in tumor cells. PLK1 suppression hindered breast cancer metastasis with acquired palbociclib resistance, owing to a PLK1-mediated poor response to cyclin-dependent kinase 4/6 inhibitors (CDK4/6i) [20]. Inhibition of PLK1 overcame oxaliplatin resistance in colorectal cancer by modulating the PLK1-MYC-CDC7 signaling axis [21]. Consequently, PLK1 is considered a promising therapeutic target for overcoming drug resistance.

In this study, we conducted in-depth investigations at multiple levels, identifying USP1 as a critical regulator of Lenvatinib resistance in HCC. We further demonstrated that USP1 regulates chromosome alignment by interacting with and stabilizing PLK1, thus contributing to Lenvatinib resistance. The USP1/PLK1/c-Myc axis may form a positive feedback loop. Furthermore, our data suggest that combining Lenvatinib with the USP1 inhibitor ML323 or the newly identified agent NTUZLB-001 could synergistically induce HCC cell death both in vitro and in vivo.

## Methods

### Cell culture, synchronization and establishment of Lenvatinib-resistant cell line

HCC cell lines HCCLM3(Cat. ZQ0023), MHCC97-H(Cat. ZQ0020), MHCC97-L(Cat. ZQ0019), SMMC7721(Cat. ZQ0029), SK-Hep1(Cat. ZQ0030), Huh7 (Cat. ZQ0025), Hep3B(Cat. ZQ0024), SNU449 (Cat. ZQ0787) and human embryonic kidney cell line HEK293T (Cat. ZQ0033) were obtained from Zhong Qiao Xin Zhou Biotechnology (Shanghai, China). All cell lines were cultured in high-glucose medium supplemented with 10% fetal bovine serum (FBS) at 37 °C in a humidified atmosphere with 5% CO_2_. Cells were synchronized at the G1/S phase by treatment with 2 mM thymidine (MedChemExpress) for 24 h, followed by release into fresh medium for 10 h. Subsequently, cells were treated with 10 µM MG132 for 2.5 h to synchronize at metaphase. To establish the lenvatinib-resistant Huh7 cell line (Huh7-R), cells were gradually exposed to increasing concentrations of Lenvatinib (Selleck), starting at 3 µmol/L. The culture medium was replaced every 48 h, and cells were passaged once they covered approximately 90% of the culture dish. After each passage, the Lenvatinib concentration was increased by 0.5 µmol/L, continuing until the cells proliferated rapidly in the presence of 30 µmol/L Lenvatinib. This process took a minimum of 6 months to complete. Cell stocks were generated within five passages, and all experiments were conducted within eight passages to ensure consistency and stability.

### Clinical samples

HCC samples were obtained from patients who underwent liver biopsy and subsequent Lenvatinib treatment at the Affiliated Hospital of Nantong University (Nantong, Jiangsu, China). The study complies with the Declaration of Helsinki and was approved by the Ethics Committee of the Affiliated Hospital of Nantong University (approval No. 2023-L038; date: 03/02/2023), which operates in accordance with the Declaration of Helsinki. Given the retrospective design and use of de-identified data and specimens, the Committee granted a waiver of informed consent.

### Cell proliferation assays

HCC cells were seeded at a density of 3000 cells per 100 µL in 96-well plates. After 24 h, the medium was replaced with fresh medium containing 10 µM Lenvatinib. After 48 h, cells were treated with Cell Counting Kit-8 (CCK-8) reagent (Beyotime Biotechnology, Shanghai, China), diluted 1:10 with serum-free medium, and incubated for 2 h. The absorbance was measured at 450 nm. For colony formation assays, 500 HCC cells from each group were seeded in 6-well plates. After 7 days, the agents including DMSO, Lenvatinib, ML323, and/or Onvansertib were added to the culture media for an additional 7 days. The plates were then fixed with 4% paraformaldehyde (PFA) for at least 40 min and stained with 0.1% crystal violet.

### Transwell assays

Migration assays were performed using 8-µm pore polycarbonate membrane transwell plates (Corning, Acton, MA, USA). 3 × 10^5^ HCC cells from each group were seeded in the upper chamber without serum. The lower chamber of the 24-well plates was filled with 500 µL medium containing DMSO or Lenvatinib. After 48 h, cells were fixed with 4% PFA for at least 40 min and stained with 0.1% crystal violet. The cells were then observed under a microscope.

### Cells transfection and stable transfected cell lines

Plasmids coding for USP1 (p3×FLAG-CMV-10-USP1), USP1-C90S(p3×FLAG-CMV-10-USP1-C90S), PLK1(p3×FLAG-CMV-10-PLK1), STUB1(p3×FLAG-CMV-10-STUB1), and ShUSP1-expressing plasmids were obtained from TransheepBio (Shanghai, China). Transfection was performed using the Lipofectamine 3000 Transfection Kit (Thermo Fisher Scientific, USA) according to the manufacturer’s instructions. Puromycin was used to select stably transfected cells. The sequences of the plasmids used were as follows: ShUSP1-1: CCGGGCTAGTGGTTTGGAGTTTGATCTCGAGATCAAACTCCAAACCACTAGCTTTTTT. ShUSP1-2:CCGGCCAGTGACCAAACAGGCATTACTCGAGTAATGCCTGTTTGGTCACTGGTTTTTT. ShNC, Control, WT, C90S, HA-Ub, HA-K48-Ub, HA-K63-Ub, USP1-UTM, and PLK1-UTM plasmids were obtained from TransheepBio (Shanghai, China). The p3XFLAG-CMV-10-USP1(C90S) mutant plasmid were generated using a homologous recombination–based cloning strategy. Briefly, the parental p3XFLAG-CMV-10-USP1 (TSB112131-5) construct was used as the template and linearized by double digestion with HindIII and EcoRI, followed by gel purification to obtain the p3XFLAG-CMV-10 backbone. The USP1 C90S point mutation was introduced by overlap-extension PCR using the same parental plasmid as template. Two overlapping fragments containing the desired substitution were first amplified with mutation-specific primers, in which the catalytic cysteine codon TGT was changed to the serine codon AGT. The full-length mutant USP1 insert flanked by homology arms matching the HindIII/EcoRI-linearized vector was then generated by a second-round PCR using the outer primers. The resulting PCR product was assembled into the linearized p3XFLAG-CMV-10 vector via homologous recombination to obtain the final p3XFLAG-CMV-10-USP1(C90S) expression plasmid, and the mutation was confirmed by Sanger sequencing prior to downstream experiments.

### Western blot assay

Western blot assays were performed following standard procedures to measure the expression levels of proteins in cells. Total protein extracts were prepared and subjected to SDS-PAGE for separation, and the proteins were then transferred onto polyvinylidene difluoride (PVDF) membranes (Bio-Rad, Hercules, CA, USA). The membranes were blocked with 5% non-fat milk in TBST (Tris-buffered saline with 0.1% Tween-20) for 2 h at room temperature to prevent non-specific binding. Subsequently, the membranes were incubated with primary antibodies, which were diluted in 5% non-fat milk/TBST, as follows: anti-USP1 (Proteintech, Cat#1434-1-AP, 1:1000 v/v), anti-PLK1 (Cell Signaling, Cat#208G4, 1:500 v/v), anti-β-actin (Proteintech, Cat#81115-1-RR, 1:1000 v/v), anti-HA (Abclonal, Cat#AE008, 1:500 v/v), anti-DDDDK-Tag (Abclonal, Cat#AE005, 1:500 v/v), anti-Cyclin B1 (Santa Cruz, Cat#sc245, 1:500 v/v), anti-CDK1 (Santa Cruz, Cat#sc53219, 1:500 v/v), anti-STUB1 (Santa Cruz, Cat#sc133066, 1:500 v/v), anti-c-Myc (Abclonal, Cat#A1309, 1:1000 v/v), and anti-phospho-c-Myc S62 (Abclonal, Cat#AP0082, 1:1000 v/v). The membranes were incubated with primary antibodies at 4 °C overnight, followed by three washes with TBST. For detection of the target proteins, the membranes were incubated with appropriate secondary antibodies conjugated with horseradish peroxidase (HRP) at room temperature for 1 h. After washing three times with TBST, the protein bands were visualized using enhanced chemiluminescence (ECL) reagents (Thermo Fisher Scientific, USA) according to the manufacturer’s instructions. The images were captured using a chemiluminescence imaging system (Bio-Rad, Hercules, CA, USA). The expression levels of the proteins were quantified using ImageJ software (NIH, Bethesda, MD, USA), with β-actin serving as a loading control.

### 3D spheroid model construction

Two thousand HCC cells from different groups were mixed with collagen I (Meilun Biotechnology, 5 µg/mL). The cell-collagen mixture was then plated into a 96-well Corning Round Bottom Ultra-Low Attachment Microplate (Corning, USA). Spheroid formation was monitored and photographed on the 1st, 3rd, and 5th days. On day 5, some groups were treated with Lenvatinib or ML323. The spheroid volume was measured to assess growth and changes over time.

### RNA extraction and real-time quantitative PCR

Total RNA was extracted using TRIzol reagent (Invitrogen, USA) according to the manufacturer’s instructions. cDNA synthesis was performed using the HiScript III RT SuperMix (Vazyme, Nanjing, China). Real-time quantitative PCR was conducted with SYBR Green Master Mix (Applied Biosystems Inc., MA, USA). β-actin was used as the reference control. The expression levels of target genes were calculated using the 2^−ΔΔCT^ method. The sequences of the primers used are listed in Supplementary Table S1.

### Chromatin immunoprecipitation (ChIP)

Cells were first cross-linked with 1% formaldehyde solution for 10 min at room temperature, and the cross-linking reaction was quenched by adding 1.25 M glycine. Afterward, cells were harvested and lysed using an appropriate lysis buffer, followed by sonication to fragment the chromatin. The resulting lysates were incubated overnight at 4 °C with a target-specific antibody against c-MYC (Abclonal, A1309) or a control IgG antibody. To reduce non-specific binding, the immunoprecipitated complexes were pre-cleared by incubation with ChIP-grade protein A/G magnetic beads for 2 h. The antibody-bound chromatin was isolated, and the DNA was eluted from the complexes. Cross-links were reversed by incubating at 65 °C, and the DNA was purified using a standard ChIP DNA purification kit. The high-quality ChIP DNA was subsequently used for quantitative RT-PCR analysis as previously described.

### Dual-luciferase promoter reporter assay

USP1 promoter activity was assessed using a dual-luciferase reporter system (TRANSHEEP BIO, China). Briefly, the indicated USP1 promoter fragment was cloned into the pGL4 firefly luciferase reporter vector, and the corresponding empty pGL4 vector was used as the negative control. HCC cells were seeded in 24-well plates and co-transfected with the USP1 promoter–luciferase reporter and the Renilla luciferase internal control plasmid (pRL-TK; TRANSHEEP BIO) using the indicated transfection reagent. Cells were co-transfected with c-Myc expression plasmid or treated with 10,058-F4 after transfection. At 48 h post-transfection, luciferase activities were measured sequentially using a dual-luciferase assay kit (TRANSHEEP BIO) on a luminometer according to the manufacturer’s instructions. Firefly luciferase signals were normalized to Renilla luciferase to control for transfection efficiency, and data were presented as relative luciferase activity compared with the corresponding control group.

### Co-immunoprecipitation(Co-IP) assay and ubiquitination assay

Cells were lysed on ice for 30 min using Radio Immunoprecipitation Assay (RIPA) Lysis Buffer (Beyotime Biotechnology, Shanghai, China) containing protease inhibitors. The cell lysates were pre-cleared by incubation with rabbit IgG for 3 h to remove non-specific binding. Afterward, the lysates were incubated with the specified primary antibody for 12 h at 4 °C. The antigen-antibody complexes were then captured by incubation with protein A/G agarose beads (Bioworld, USA) for 2.5 h. Following incubation, the protein-antibody-bead complexes were washed three times with ice-cold lysis buffer to remove unbound proteins. The immunoprecipitated proteins were eluted and subsequently analyzed by western blotting.

### Immunofluorescence (IF) staining

HCC cells and Huh7-R cells were seeded on the glass slides in 24-well plates. After 24 h, the cells were fixed in 4% PFA for 20 min at room temperature. The cells were then blocked with 1% bovine serum albumin (BSA) containing 1% Triton X-100 for 1 h to prevent non-specific binding. Following blocking, the cells were incubated overnight at 4 °C with primary antibodies: anti-USP1 (Proteintech, Cat#1434-1-AP, 1:1000 v/v), anti-PLK1 (Cell Signaling, Cat#208G4, 1:500 v/v), anti-STUB1 (Santa Cruz, Cat#sc133066, 1:500 v/v). After three washes with PBS, the cells were incubated with fluorophore-conjugated secondary antibodies (ABclonal Technology) for 2.5 h at room temperature. The nuclei were stained using an antifade mounting medium containing DAPI (Beyotime Biotechnology). Images were captured using a confocal laser scanning microscope (ZEISS) or fluorescence microscope (OLYMPUS DP72). Pearson’s correlation coefficient (PCC) and Manders’ overlap coefficient (MOC) were calculated in ZEN (Zeiss) from raw *.czi images.

### Flow cytometry analysis

After treatment, cells were collected at a density of 1 × 10^6 cells/mL and washed with PBS. The cells were then fixed in 70% ethanol for at least 2 h. Following fixation, 100 µL of RNase A was added, and the cells were incubated at 37 °C for 30 min to remove RNA. Subsequently, the cells were incubated with propidium iodide (PI) solution for 30 min in the dark. The samples were analyzed by flow cytometry (BD Biosciences, USA).

### Tumor xenografts in nude mice

All procedures involving animals complied with institutional, national, and international guidelines and were approved by the Animal Care and Use Committee of Nantong University (approval No. P20230221-017; date:02/21/2023). The Committee acts in accordance with the International Council for Laboratory Animal Science (ICLAS) Ethics and Animal Welfare Committee guidelines, approved by the ICLAS Governing Board on 6 June 2013. Animals were housed under SPF. Anesthesia and humane endpoints were implemented as described. Reporting follows ARRIVE 2.0 recommendations where applicable. Four-week-old male BALB/c nude mice (20 g) were obtained from the Animal Research Center of Nantong University. The mice were randomly divided into following groups: DMSO, ML323 / NTUZLB-001, Lenvatinib, and ML323 / NTUZLB-001 + Lenvatinib. 5 × 10^6^ HCCLM3 or Huh7-R cells were injected subcutaneously into the dorsal flank of mice. Drug treatments were initiated on day 5. Mice were treated with Lenvatinib (4 mg/ kg) via oral gavage (p.o.) every 3 days. ML323 (4 mg/kg) or NTUZLB-001 (4 mg/kg) were intraperitoneally (i.p.) administrated into indicated groups every 3 days. After 30 days, Tumor weight was measured following euthanasia via CO_2_ asphyxiation for 5 min, and tumor samples were subsequently collected for additional investigation. The tumor volume was calculated as V = (length × width²) / 2.

### Immunohistochemical (IHC) staining

Tissue samples were fixed and embedded using standard techniques. The slides were sequentially immersed in xylene, 100% ethanol, 95% ethanol, 90% ethanol, 80% ethanol, and 70% ethanol to deparaffinize the tissue sections. The slides were then incubated in pre-heated sodium citrate buffer for 4 min to retrieve the antigens and subsequently blocked with 1% BSA solution. Following blocking, the slides were incubated overnight at 4 °C with primary antibodies. The immunohistochemistry assay was then performed using an IHC kit (Absin, abs957) according to the manufacturer’s instructions.

### Bioinformatics analyses

DeepMap-Drug sensitivity analysis was performed using the cSurvival database [22]. Liver cancer cell lines from DepMap/CCLE were selected as the analysis background. Lenvatinib response was queried using the DepMap compound identifier LENVATINIB (BRD: BRD-K39974922-001-04-3), and USP1 expression was evaluated using USP1 (Entrez ID: 7398). Cell lines were stratified into high- and low-USP1 expression groups using the dynamic iteration method (start percentile 0.2, end percentile 0.8, step size 0.1), with the final cutoff selected at the 70th percentile as implemented in cSurvival. Drug sensitivity was quantified as the log fold-change of cell viability relative to DMSO, and significance was assessed using the Wilcoxon rank-sum exact test with permutation-based adjustment (100 permutations).

Candidate E3 ligases for PLK1 were predicted using UbiBrowser [23]. Briefly, PLK1 was queried in the “Substrate” mode by entering the human gene symbol PLK1 (UniProt accession P53350) through the UbiBrowser web interface(ubibrowser.bio-it.cn/ubibrowser/). Predicted E3–substrate interactions were retrieved from the “predicted E3 ligases” output and filtered based on the built-in confidence score. To enhance stringency, candidates with confidence score ≥ 0.70 were prioritized for downstream analysis. Based on this filtering strategy, STUB1 was selected as a high-confidence candidate and subsequently subjected to experimental validation.

Public HCC transcriptomic datasets were collected from TCGA-LIHC, ICGC-LIRI-JP, ArrayExpress (E-TABM-36), and GEO cohorts (GSE10141, GSE104310, GSE109211, GSE112790, GSE136247, GSE144269, GSE14520, GSE1750, GSE25097, GSE36376, GSE45436, and GSE76427). Within each cohort, samples were stratified into USP1-high and USP1-low groups according to the median USP1 expression level. Gene set enrichment analysis was performed using the corresponding MSigDB gene set collection. For each cohort, genes were pre-ranked by the differential expression statistic between USP1-high and USP1-low groups, and normalized enrichment scores (NES) and false discovery rates (FDR, Benjamini–Hochberg correction) were computed. The single-cell RNA-seq dataset GSE166635 was analyzed using a standard Seurat workflow. Low-quality cells were removed based on gene complexity and mitochondrial transcript proportion, followed by log-normalization, scaling, dimensionality reduction (PCA), and UMAP visualization. Cell types were annotated according to canonical marker genes and the original dataset annotation. Malignant cells were extracted for downstream analyses. USP1-positive and USP1-negative malignant cell subsets were defined based on detectable USP1 expression in single cells, and differential gene expression was assessed between the two subsets. Functional enrichment analyses were conducted on significantly altered genes using pathway enrichment methods (GO/KEGG), with Benjamini–Hochberg adjustment for multiple testing.

### General simulation setup and parameterization

The protein or protein complex was placed in a cubic simulation box with periodic boundary conditions. Water molecules were explicitly modeled using the TIP3P water model, and Na + and Cl– ions were added to neutralize the system and maintain a physiological salinity of 150 mM. The system was parameterized using the Amber99SB*-ILDN force field, which incorporates improvements over Amber99 [24–26], while small molecules were parameterized with the General Amber Force Field (GAFF) [27]. Equilibration was performed using Desmond (version 2023.4) on a GPU, following a mixed NVT/NPT schedule. Production MD simulations were conducted in the NPT ensemble at a temperature of 310 K and a pressure of 1.013 bar. Temperature and pressure were controlled using the Nosé–Hoover thermostat and the Martyna–Tobias–Klein barostat, respectively [28]. Simulations employed a multigrator approach with a modified r-RESPA integrator, allowing a time step of 2 fs while evaluating long-range electrostatics every three steps. All simulations were executed on an NVIDIA RTX-4090 GPU.

### Preparation of protein/complex

To investigate the interaction between USP1 and PLK1, the crystal structure of PLK1 (PDB ID: 3HIK, chain A) [29] was used to capture the functional dynamics of its Polo-box domain (PBD). USP1 was modeled based on its catalytic conformation (PDB ID: 7AY1, chains C and D). MD simulations were performed to ensure structural convergence under physiologically relevant conditions. Structural gaps were resolved using AlphaFold3 predictions, and the termini were capped with acetyl (ACE) and N-methylamide (NME) groups. Following a 500-ns MD simulation, the most stable conformations were identified and subjected to docking using ZDOCK 3.0.2f [30] with IRaPPA [31] applied for re-ranking. Spatial constraints were introduced to maintain the proximity of K492 in PLK1 to the catalytic residues of the USP1-ubiquitin complex. Subsequently, a covalent linkage was established between K492 in PLK1 and G76 in ubiquitin. An additional 500-ns MD simulation was performed to refine the complex and capture its dynamic behavior.

### Virtual screening

The initial geometries of 1.6 million small molecules from the ChemDiv database were optimized using OpenBabel (version 3.1.0) [32]. The GAFF was chosen for its compatibility with organic molecules and its seamless integration with the Amber force field used for receptor proteins. Optimization was conducted using the Conjugate Gradient method, capped at 2500 steps, with a convergence criterion of energy changes below 10 − 7 kcal/mol. Additionally, all ligands were processed using Meeko to ensure consistency and compatibility for downstream docking and analysis. Protein interface conformations were extracted from MD trajectories, and binding pockets were defined using AutoDockTools with optimized grid parameters. The Uni-Dock [33] framework was employed for virtual screening, enabling efficient and accurate analysis of the ChemDiv library. From the initial screening, the top 5% of compounds (96,205 molecules) were selected based on docking scores. These were further refined using Uni-GBSA [34], which included energy minimization and scoring protein-ligand complexes with gmx_MMGBSA.py. The top 1% of compounds (962 molecules) underwent short 10-ns MD simulations, after which binding energies were re-evaluated using gmx_MMGBSA.py [35]. Combining automated scoring with manual inspection of binding modes, 46 candidate compounds were identified for purchase and experimental validation.

### In vitro USP1 deubiquitinase activity assay

The assay was conducted in a 384-well black plate with a final reaction volume of 10 µL. Test compounds (NTUZLB-001, ML323, and KSQ-4279) were first prepared as 10 mM stocks in 100% DMSO. For the assay, a 50 µM working solution was prepared in 100% DMSO, followed by a 5-fold serial dilution series in 100% DMSO to generate a concentration gradient. Subsequently, 10 µL of each DMSO dilution was transferred to a new plate and mixed with 90 µL of deionized water, resulting in a compound solution in 1% DMSO. Finally, 2 µL of this solution was dispensed into the assay plate, yielding a final DMSO concentration of 2% in the reaction. The final tested compound concentrations ranged from 10,000 nM to 0.64 nM.

The 2.5x enzyme solution was prepared in 1x assay buffer (50 mM HEPES, pH 7.8, 0.01 mg/mL BSA, 1 mM EDTA, 1 mM DTT, 20 mM NaCl, 0.01% Tween-20) to achieve a final USP1/UAF1 complex concentration of 0.3 nM in the reaction. After adding 4 µL of the enzyme solution to the compound-containing wells, the plate was shaken, centrifuged briefly, and pre-incubated for 20 min at room temperature. The enzymatic reaction was initiated by adding 4 µL of a 2.5x substrate solution containing ubiquitin-Rho 110 (final concentration: 125 nM). The reaction was allowed to proceed for 2 h at room temperature before being immediately analyzed.

Fluorescence was measured on an EnVision multimode plate reader (PerkinElmer) using dynamic mode with excitation at 480 nm and emission at 540 nm. Data were normalized: the signal from wells with 2% DMSO (no compound) was defined as 100% activity (negative control), and the signal from wells containing all components except the enzyme (blank control) was defined as 0% activity. Percent inhibition was calculated using the formula: Inhibition (%) = 100 - [(Sample - Blank) / (DMSO control - Blank)] ×100. Dose-response curves were fitted, and half-maximal inhibitory concentration (IC_50_) values were calculated using GraphPad Prism software.

### Statistical analysis

All statistical analyses were performed using GraphPad Prism 9.0 (CA, USA). A Student’s t-test was used to compare two independent groups, while a two-way ANOVA was applied for comparison of multiple groups. Differences were considered statistically significant when the p-value was less than 0.05.

## Results

### USP1 correlates with Lenvatinib sensitivity in HCC

**Fig. 1:**
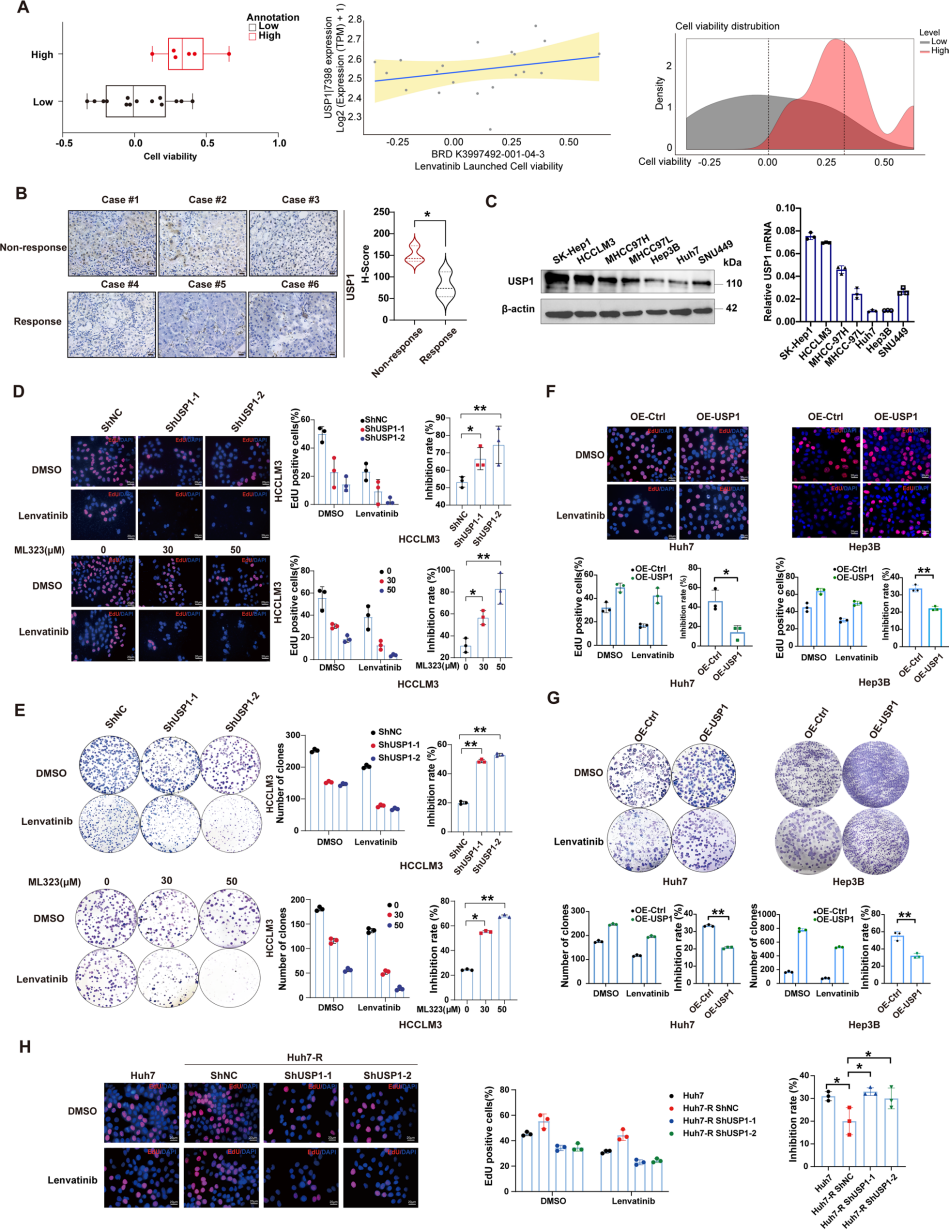
USP1 modulates Lenvatinib sensitivity in hepatocellular carcinoma.** A** Survival rates of hepatoma cell lines exhibiting high or low USP1 expression from the DeepMap-Drug database. **B** Immunohistochemical analysis of USP1 expression in liver tissue samples from patients responsive and non-responsive to Lenvatinib. USP1 staining was quantified by H-score. **C **Quantification of USP1 protein and mRNA levels across various hepatoma cell lines. **D **&** E** Lenvatinib (10 µM) inhibition rates in ML323-treated (30/50 µM) or USP1-depleted HCCLM3 cells, as assessed by EdU incorporation and colony formation assays, respectively. **F **&** G** Lenvatinib inhibition rates in USP1-overexpressing Huh7 and Hep3B cells, determined by EdU incorporation and colony formation assays, respectively. **H** Lenvatinib inhibition rates in USP1-silenced Lenvatinib-resistant Huh7 cells (Huh7-R), measured by EdU incorporation assay. **, *P* < 0.01; *, *P* < 0.05

Based on our previous studies, USP1 is hypothesized to play a role in HCC progression and malignancy [36]. In this study, we further evaluated its role in Lenvatinib resistance. The DeepMap-Drug database indicated that hepatoma cells with elevated USP1 expression exhibited increased survival rates following Lenvatinib treatment, suggesting that USP1 may be involved in Lenvatinib resistance (Fig. 1A). IHC assays on human HCC tissues revealed lower USP1 expression in patients who responded to Lenvatinib compared to non-responders (Fig. 1B). Variable USP1 expression levels were observed across different HCC cell lines at both the protein and mRNA levels Fig. 1C. For functional analyses, HCCLM3 and SK-Hep1 cells, with higher USP1 expression, were selected for loss-of-function studies, while Huh7 and Hep3B cells were used for gain-of-function experiments (Figure S1A). USP1 knockdown using two specific shRNAs significantly enhanced Lenvatinib’s efficacy in reducing malignant behaviors in HCC cells. Similarly, treatment with ML323, a known USP1 inhibitor, significantly enhanced the inhibitory effect of Lenvatinib on HCC cells in a dose-dependent manner (Fig. 1D&E, Figure S1B-F). In contrast, USP1 overexpression in Huh7 and Hep3B cells demonstrated the opposite effect, attenuating Lenvatinib’s efficacy (Fig. 1F&G, Figure S1G). To further validate the role of USP1 in Lenvatinib resistance, we established lenvatinib-resistant cell Huh7-R (FigureS1H). Consistently, immunoblotting showed that USP1 protein level was elevated in Huh7-R cells relative to Huh7 cells, and USP1 showed minimal change upon short-term lenvatinib treatment in the resistant cells. In contrast, USP1 decreased in HCCLM3 cells upon lenvatinib exposure (FigureS1I). Importantly, USP1 knockdown further enhanced Lenvatinib’s anti-proliferative effect on these resistant cells (Fig. 1H). We then generated the wild-type and deubiquitinating-inactive mutant C90S. Compared to the wild-type (WT), the C90S mutation failed to confer Lenvatinib resistance (Figure S2A-C). Collectively, these findings suggest that depleting USP1 could enhance Lenvatinib sensitivity in HCC cells, highlighting its potential as a therapeutic target.

### USP1 deletion induces mitotic arrest and chromosome misalignment

**Fig. 2:**
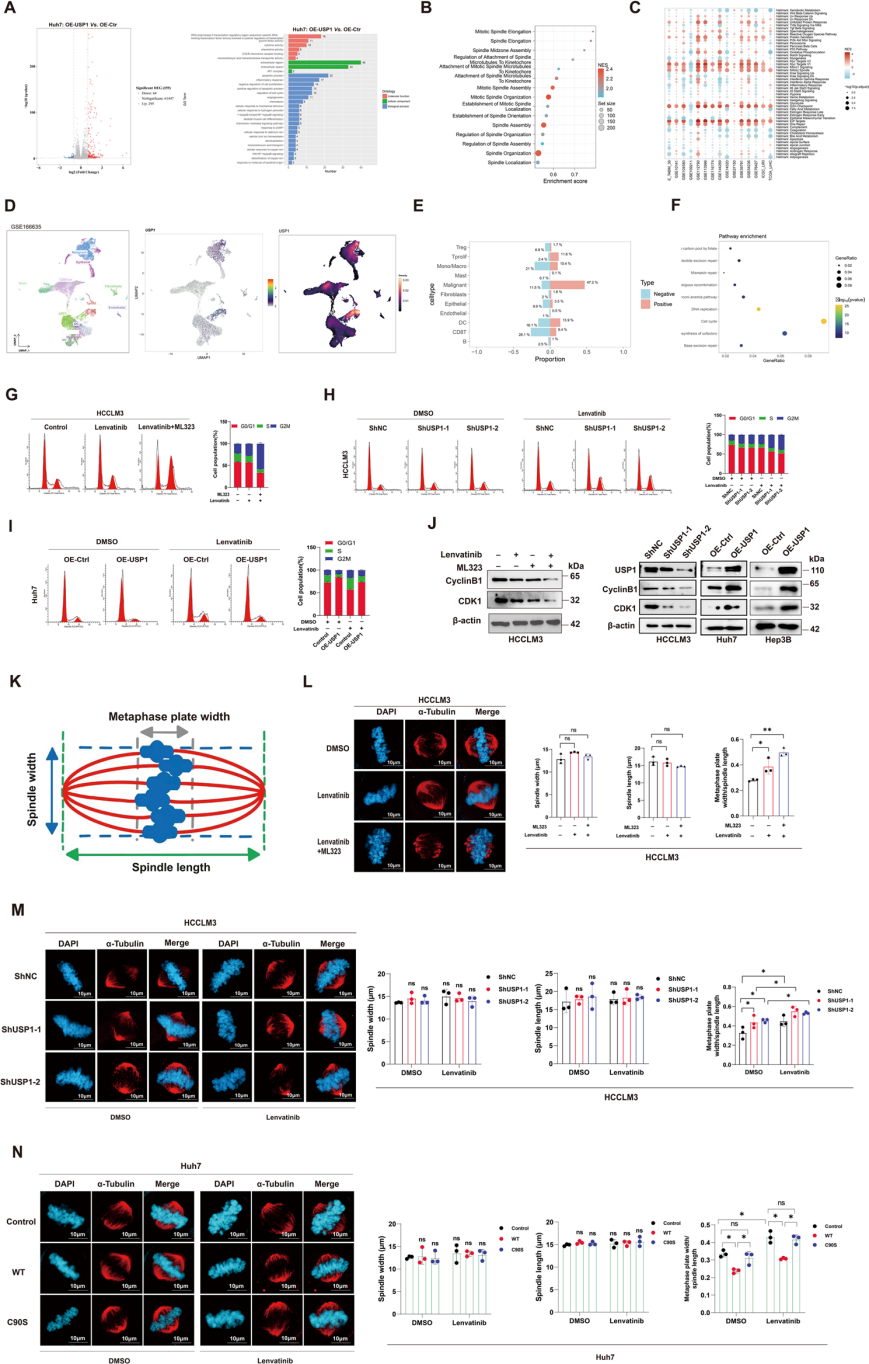
USP1 deletion induces mitotic arrest and chromosome misalignment.** A** RNA sequencing was performed to identify differentially expressed genes between control and USP1-overexpressing Huh7 cells. Gene Ontology (GO) enrichment analysis was subsequently conducted on the differentially expressed genes. **B** Gene Set Enrichment Analysis (GSEA) of USP1-high expression samples from the TCGA database. **(C)** GSEA of USP1-high expression samples from multiple datasets based on Hallmark gene sets. **D** Single-cell sequencing dataset was analyzed to define the expression characteristics of USP1 in single-cell sequencing dataset GSE166635. **E** The cell type differences of USP1 + and USP1- groups in GSE166635. **F **Enrichment analysis for USP1 + and USP1- malignant cells in single-cell sequencing dataset GSE166635. **G-I** Cell cycle distribution in various groups, analyzed by flow cytometry. **J** Immunoblot analysis of Cyclin B1 and CDK1 expression in USP1-knockdown, USP1-overexpressing (OE-USP1), and HCC cells treated with Lenvatinib (10 µM) plus ML323 (30 µM) compared to Lenvatinib (10 µM) alone. **K-N** Chromosome misalignment observed in HCC cells, pre-synchronized to metaphase. α-Tubulin is stained red and DAPI is stained blue. **, *P* < 0.01; *, *P* < 0.05

To explore the potential mechanism by which USP1 regulates Lenvatinib resistance, we performed RNA sequencing to identify the potential processes mediated by USP1 overexpression in USP1-overexpressed Huh7 cells. As shown in Fig. 2A, the differentially expressed genes were enriched in various pathway and processes, including cell proliferation, cell cycle and the inflammation response. Pathway enrichment analysis using GSEA was conducted on the TCGA LIHC dataset. The analysis revealed significant enrichment of mitotic pathways, including Mitotic Spindle Organization, Spindle Assembly, and Spindle Organization, in samples with high USP1 expression (Fig. 2B). The enrichment analyses of multiple datasets, based on Hallmark gene sets, suggested a possible role for USP1 in modulating Mitotic Spindle and G2M phase in HCC (Fig. 2C). Additionally, single-cell sequencing indicated that USP1 may mainly influence the proportion of malignant cells (Fig. 2D&E). Further enrichment analysis in USP1+/ USP1- malignant cells suggested that USP1 might be correlated with cell cycle (Fig. 2F). We then assessed changes in cell cycle distribution following USP1 interference or overexpression. Cell cycle analysis in HCC cells treated with Lenvatinib revealed significant accumulation in the G2/M phase, an effect that was enhanced by the USP1 inhibitor ML323 (Fig. 2G, Figure S3A). USP1 knockdown further increased lenvatinib-induced G2/M accumulation, whereas USP1 overexpression reduced the G2/M fraction and partially restored cell-cycle progression under lenvatinib exposure (Fig. 2H&I, Figure S3B). Protein levels of G2/M phase markers, Cyclin B1 and CDK1, were altered in response to USP1 modulation and ML323 treatment, supporting their involvement in the observed cell cycle effects (Fig. 2J, Figure S3C). Previous studies have shown that chromosome misalignment can lead to mitotic catastrophe in HeLa and H1299 cells [37]. Therefore, to further explore the relationship between Lenvatinib and G2/M phase arrest, we stained the spindle and chromatin with α-tubulin and DAPI after synchronizing cells in metaphase. Lenvatinib treatment resulted in noticeable chromosome misalignment, a condition exacerbated by USP1 inhibition. Chromosome misalignment induced by lenvatinib was rescued by USP1 WT, but not by the catalytic-inactive C90S mutant, supporting a catalytic activity–dependent role of USP1 in maintaining metaphase chromosome alignment (Fig. 2K-N, Figure S3D&E). These data demonstrate that USP1 regulates G2/M progression and chromosome alignment under lenvatinib stress, thereby facilitating lenvatinib resistance in HCC cells.

### Identify PLK1 as a key substrate of USP1

**Fig. 3:**
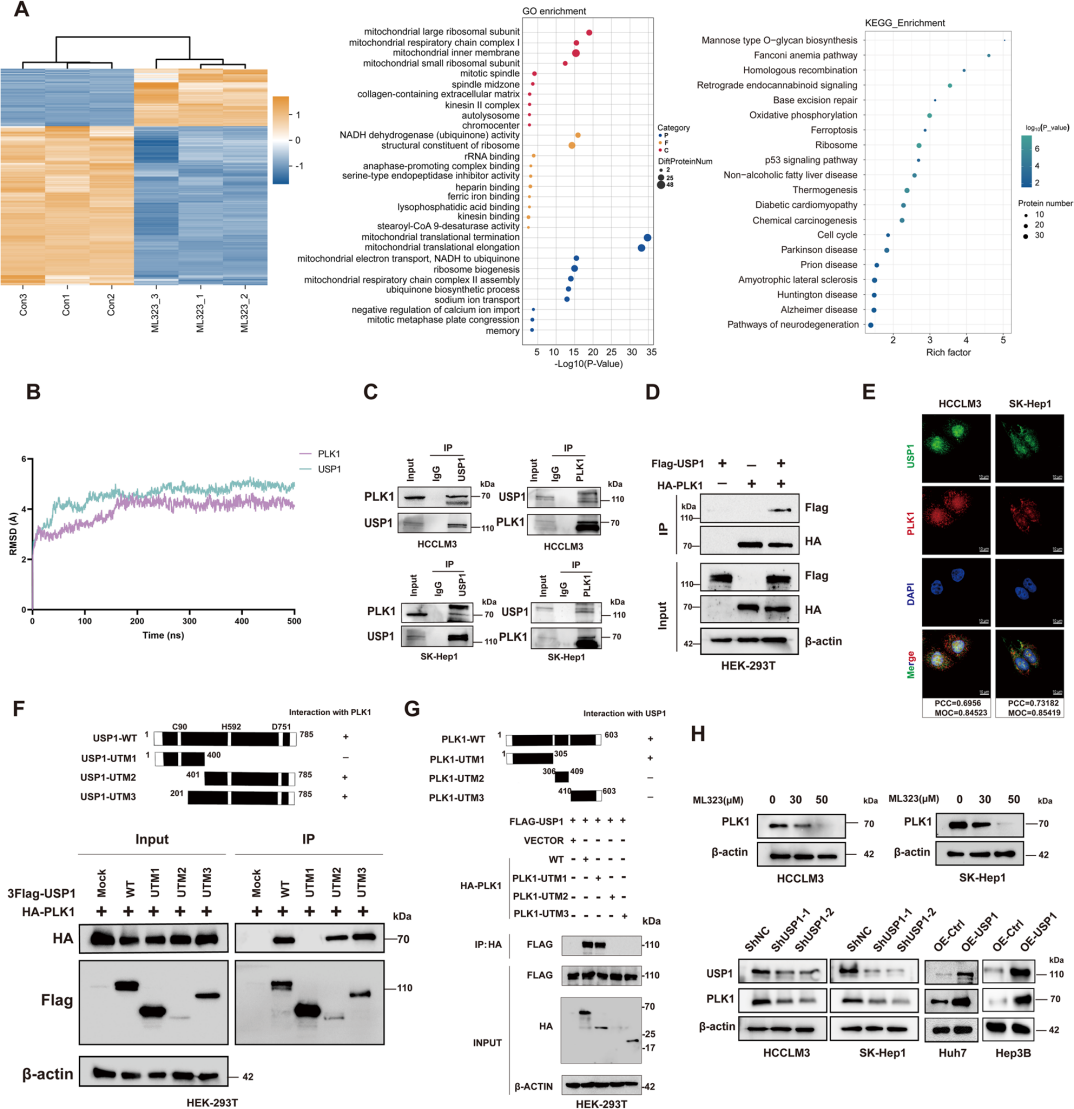
USP1 interacts with and regulates PLK1.** A** Mass spectrometry analysis of control and ML323-treated cells to identify differentially expressed proteins. **B** A 500 ns molecular dynamics simulation was conducted on the complex, with Root Mean Square Deviation (RMSD) analyses performed for both PLK1 (magenta) and USP1 (cyan). **C** Co-immunoprecipitation assays validating the endogenous interaction between USP1 and PLK1 in HCC cells. **D** Exogenous Co-immunoprecipitation assays of 3Flag-tagged USP1 and HA-tagged PLK1 co-expressed in HEK293T cells. **E** Co-localization of USP1 and PLK1 in HCC cells. Colocalization was presented as Pearson’s correlation coefficient (PCC) and Manders’ overlap coefficient (MOC). **F** Interaction between full-length PLK1 and USP1-truncated mutants, assessed by co-immunoprecipitation and immunoblotting using the indicated antibodies. **G** Interaction between full-length USP1 and PLK1-truncated mutants, assessed by co-immunoprecipitation and immunoblotting with the indicated antibodies. **H** Immunoblot analysis of PLK1 expression in HCC cells with ML323 treatment, USP1 knockdown or USP1 overexpression

As is known, the biological activities of DUBs are largely dependent on their substrates. Mass spectrometry (MS) was performed to identify significantly downregulated proteins (fold change > 1, *P* < 0.05) upon ML323 treatment (Fig. 3A, Figure S4A&B). UbiBrowser was used to predict potential substrates. Four proteins (PLK1, HELLS, PRC1, and KDM1) were subsequently identified as candidates based on the combined analysis, with PLK1 frequently associated with cell cycle regulation and mitosis. The DeepMap-Drug database further indicated that high PLK1 protein expression was associated with a higher survival rate following Lenvatinib treatment (Figure S4C). Thus, PLK1 was selected for further validation. Molecular dynamics (MD) simulations over 500 ns confirmed the structural stability of the USP1-PLK1 complex, as supported by RMSD analyses **(**Fig. 3B**)**. Notably, the binding interface between USP1 and PLK1 aligns with the previously reported interaction site of USP1 and FANCI [38], underscoring its potential role as USP1’s substrate recognition and binding domain. Co-IP assays confirmed that endogenous USP1 interacts with PLK1 in HCC cells **(**Fig. 3C**)**. The interaction between USP1 and PLK1 was further validated in HEK293T cells via co-transfection of Flag-USP1 and HA-PLK1 (Fig. 3D). Immunofluorescence staining revealed the co-localization between USP1 and PLK1 (Fig. 3E). Next, we generated three truncated mutants (UTMs) of USP1 to identify the domains required for binding to PLK1. The UTMs include the N-terminal USP1:UTM1 (1-400aa) encoding the catalytic cysteine (Cys) box, the C-terminal USP1:UTM2 (401-785aa) encoding the catalytic histidine (His) box and aspartic acid (Asp) box, and the extended C-terminal USP1:UTM3 (201-785aa) encoding both the His and Asp catalytic boxes. Co-IP results demonstrated that UTM2 and UTM3 are essential for binding to PLK1 **(**Fig. 3F**)**. Additionally, we generated three UTMs of PLK1 and identified the interaction between PLK1 truncated mutants (1-305aa) and USP1 **(**Fig. 3G**)**. To determine how USP1 regulates PLK1, we assessed the protein and mRNA levels of PLK1 using western blot and qRT-PCR. As shown in Fig. 3H and Figure S4D-F, USP1 positively modulated the protein expression of PLK1. However, no significant changes were observed at the mRNA level following this treatment. These results suggest that USP1 interacts with PLK1 and regulates its protein expression at the post-transcriptional level.

### USP1 stabilizes PLK1 via deubiquitination

**Fig. 4:**
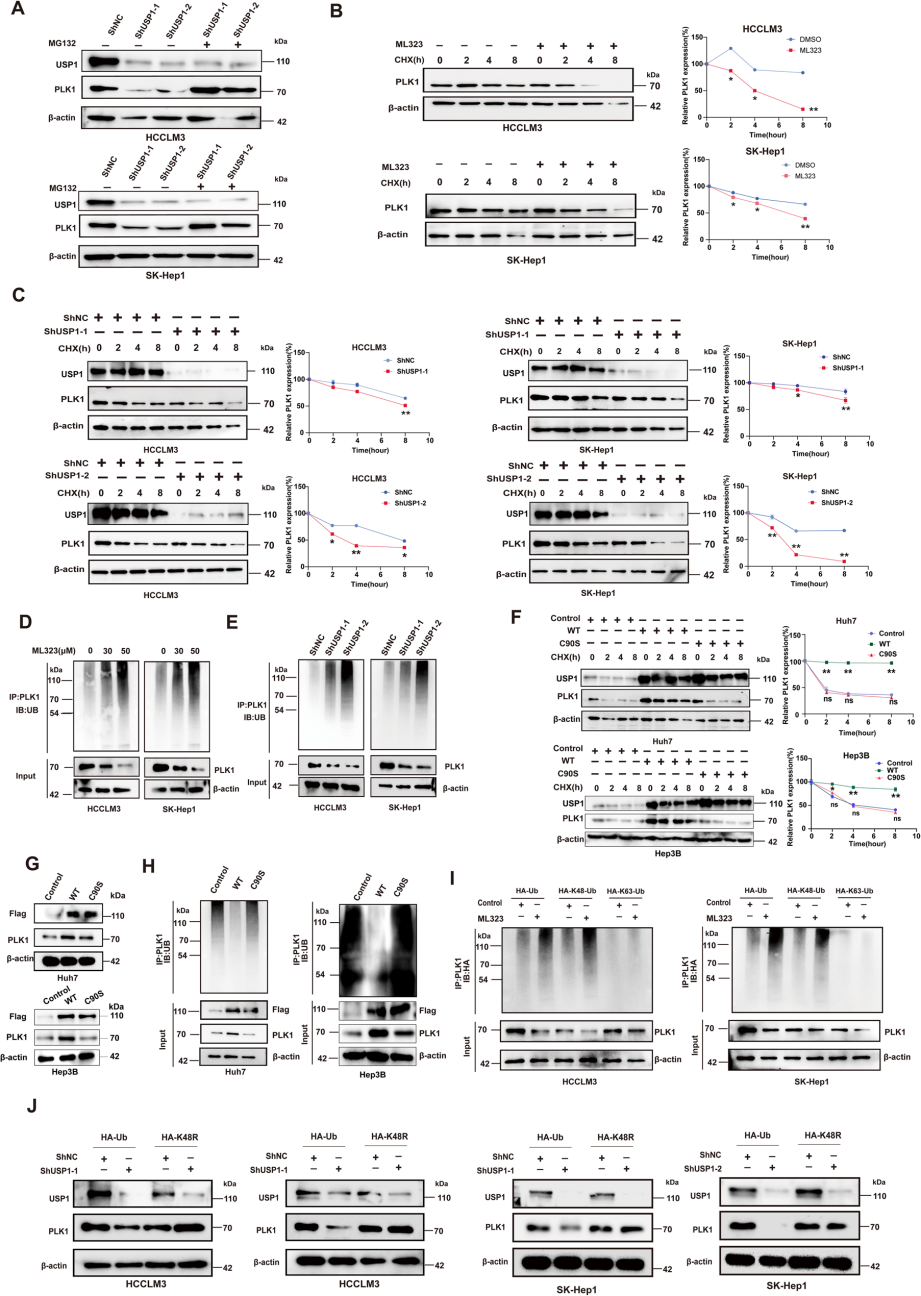
USP1 stabilizes PLK1 through deubiquitination.** A** Immunoblot analysis of PLK1 expression in HCC cells subjected to different treatments. **B**&**C** PLK1 expression in HCC cells treated with cycloheximide (CHX) at the indicated time points. Quantification of PLK1 levels was performed relative to β-actin. **D**&**E** Ubiquitination levels of PLK1 were analyzed by immunoprecipitation using an anti-PLK1 antibody, followed by immunoblotting with an anti-ubiquitin antibody. **F **PLK1 expression in HCC cells treated with CHX at indicated time points in the Control, WT, and C90S groups. **G** Immunoblot analysis of Flag-tagged USP1 and PLK1 expression in Huh7 and Hep3B cells transfected with Control, WT, and C90S plasmids. **H** Ubiquitination levels of PLK1 in Huh7 and Hep3B cells transfected with Control, WT, and C90S plasmids. **I** HCC cells treated with ML323 and transfected with HA-Ub, HA-K48-Ub, or HA-K63-Ub plasmids. Then PLK1 ubiquitination linkage was analyzed. **J** HCC cells transfected with HA-Ub or HA-K48-Ub in the presence of ShNC or ShUSP1-1/2. Cell lysates were analyzed by immunoblotting using anti-PLK1 and anti-USP1 antibodies

Based on the previous results, we hypothesized that USP1 regulates PLK1 expression through the ubiquitin-proteasome pathway. Consistent with this hypothesis, the proteasome inhibitor MG132 rescued PLK1 expression, which was decreased by USP1 knockdown (Fig. 4A). To assess whether USP1 promotes PLK1 stability, we treated the cells with cycloheximide (CHX). ML323 treatment or USP1 depletion significantly accelerated PLK1 protein degradation upon CHX treatment **(**Fig. 4B&C**)**. As shown in Fig. 4D, endogenous ubiquitination of PLK1 was upregulated in cells treated with ML323. Similar results were observed in the USP1-knockdown group **(**Fig. 4E**)**. In contrast, wild-type USP1, but not the C90S mutant, significantly enhanced PLK1 stability **(**Fig. 4F**)**. Furthermore, compared to the wild-type USP1, the C90S mutant failed to significantly upregulate PLK1 expression at the protein level **(**Fig. 4G**)**. Consistent with this, overexpression of wild-type USP1, but not the C90S mutant, reduced ubiquitination levels in Huh7 and Hep3B cells **(**Fig. 4H**)**. These results suggest that USP1 regulates PLK1 in a deubiquitination-dependent manner. Additional assays were performed to identify the specific ubiquitin chains involved in USP1-mediated deubiquitination. Our results indicate that USP1 efficiently removes the K48-linked ubiquitin chain from PLK1 **(**Fig. 4I&J**)**. Taken together, USP1 stabilizes PLK1 via deubiquitination in HCC cells.

### PLK1 mediated the USP1-driven Lenvatinib resistance

**Fig. 5:**
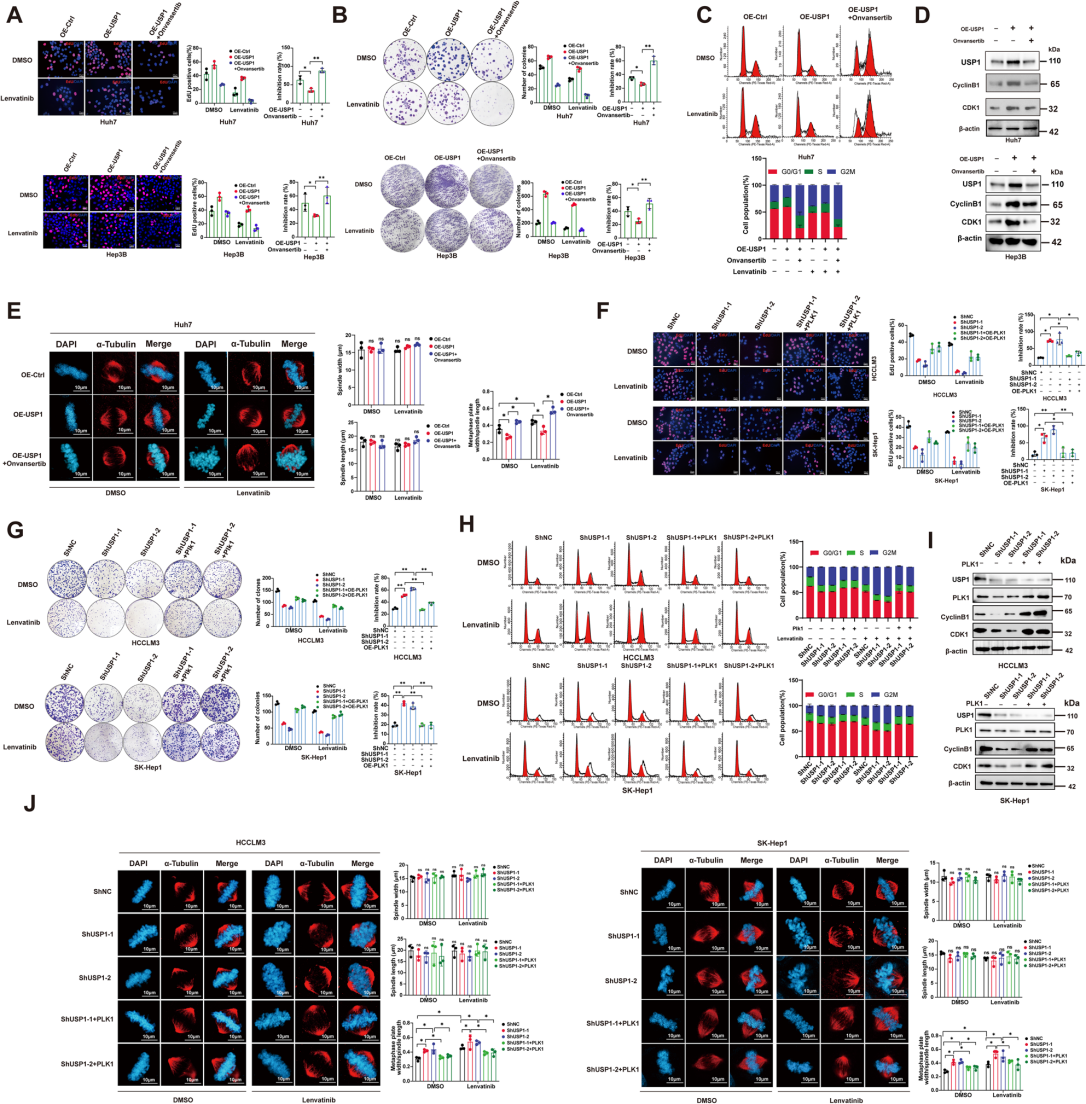
PLK1 mediated the USP1-driven Lenvatinib resistance. **A,B** Lenvatinib (10 µM) inhibition rates in Huh7 and Hep3B cells treated with Onvansertib (5 µM) following transfection with OE-USP1 plasmids, assessed by EdU incorporation and colony formation assays. **C**&**D** Cell cycle distribution and expression of cell cycle-related proteins in the aforementioned conditions, as analyzed by flow cytometry and immunoblotting. **E** Chromosome misalignment in Huh7 cells synchronized to metaphase, detected by immunofluorescence and confocal microscopy. **F**&**G** Lenvatinib inhibition rates in HCC cells co-transfected with ShUSP1 and OE-PLK1 plasmids, measured by EdU incorporation and colony formation assays. **H**&**I** Cell cycle distribution and expression of cell cycle-related proteins in HCCLM3 and SK-Hep1 cells under the aforementioned conditions, as analyzed by flow cytometry and immunoblotting. **J** Chromosome misalignment in HCC cells pre-synchronized to metaphase under the indicated conditions, detected by immunofluorescence. **, *P* < 0.01; *, *P* < 0.05

We investigated whether USP1-mediated PLK1 stabilization is essential for Lenvatinib resistance in HCC cells. Rescue experiments were performed by treating OE-USP1 cells with Onvansertib, a PLK1 inhibitor, in Huh7 and Hep3B cells. Onvansertib significantly enhanced cell sensitivity to Lenvatinib, which was impaired by PLK1 overexpression (Fig. 5A&B, Figure S5A&B). Additionally, Onvansertib increased the G2/M phase population and reduced the expression of Cyclin B1 and CDK1 (Fig. 5C&D). Consistent with the previous results, Onvansertib dramatically disrupted chromosome alignment compared to USP1 overexpression cells **(**Fig. 5E**)**. Furthermore, we overexpressed PLK1 in USP1-knockdown or ML323-treated cells. Consistently, PLK1 overexpression enhanced cell resistance to Lenvatinib, which was reduced by USP1 knockdown **(**Fig. 5F&G, Figure S5C-H). Additionally, ectopic PLK1 expression restored the G2/M phase population, and altered the expression of Cyclin B1 and CDK1 caused by USP1 inhibition (Fig. 5H&I). In line with the previous results, forced PLK1 expression in HCC cells significantly inhibited chromosome misalignment induced by USP1 downregulation (Fig. 5J). In summary, these results suggest that USP1 mediates Lenvatinib resistance in HCC cells through PLK1.

### The E3 ligase STUB1, together with USP1, regulated the equilibrium of PLK1

**Fig. 6:**
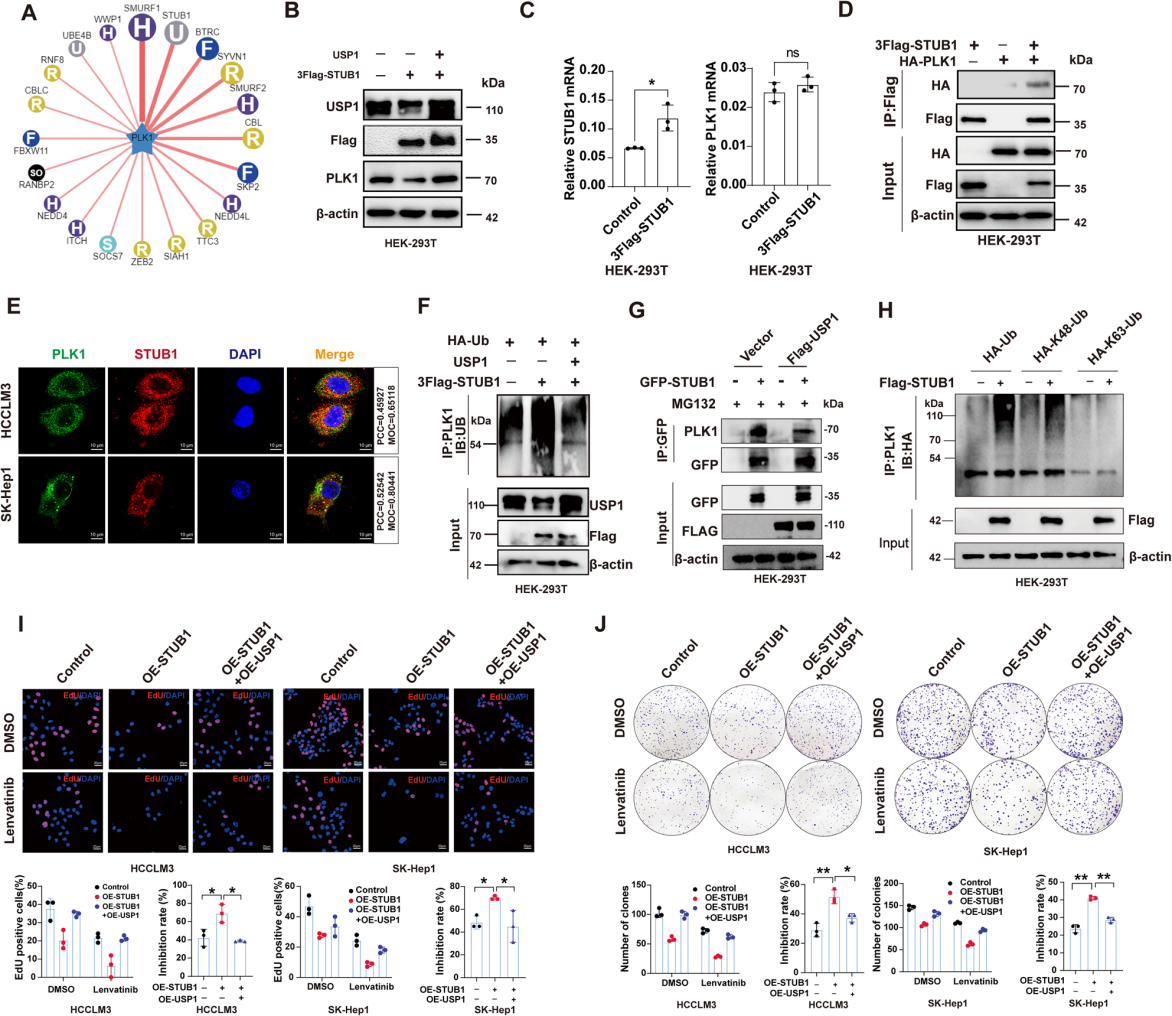
The E3 ligase STUB1, in conjunction with USP1, regulates PLK1 homeostasis.** A** Candidate E3 ligases of PLK1 predicted using the UbiBrowser database. **B** Immunoblot analysis of USP1, Flag, and PLK1 expression in HEK293T cells transfected with 3Flag-STUB1 alone or co-transfected with 3Flag-STUB1 and OE-USP1 plasmids. **C** mRNA levels of STUB1 and PLK1 in HEK293T cells transfected with 3Flag-STUB1. **D** Co-immunoprecipitation assays of 3Flag-tagged STUB1 and HA-tagged PLK1 co-expressed in HEK293T cells. **E** Co-localization of STUB1 and PLK1 in HCCLM3 and SK-Hep1. Colocalization was presented as Pearson’s correlation coefficient (PCC) and Manders’ overlap coefficient (MOC). **F** Ubiquitination levels of PLK1 in HEK293T cells transfected with 3Flag-STUB1 or with additional OE-USP1 plasmids. **G** Interaction between PLK1 and STUB1 in the presence of ectopic USP1, detected by Co-IP assay. **H** HCC cells transfected with Flag-STUB1 and transfected with HA-Ub, HA-K48-Ub, or HA-K63-Ub plasmids. Then PLK1 ubiquitination linkage was analyzed. **I**&**J** Lenvatinib (10 µM) inhibition rates in HCC cells transfected with 3Flag-STUB1 alone or co-transfected with 3Flag-STUB1 and OE-USP1 plasmids, measured by EdU staining and colony formation assays. **, *P* < 0.01; *, *P* < 0.05

Ubiquitination and deubiquitination are dynamic processes that maintain protein homeostasis. The ubiquitin database was utilized to predict the E3 ligase for PLK1 **(**Fig. 6A**)**. Western blot and qPCR results showed that the ubiquitin E3 ligase STUB1 regulates PLK1 expression at the protein level, but not at the mRNA level (Fig. 6B&C). The interaction between USP1 and PLK1 was validated in HEK293T cells by co-transfecting Flag-STUB1 and HA-PLK1 **(**Fig. 6D**)**. Immunofluorescence staining revealed the co-localization between STUB1 and PLK1 **(**Fig. 6E**)**. We then further confirmed whether STUB1 and USP1 participate in the regulation of PLK1 expression. Additionally, USP1 overexpression rescued the decrease in PLK1 caused by STUB1 overexpression. Ubiquitin-binding assays further confirmed that STUB1 overexpression increased the ubiquitination level, which was reversed by USP1 overexpression **(**Fig. 6F**)**. Additionally, ectopic USP1 expression reduced the interaction between STUB1 and PLK1 **(**Fig. 6G**)**. Further assays were conducted to identify the specific ubiquitin chains involved in STUB1-mediated ubiquitination. Our results suggest that STUB1 efficiently adds the K48-linked ubiquitin chain to PLK1 **(**Fig. 6H**)**. We further explored the effect of STUB1 on Lenvatinib resistance mediated by the USP1-PLK1 deubiquitination axis. EdU and colony formation assays showed that STUB1 enhanced Lenvatinib sensitivity in HCC cells, and this effect was reversed by USP1 overexpression **(**Fig. 6I&J**)**. In conclusion, USP1 and the E3 ligase STUB1 together maintain the equilibrium of PLK1, thereby regulating Lenvatinib resistance.

### PLK1 regulate USP1 by phosphorylating c-Myc

As shown above, USP1 efficiently regulated the expression of PLK1. We further investigated whether PLK1 modulates the expression of USP1. As shown in Figure S6A, PLK1 overexpression significantly enhanced the expression of USP1 at both the transcriptional and protein levels. It has been reported that PLK1 regulated c-Myc expression through phosphorylation at Ser62. As hypothesized, ectopic PLK1 upregulated both total expression and phosphorylation of c-Myc, whereas Onvansertib reduced c-Myc expression and decreased phosphorylation at the Ser62 site. As shown in Figure S6B, MYC was identified as the sole candidate transcription factor of USP1 across all six transcription factor databases. ChIP assays further confirmed the binding of c-Myc to the USP1 promoter (Figure S6C). Importantly, promoter-reporter assays suggested that c-Myc overexpression significantly enhanced USP1 promoter–driven luciferase activity in Huh7 and Hep3B cells, whereas the c-Myc inhibitor 10058-F4 suppressed USP1 promoter activity in HCCLM3 and SK-Hep1 cells (Figure S6D). In line with these findings, treatment with c-Myc inhibitor 10058-F4, downregulated USP1 expression at both protein and mRNA levels in a dose-dependent manner (Figure S6E&F). Based on the results above, we propose that c-Myc is a transcription factor of USP1, and that the USP1-PLK1-c-Myc axis forms a positive feedback loop in HCC cells.

### The ML323 in combination with Lenvatinib synergistically kills HCC cells in vitro and vivo

**Fig. 7:**
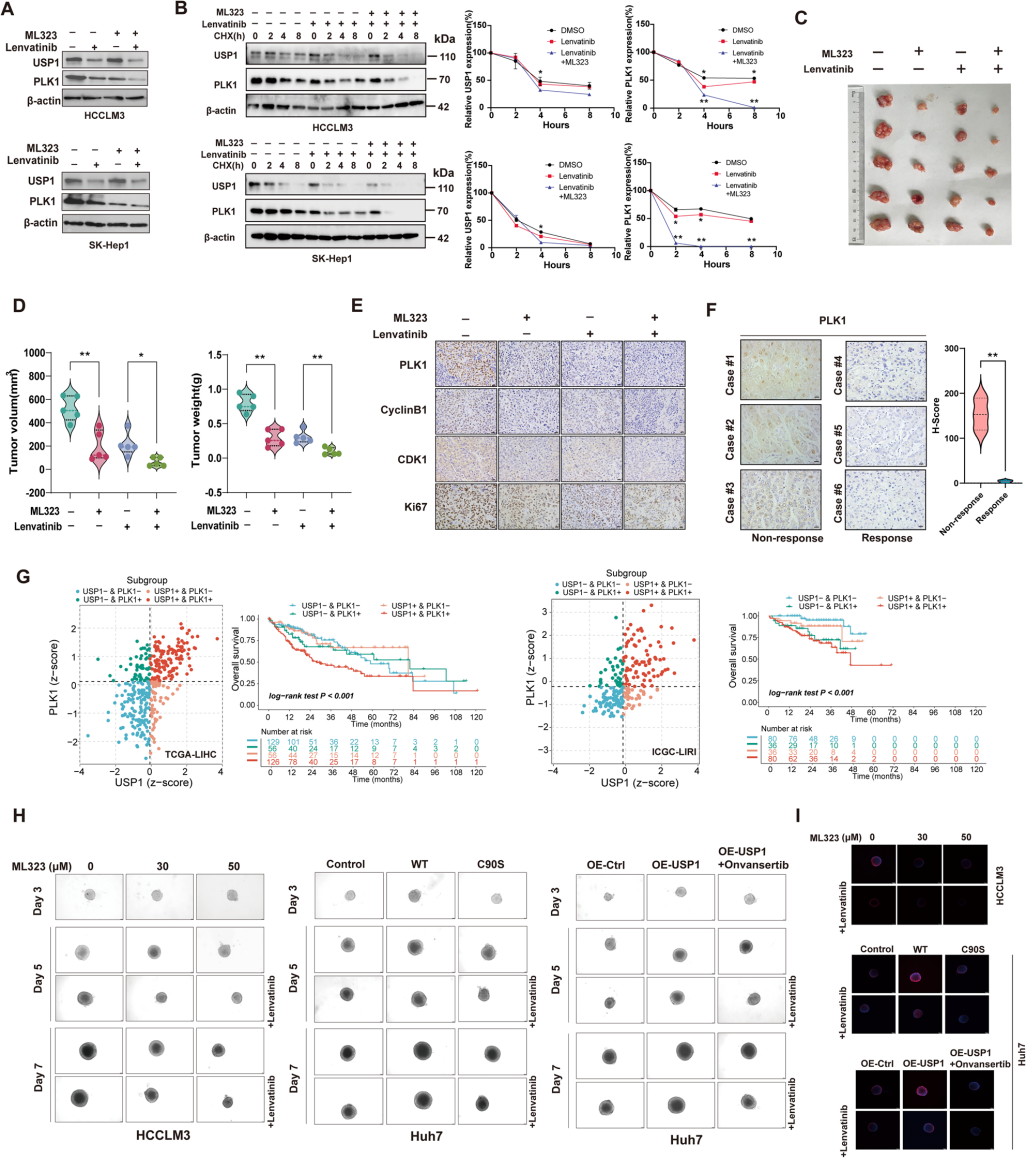
Pharmacological depletion of USP1 synergistically enhances Lenvatinib-mediated inactivation of HCC cells.** A** Immunoblotting analysis of USP1 and PLK1 expression in HCC cells treated with ML323 (30 µM), Lenvatinib (10 µM), or a combination of ML323 (30 µM) and Lenvatinib (10 µM). **B **Expression of USP1 and PLK1 in HCC cells treated with cycloheximide (CHX) at indicated time points in the different treatment groups. **C** HCCLM3 xenograft tumor-bearing mice were treated with ML323 or Lenvatinib alone, or with the combination of both drugs. DMSO was used as a control. Representative images of HCCLM3 xenograft tumors from each treatment group. **D** Tumor weight and volume measurements in each treatment group. **E** Representative images of immunohistochemical staining for PLK1, Cyclin B1, CDK1, and Ki-67 in xenograft tumor tissues. **F** Expression of PLK1 in Lenvatinib-responsive and non-responsive liver tissues. PLK1 staining was quantified by H-score. **G** The prognostic analysis was conducted in subgroups of USP1 + PLK1+, USP1 + PLK1-. USP1-PLK1+, and USP1-PLK1- stratified by Z-score. **H** Growth of 3D spheroids derived from HCCLM3 cells in each treatment group at days 3, 5, and 7. The spheroids were treated with ML323 (30 µM), Lenvatinib (10 µM), or a combination of both. **I** EdU staining of the 3D spheroids. **, *P* < 0.01; *, *P* < 0.05

Based on the aforementioned results, we found that ML323 enhances the inhibitory effect of Lenvatinib on the proliferation and migration of HCC cells. Thus, we aimed to determine whether the combination of Lenvatinib and ML323 could exert a synergistic effect on PLK1 expression. As shown in Fig. 7A, cells co-treated with Lenvatinib and ML323 exhibited lower PLK1 expression levels compared to those treated separately with Lenvatinib or ML323. CHX-tracking assays demonstrated that the combination of Lenvatinib and ML323 further promoted PLK1 protein degradation (Fig. 7B). To further confirm our findings in vivo, we performed subcutaneous tumor formation using HCCLM3 cells in nude mice. We divided the tumor-bearing nude mice into four groups: normal saline control, ML323 (4 mg/kg), Lenvatinib (4 mg/kg), and combination (Lenvatinib 4 mg/kg + ML323 4 mg/kg). No significant differences in weight were observed among the different groups of mice. As shown in Fig. 7C&D, compared with the control group, ML323 or Lenvatinib treatment resulted in smaller xenograft tumor volumes. However, the combination of Lenvatinib and ML323 demonstrated the most effective inhibitory effect on xenograft tumor growth. Immunohistochemical staining of xenograft tumor tissues showed that the expression levels of PLK1, Cyclin B1, CDK1, and Ki67 were lowest in the combination of Lenvatinib and ML323 group (Fig. 7E). IHC assays of human HCC tissues showed that PLK1 expression was lower in Lenvatinib-responsive patients than in non-responders (Fig. 7F**)**. Interestingly, joint-analysis indicated that patients exhibiting co-elevation of USP1 and PLK1 demonstrated significantly reduced overall survival (Fig. 7G**)**. The results from 3D models and EdU staining were consistent with the previous findings **(**Fig. 7H&I**)**. Taken together, these findings suggest that ML323, in combination with Lenvatinib, induces synergistic inhibitory effects on HCC cells both in vitro and in vivo.

### Screening and validating a novel USP1 inhibitor overcoming Lenvatinib resistance

**Fig. 8:**
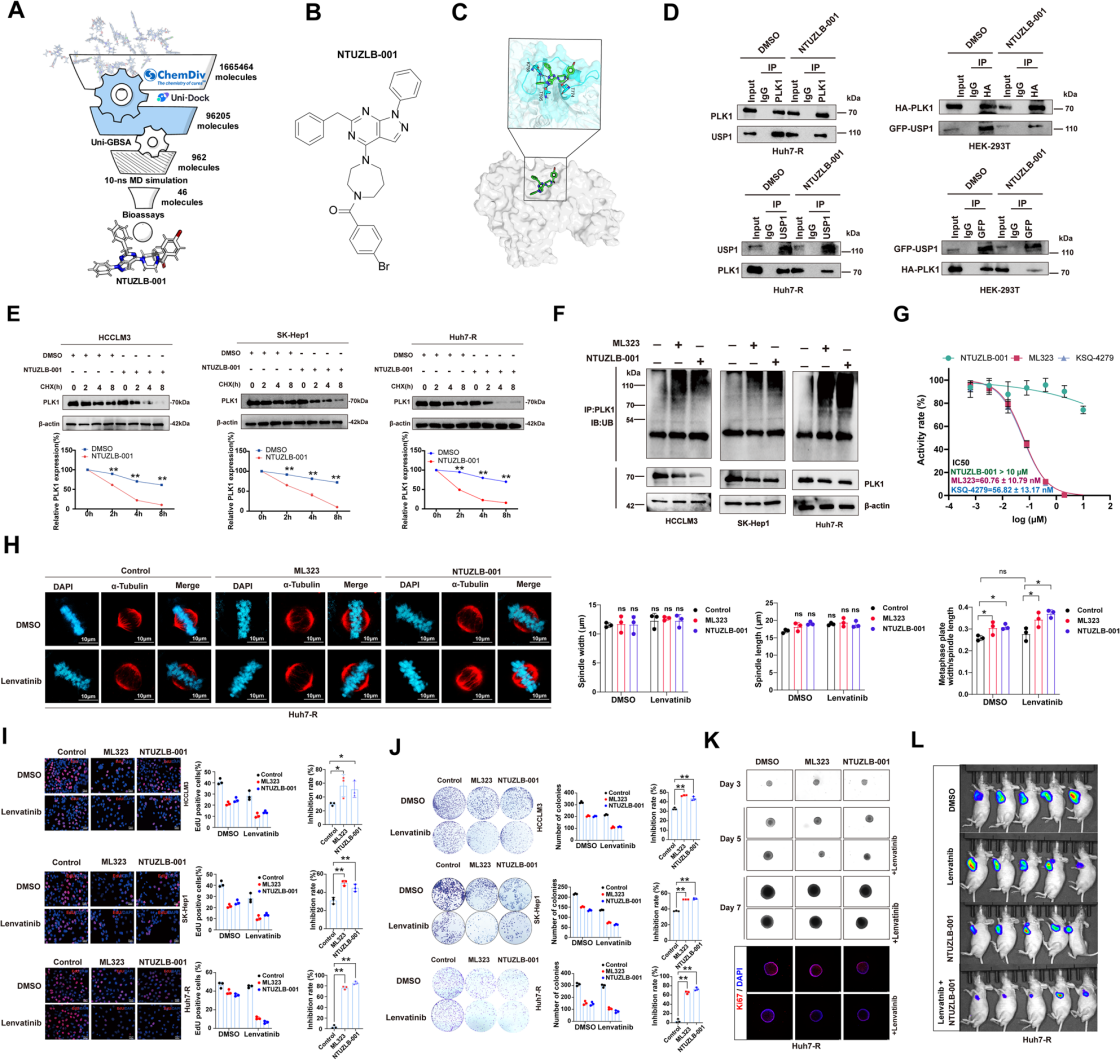
Identification of a novel inhibitor for USP1/PLK1 interaction.** A** Virtual screening workflow. Virtual screening was performed targeting the binding pocket of the USP1-PLK1 interaction interface, utilizing the GPU-based Uni-Dock and Uni-GBSA workflow. This process incorporated short MD simulations and manual inspection. From the ChemDiv library (1,665,464 molecules), 46 compounds were selected and validated through biological experiments, ultimately leading to the identification of NTUZLB-001. **B** Chemical structure of NTUZLB-001. **C** Binding mode of compound NTUZLB-001 at the USP1-PLK1 interaction interface. The compound’s carbon atoms are shown in green, while the carbon backbone of the protein surface residues is depicted in cyan. NTUZLB-001 forms stable hydrogen bonds with residues T774, T595, and K756 of USP1. **D** Co-immunoprecipitation assays validating the endogenous and exogenous interaction with NTUZLB-001 (30 µM) treatment between USP1 and PLK1 in Huh7-R and HEK-293T cells, respectively. **E** Expression of PLK1 in HCC cells, and Huh7-R cells treated with cycloheximide (CHX) at indicated time points. **F** Ubiquitination levels of PLK1 in HCC and Huh7-R cells treated with ML323 (30 µM) or NTUZLB-001 (30 µM). **G** In vitro USP1 deubiquitinase activity assay using a catalytic-site–dependent substrate, comparing NTUZLB-001 with the canonical USP1 inhibitor ML323 and the positive control KSQ-4279. **H** Chromosome misalignment in Huh7-R cells synchronized to metaphase, assessed by confocal staining, following treatment with ML323 or NTUZLB-001. **I**&**J** Synergistic inhibition of Lenvatinib (10 µM) in combination with ML323 (30 µM) or NTUZLB-001 (30 µM) in HCC cells and Huh7-R cells, assessed by EdU staining and colony formation assays. **K** Growth of 3D spheroids derived from Huh7-R cells treated with ML323 (30 µM), NTUZLB-001(30 µM), Lenvatinib (10 µM), or their combination, at day 3, 5, and 7, as well as EdU staining of spheroids. **L** In vivo imaging was utilized to monitor tumor growth in a Huh7-R subcutaneous tumor xenograft model treated with NTUZLB-001 or Lenvatinib alone, or with the combination of both drugs. **, *P* < 0.01; *, *P* < 0.05

To identify small molecules capable of disrupting the PLK1-USP1 interaction, we analyzed the binding interface, prioritizing the USP1 surface to preserve PLK1 functionality. Virtual screening of 1.6 million compounds from the ChemDiv library yielded 46 candidates for further molecular and cellular biology investigations **(**Fig. 8A**)**. Among these, NTUZLB-001 **(**Fig. 8B; Figure S7**)** emerged as a potent disruptor of the PLK1-USP1 interaction, significantly altering PLK1 stability and demonstrating strong biological activity. To explore the molecular basis of this activity, NTUZLB-001 was subjected to a 500 ns MD simulation in complex with USP1. Analysis showed that NTUZLB-001 established stable hydrogen bonds with residues T774, T595, and K756 of USP1. Furthermore, a cation-π interaction formed between the amino cation of K756 and the benzene ring of the compound, enhancing structural stability and anchoring the molecule at the protein-protein interface. This interaction successfully disrupted the binding of USP1 to PLK1, underscoring its potential as an effective modulator of PLK1 stability (Fig. 8C). As shown in Fig. 8D, NTUZLB-001 reduced the endogenous and exogenous interaction between USP1 and PLK1 in Huh7-R and HEK-293T cells, respectively. Additionally, NTUZLB-001 significantly accelerated CHX-induced degradation of PLK1 in HCC cells, and Lenvatinib-resistant Huh7-R cells **(**Fig. 8E**)**. Similar to ML323, NTUZLB-001 also enhanced PLK1 ubiquitination levels in HCC cells **(**Fig. 8F**)**. The in vitro deubiquitinase assay demonstrated robust inhibition of USP1/UAF1 by the canonical catalytic-site inhibitors ML323 and KSQ-4279, with IC50 values of 60.76 ± 10.79 nM and 56.82 ± 13.17 nM, respectively **(**Fig. 8G**)**. In contrast, NTUZLB-001 showed no measurable inhibition of USP1 enzymatic activity at concentrations up to 10 µM (IC50 > 10 µM). This result definitively establishes that the inhibitory effects of NTUZLB-001 on PLK1 are not mediated through direct pharmacological blockade of the USP1 catalytic pocket. For the phenotypes, combining NTUZLB-001 with Lenvatinib significantly induced chromosome misalignment in Lenvatinib-resistant Huh7-R cells **(**Fig. 8H**)**. Furthermore, in combination with Lenvatinib, NTUZLB-001 competitively inhibited proliferation, colony formation, and spheroid growth in HCC cells and Lenvatinib-resistant Huh7-R cells **(**Fig. 8I-K**)**. Consistently, NTUZLB-001 also enhanced the efficacy of Lenvatinib in inhibiting tumor growth in vivo **(**Fig. 8L**)**. Therefore, NTUZLB-001 could be a novel USP1-PLK1 inhibitor that exhibits synergistic inhibitory effects with Lenvatinib on HCC cells.

## Discussion

Accumulating evidence suggests that the processes of ubiquitination and deubiquitination are closely associated with drug resistance in various malignancies [39]. USP1 has been shown to promote proliferation and metastasis in HCC. Recently, USP1 was shown to enhance platinum resistance in ovarian cancer cells [40] and cisplatin resistance in lung cancer cells [41]. However, limited studies have explored the relationship between USP1 and Lenvatinib resistance in HCC. In this study, building on prior evidence linking USP1 to lenvatinib response, we define a distinct mitosis-centered resistance program mediated by USP1. These results provide a novel potential target and combination therapy strategy to overcome Lenvatinib resistance in HCC.

DeepMAP-Drug analysis, along with loss-of-function and gain-of-function assays, revealed that USP1 dramatically enhances Lenvatinib resistance in HCC cells. To elucidate how USP1 regulates Lenvatinib resistance in HCC, we performed RNA sequencing and an enrichment analysis in TCGA dataset. Cell cycle process and Mitotic pathways including mitotic spindle organization, spindle assembly, and spindle organization, were significantly enriched in USP1-overexpressed cells or USP1-highly expressed samples. Recent research has shown that intracellular mechanical drugs can induce cell-cycle dysregulation and cell death by preventing spindle centering and correct chromosome alignment [42]. Furthermore, pharmacological inhibition of USP7 induced cell cycle arrest at the G2/M phase and chromosome misalignment [43]. Our data suggest a similar modulatory role of Lenvatinib in mitosis in cells. We found that genetic or pharmacological inhibition of USP1 significantly enhanced Lenvatinib-induced cell cycle arrest at the G2/M phase and chromosome misalignment in HCC cells. Additionally, synergistic inhibitory effects of combining USP1 depletion with Lenvatinib were observed in well-established Lenvatinib-resistant Huh7-R cells.

We then investigated the underlying mechanisms by which USP1 induces mitotic arrest and chromosome misalignment. Four proteins were identified as candidates through combining mass spectrometry and UbiBrowser analysis. As demonstrated, regulating proper chromosome alignment and timely sister chromatid segregation are crucial functions of PLK1. Previous studies have shown that PLK1 also played a significant role in regulating drug resistance in cancer cells. The deubiquitination of PLK1 by USP16 promotes PLK1 localization to the kinetochores and proper chromosome alignment on the metaphase plate. Additionally, PLK1 promotes HCC progression by regulating mitosis [44]. Upon activation, the Polo-box domain (PBD) of PLK1 specifically recognizes and binds to its substrates, leading to the release of an auto-inhibitory sequence and facilitating a transition to an active conformation. Following mitotic functions, this active conformation is targeted by the ubiquitin-proteasome system, where K492 within the PBD is ubiquitinated, marking PLK1 for degradation [45]. Molecular validations confirmed that the USP1-PLK1 regulatory axis contributes to Lenvatinib resistance in HCC cells by influencing chromosome alignment during mitosis. We also found that the E3 ligase STUB1, which has been reported to impede Sorafenib resistance in HCC [46], also restored Lenvatinib sensitivity in HCC. We hypothesize that the USP1/STUB1 axis modulates Lenvatinib sensitivity in HCC cells. Interestingly, we also found that USP1 modulated c-Myc expression via PLK1-mediated phosphorylation, and that c-Myc might act as a transcriptional regulator of USP1. These data support that c-Myc transcriptionally activates USP1, and together with PLK1-mediated c-Myc stabilization, this circuitry establishes a positive feedback loop that sustains the USP1–PLK1 axis.

Recently, we have seen the evolution of systemic therapies towards combination treatments [47]. Lenvatinib combined with pembrolizumab has shown promising antitumor activity in unresectable HCC [48]. Combination treatment with PLK1 inhibitors and abiraterone synergistically disrupts mitosis and kills cancer cells [49]. Our previous results indicate that inhibition of USP1 enhanced the inhibitory effect of Lenvatinib on HCC. We further determined that the combination of Lenvatinib and ML323 synergistically affected PLK1 expression and Lenvatinib resistance. Finally, we identified NTUZLB-001 as a small-molecule modulator that disrupts the USP1–PLK1 interface and consequently impairs PLK1 deubiquitination in cells. Importantly, a fluorogenic deubiquitinase assay showed that NTUZLB-001 does not measurably inhibit the intrinsic catalytic activity of recombinant USP1/UAF1, in contrast to active-site inhibitors such as ML323 and KSQ-4279. These data support an interaction-directed mechanism in which NTUZLB-001 uncouples USP1 from its substrate PLK1, promoting PLK1 ubiquitination and proteasomal turnover. Consistently, NTUZLB-001 synergized with lenvatinib to reduce PLK1 protein levels and sensitize HCC cells to lenvatinib. Together, our findings highlight USP1 as a key regulator of PLK1 stability and underscore the therapeutic potential of targeting DUB–substrate interfaces.

## Conclusion

Collectively, our findings demonstrate that USP1 regulates chromosome alignment by deubiquitinating and stabilizing PLK1, thereby promoting Lenvatinib resistance in HCC cells. USP1 modulates c-Myc expression via PLK1-mediated phosphorylation, and the USP1/PLK1/c-Myc axis forms a positive feedback loop (Figure S8). Additionally, combined administration of Lenvatinib with ML323 or the newly established inhibitor NTUZLB-001 synergistically impairs HCC cells, offering potential targets and novel therapeutic strategies.

## Supplementary Information

Below is the link to the electronic supplementary material.

Supplementary Material 1.

Supplementary Material 2.

## Data Availability

Public datasets used in this study are available from TCGA/ICGC/GEO (accession numbers listed in Methods). Raw data generated in this study are available from the corresponding author upon reasonable request.

## Abbreviations

LEN: Lenvatinib
HCC: Hepatocellular Carcinoma
USP1: Ubiquitin- specific peptidase1
PLK1: Polo-like kinase 1
STUB1: STIP1 homology and U-box-containing protein 1
HMGB1: High mobility group box-1
PFA: Paraformaldehyde
GSEA: Gene set enrichment analysis
CCK8: Cell counting kit 8
FBS: Fetal bovine serum
IHC: Immunohistochemistry

## References

[1] Llovet JM, Kelley RK, Villanueva A, Singal AG, Pikarsky E, Roayaie S, et al. Hepatocellular carcinoma. Nat Rev Dis Primers. 2021;7(1):6. 33479224 10.1038/s41572-020-00240-3 PMC13537433

[2] Finn RS, Zhu AX. Evolution of Systemic Therapy for Hepatocellular Carcinoma. Hepatology. 2021;73 Suppl 1:150–7. 32380571 10.1002/hep.31306

[3] Bo W, Chen Y. Lenvatinib resistance mechanism and potential ways to conquer. Front Pharmacol. 2023;14:1153991. 37153782 10.3389/fphar.2023.1153991 PMC10157404

[4] Mevissen TET, Komander D. Mechanisms of deubiquitinase specificity and regulation. Annu Rev Biochem. 2017;86:159–92. 28498721 10.1146/annurev-biochem-061516-044916

[5] Erven I, Abraham E, Hermanns T, Baumann U, Hofmann K. A widely distributed family of eukaryotic and bacterial deubiquitinases related to herpesviral large tegument proteins. Nat Commun. 2022;13(1):7643. 36496440 10.1038/s41467-022-35244-y PMC9741609

[6] Fraile JM, Quesada V, Rodríguez D, Freije JMP, López-Otín C. Deubiquitinases in cancer: new functions and therapeutic options. Oncogene. 2012;31(19):2373–88. 21996736 10.1038/onc.2011.443

[7] Li H, Roy M, Liang L, Cao W, Hu B, Li Y, et al. Deubiquitylase USP12 induces pro-survival autophagy and bortezomib resistance in multiple myeloma by stabilizing HMGB1. Oncogene. 2022;41(9):1298–308. 34997217 10.1038/s41388-021-02167-9

[8] Guan T, Li M, Song Y, Chen J, Tang J, Zhang C, et al. Phosphorylation of USP29 by CDK1 Governs TWIST1 stability and oncogenic functions. Adv Sci (Weinh). 2023;10(11):e2205873. 36782089 10.1002/advs.202205873 PMC10104637

[9] Ni W, Bian S, Zhu M, Song Q, Zhang J, Xiao M, et al. Identification and validation of ubiquitin-specific proteases as a novel prognostic signature for hepatocellular carcinoma. Front Oncol. 2021;11:629327. 33718205 10.3389/fonc.2021.629327 PMC7949004

[10] García-Santisteban I, Peters GJ, Giovannetti E, Rodríguez JA. USP1 deubiquitinase: cellular functions, regulatory mechanisms and emerging potential as target in cancer therapy. Mol Cancer. 2013;12:91. 23937906 10.1186/1476-4598-12-91 PMC3750636

[11] Liao Y, Shao Z, Liu Y, Xia X, Deng Y, Yu C, et al. USP1-dependent RPS16 protein stability drives growth and metastasis of human hepatocellular carcinoma cells. J Exp Clin Cancer Res. 2021;40(1):201. 34154657 10.1186/s13046-021-02008-3 PMC8215741

[12] Liu D, Li Q, Zang Y, Li X, Li Z, Zhang P, et al. USP1 modulates hepatocellular carcinoma progression via the Hippo/TAZ axis. Cell Death Dis. 2023;14(4):264. 37041150 10.1038/s41419-023-05777-1 PMC10090121

[13] Kalous J, Aleshkina D. Multiple roles of PLK1 in mitosis and meiosis. Cells. 2023;12(1):187. 10.3390/cells12010187 PMC9818836 36611980

[14] de Cárcer G, Manning G, Malumbres M. From Plk1 to Plk5: functional evolution of polo-like kinases. Cell Cycle. 2011;10(14):2255–62. 21654194 10.4161/cc.10.14.16494 PMC3230524

[15] Houston J, Ohta M, Gómez-Cavazos JS, Deep A, Corbett KD, Oegema K et al. BUB-1-bound PLK-1 directs CDC-20 kinetochore recruitment to ensure timely embryonic mitoses. Curr Biol. 2023; 33(11):2291–9. 10.1016/j.cub.2023.04.021 PMC10270731 37137308

[16] Shin S-B, Jang H-R, Xu R, Won J-Y, Yim H. Correction: active PLK1-driven metastasis is amplified by TGF-β signaling that forms a positive feedback loop in non-small cell lung cancer. Oncogene. 2020;39(4):951. 31595031 10.1038/s41388-019-1049-2 PMC7608343

[17] Lashen AG, Toss MS, Wootton L, Green AR, Mongan NP, Madhusudan S et al. Characteristics and prognostic significance of polo-like kinase-1 (PLK1) expression in breast cancer. Histopathology. 2023;83(3):414–25. 10.1111/his.14960 37222669

[18] Zhang J, Zhang L, Wang J, Ouyang L, Wang Y. Polo-like Kinase 1 Inhibitors in human cancer therapy: development and therapeutic potential. J Med Chem. 2022;65(15):10133–60. 10.1021/acs.jmedchem.2c00614 35878418

[19] Van den Bossche J, Lardon F, Deschoolmeester V, De Pauw I, Vermorken JB, Specenier P, et al. Spotlight on volasertib: preclinical and clinical evaluation of a promising Plk1 inhibitor. Med Res Rev. 2016;36(4):749–86. 27140825 10.1002/med.21392

[20] Guerrero-Zotano Á, Belli S, Zielinski C, Gil-Gil M, Fernandez-Serra A, Ruiz-Borrego M, et al. CCNE1 and PLK1 Mediate resistance to Palbociclib in HR+/HER2- metastatic breast cancer. Clin Cancer Res. 2023;29(8):1557–68. 36749874 10.1158/1078-0432.CCR-22-2206 PMC10102847

[21] Yu Z, Deng P, Chen Y, Liu S, Chen J, Yang Z, et al. Inhibition of the PLK1-Coupled cell cycle machinery overcomes resistance to oxaliplatin in colorectal cancer. Adv Sci (Weinh). 2021;8(23):e2100759. 34881526 10.1002/advs.202100759 PMC8655181

[22] Cheng X, Liu Y, Wang J, Chen Y, Robertson AG, Zhang X et al. cSurvival: a web resource for biomarker interactions in cancer outcomes and in cell lines. Brief Bioinform. 2022;23(3):bbac090. 10.1093/bib/bbac090 PMC9116376 35368077

[23] Li Y, Xie P, Lu L, Wang J, Diao L, Liu Z, et al. An integrated bioinformatics platform for investigating the human E3 ubiquitin ligase-substrate interaction network. Nat Commun. 2017;8(1):347. 28839186 10.1038/s41467-017-00299-9 PMC5570908

[24] Lindorff-Larsen K, Piana S, Palmo K, Maragakis P, Klepeis JL, Dror RO, et al. Improved side-chain torsion potentials for the Amber ff99SB protein force field. Proteins. 2010;78(8):1950–8. 20408171 10.1002/prot.22711 PMC2970904

[25] Best RB, Hummer G. Optimized molecular dynamics force fields applied to the helix-coil transition of polypeptides. J Phys Chem B. 2009;113(26):9004–15. 19514729 10.1021/jp901540t PMC3115786

[26] Hornak V, Abel R, Okur A, Strockbine B, Roitberg A, Simmerling C. Comparison of multiple Amber force fields and development of improved protein backbone parameters. Proteins. 2006;65(3):712–25. 16981200 10.1002/prot.21123 PMC4805110

[27] Wang J, Wolf RM, Caldwell JW, Kollman PA, Case DA. Development and testing of a general amber force field. J Comput Chem. 2004;25(9):1157–74. 15116359 10.1002/jcc.20035

[28] Hoover WG. Canonical dynamics: equilibrium phase-space distributions. Phys Rev Gen Phys. 1985;31(3):1695–7. 10.1103/physreva.31.1695 9895674

[29] Yun SM, Moulaei T, Lim D, Bang JK, Park JE, Shenoy SR, et al. Structural and functional analyses of minimal phosphopeptides targeting the polo-box domain of polo-like kinase 1. Nat Struct Mol Biol. 2009;16(8):876–82. 19597481 10.1038/nsmb.1628 PMC2721907

[30] Pierce BG, Wiehe K, Hwang H, Kim BH, Vreven T, Weng Z. ZDOCK server: interactive docking prediction of protein-protein complexes and symmetric multimers. Bioinformatics. 2014;30(12):1771–3. 24532726 10.1093/bioinformatics/btu097 PMC4058926

[31] Moal IH, Barradas-Bautista D, Jimenez-Garcia B, Torchala M, van der Velde A, Vreven T, et al. IRaPPA: information retrieval based integration of biophysical models for protein assembly selection. Bioinformatics. 2017;33(12):1806–13. 28200016 10.1093/bioinformatics/btx068 PMC5783285

[32] O’Boyle NM, Banck M, James CA, Morley C, Vandermeersch T, Hutchison GR. Open Babel: An open chemical toolbox. J Cheminform. 2011;3:33. 21982300 10.1186/1758-2946-3-33 PMC3198950

[33] Yu Y, Cai C, Wang J, Bo Z, Zhu Z, Zheng H. Uni-Dock: GPU-Accelerated docking enables ultralarge virtual screening. J Chem Theory Comput. 2023;19(11):3336–45. 37125970 10.1021/acs.jctc.2c01145

[34] Yang M, Bo Z, Xu T, Xu B, Wang D, Zheng H. Uni-GBSA: an open-source and web-based automatic workflow to perform MM/GB(PB)SA calculations for virtual screening. Brief Bioinform. 2023;24(4):bbad218. 10.1093/bib/bbad218 37328705

[35] Valdes-Tresanco MS, Valdes-Tresanco ME, Valiente PA, Moreno E. gmx_MMPBSA: a new tool to perform end-state free energy calculations with GROMACS. J Chem Theory Comput. 2021;17(10):6281–91. 34586825 10.1021/acs.jctc.1c00645

[36] Bian S, Ni W, Zhou L, Tong Y, Dai C, Zhao X, et al. Ubiquitin-specific protease 1 facilitates hepatocellular carcinoma progression by modulating mitochondrial fission and metabolic reprogramming via cyclin-dependent kinase 5 stabilization. Cell Death Differ. 2024;31(9):1202–18. 39009653 10.1038/s41418-024-01342-1 PMC11369097

[37] Zheng J, Tan Y, Liu X, Zhang C, Su K, Jiang Y, et al. NAT10 regulates mitotic cell fate by acetylating Eg5 to control bipolar spindle assembly and chromosome segregation. Cell Death Differ. 2022;29(4):846–60. 35210604 10.1038/s41418-021-00899-5 PMC8989979

[38] Rennie ML, Arkinson C, Chaugule VK, Toth R, Walden H. Structural basis of FANCD2 deubiquitination by USP1-UAF1. Nat Struct Mol Biol. 2021;28(4):356–64. 33795880 10.1038/s41594-021-00576-8

[39] Liao Y, Shao Z, Liu Y, Xia X, Deng Y, Yu C, et al. USP1-dependent RPS16 protein stability drives growth and metastasis of human hepatocellular carcinoma cells. J experimental Clin cancer research: CR. 2021;40(1):201. 10.1186/s13046-021-02008-3 PMC8215741 34154657

[40] Sonego M, Pellarin I, Costa A, Vinciguerra GLR, Coan M, Kraut A, et al. USP1 links platinum resistance to cancer cell dissemination by regulating snail stability. Sci Adv. 2019;5(5):eaav3235. 31086816 10.1126/sciadv.aav3235 PMC6506239

[41] Tyagi A, Kaushal K, Chandrasekaran AP, Sarodaya N, Das S, Park CH, et al. CRISPR/Cas9-based genome-wide screening for deubiquitinase subfamily identifies USP1 regulating MAST1-driven cisplatin-resistance in cancer cells. Theranostics. 2022;12(13):5949–70. 35966591 10.7150/thno.72826 PMC9373805

[42] Arjona MI, Duch M, Hernández-Pinto A, Vázquez P, Agusil JP, Gómez-Martínez R, et al. Intracellular mechanical drugs induce cell-cycle altering and cell death. Adv Mater. 2022;34(17):e2109581. 35174908 10.1002/adma.202109581

[43] Peng Y, Liu Y, Gao Y, Yuan B, Qi X, Fu Y, et al. USP7 is a novel Deubiquitinase sustaining PLK1 protein stability and regulating chromosome alignment in mitosis. J experimental Clin cancer research: CR. 2019;38(1):468. 10.1186/s13046-019-1457-8 PMC6858727 31730000

[44] Liu J, Zhang C. The equilibrium of ubiquitination and deubiquitination at PLK1 regulates sister chromatid separation. Cell Mol Life Sci. 2017;74(12):2127–34. 28188342 10.1007/s00018-017-2457-5 PMC11107562

[45] Beck J, Maerki S, Posch M, Metzger T, Persaud A, Scheel H, et al. Ubiquitylation-dependent localization of PLK1 in mitosis. Nat Cell Biol. 2013;15(4):430–9. 23455478 10.1038/ncb2695 PMC7116173

[46] Liao Y, Liu Y, Yu C, Lei Q, Cheng J, Kong W, et al. HSP90β Impedes STUB1-Induced ubiquitination of YTHDF2 to drive sorafenib resistance in hepatocellular carcinoma. Adv Sci (Weinh). 2023;10(27):e2302025. 37515378 10.1002/advs.202302025 PMC10520652

[47] Yang C, Zhang H, Zhang L, Zhu AX, Bernards R, Qin W, et al. Evolving therapeutic landscape of advanced hepatocellular carcinoma. Nat Reviews Gastroenterol Hepatol. 2023;20(4):203–22. 10.1038/s41575-022-00704-9 36369487

[48] Makker V, Colombo N, Herráez AC, Monk BJ, Mackay H, Santin AD, et al. Lenvatinib Plus Pembrolizumab in previously treated advanced endometrial cancer: updated efficacy and safety from the randomized phase III study 309/KEYNOTE-775. J Clin Oncol. 2023;41(16):2904–10. 37058687 10.1200/JCO.22.02152 PMC10414727

[49] Patterson JC, Varkaris A, Croucher PJP, Ridinger M, Dalrymple S, Nouri M, et al. Plk1 inhibitors and abiraterone synergistically disrupt mitosis and kill cancer cells of disparate origin independently of androgen receptor signaling. Cancer Res. 2023;83(2):219–38. 36413141 10.1158/0008-5472.CAN-22-1533 PMC9852064

