# Supplementary material for: USP1 driven mitotic dysregulation and PLK1 stabilization confer Lenvatinib resistance in hepatocellular carcinoma
Source: J Exp Clin Cancer Res. 2026 Mar 13;45:87. doi: 10.1186/s13046-026-03683-w (PMC13045141; doi:10.1186/s13046-026-03683-w)
Supplement: Supplementary file 1 — Supplementary Material 1. [file 13046_2026_3683_MOESM1_ESM.pdf]

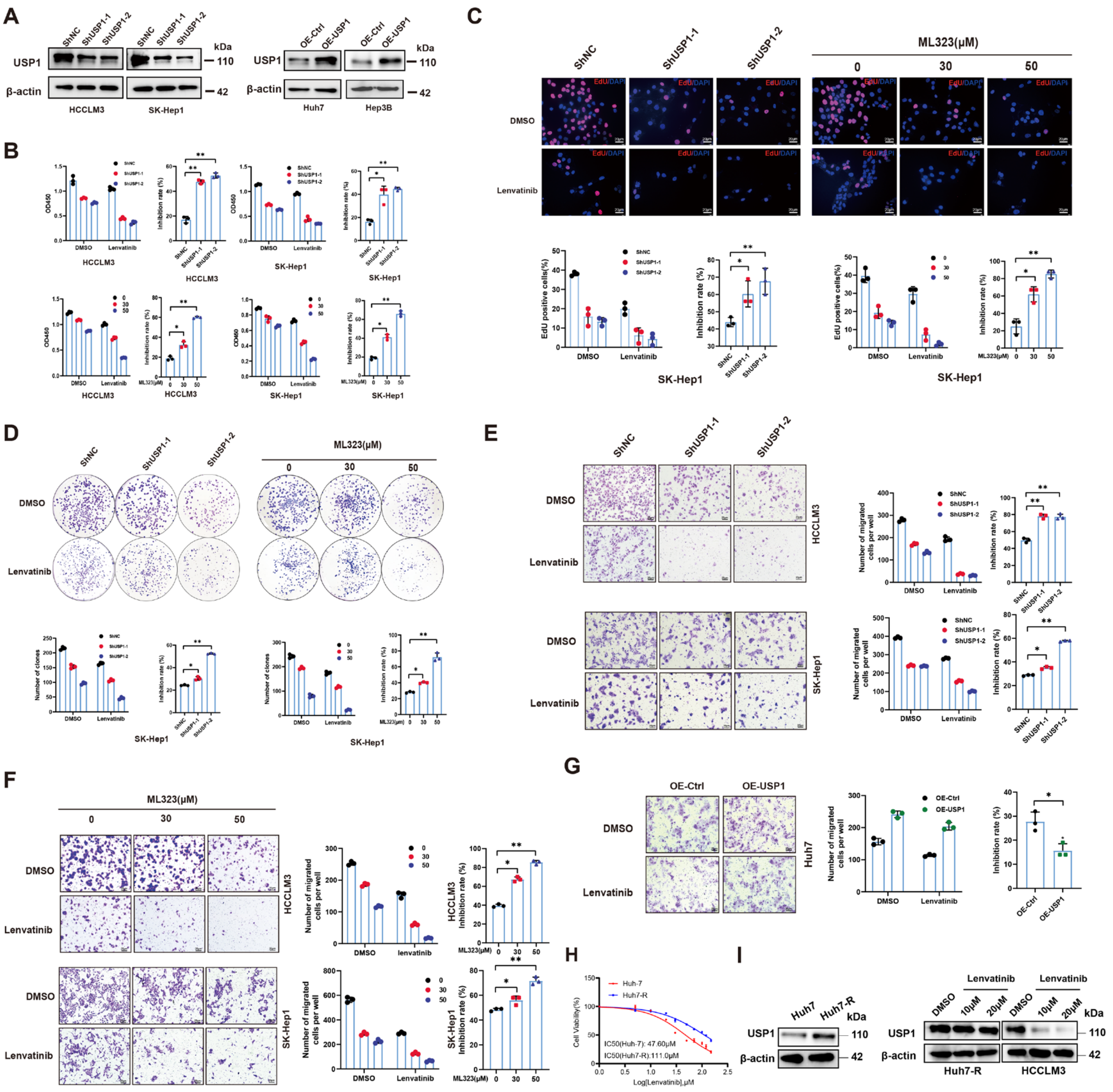

Fig. S1. Synergistic inhibitory effects of genetic and pharmacological depletion of USP1 on HCC cell proliferation and migration. (A) Transfection efficiency of USP1 in SK-Hep1, HCCLM3, Huh7, and Hep3B cells. **(B-D)** Synergistic inhibitory effects of USP1 genetic and pharmacological depletion on SK-Hep1 cell proliferation in combination with Lenvatinib, assessed by CCK-8, EdU staining, and colony formation assays. **(E&F)** Synergistic effects of USP1 depletion on the migratory capacity of SK-Hep1 cells in the presence of Lenvatinib, measured by transwell assay. **(G)** Effects of USP1 overexpression on the migratory capacity of Huh7 cells in the presence of Lenvatinib, measured by transwell assay. (H) Dose–response curves of lenvatinib in parental Huh7 and lenvatinib-resistant Huh7-R cells. (I) USP1 protein levels in parental Huh7 and Huh7-R cells, and changes in USP1 expression upon lenvatinib treatment in Huh7-R and HCCLM3 cells, as determined by western blotting. **, P<0.01; *, P<0.05.

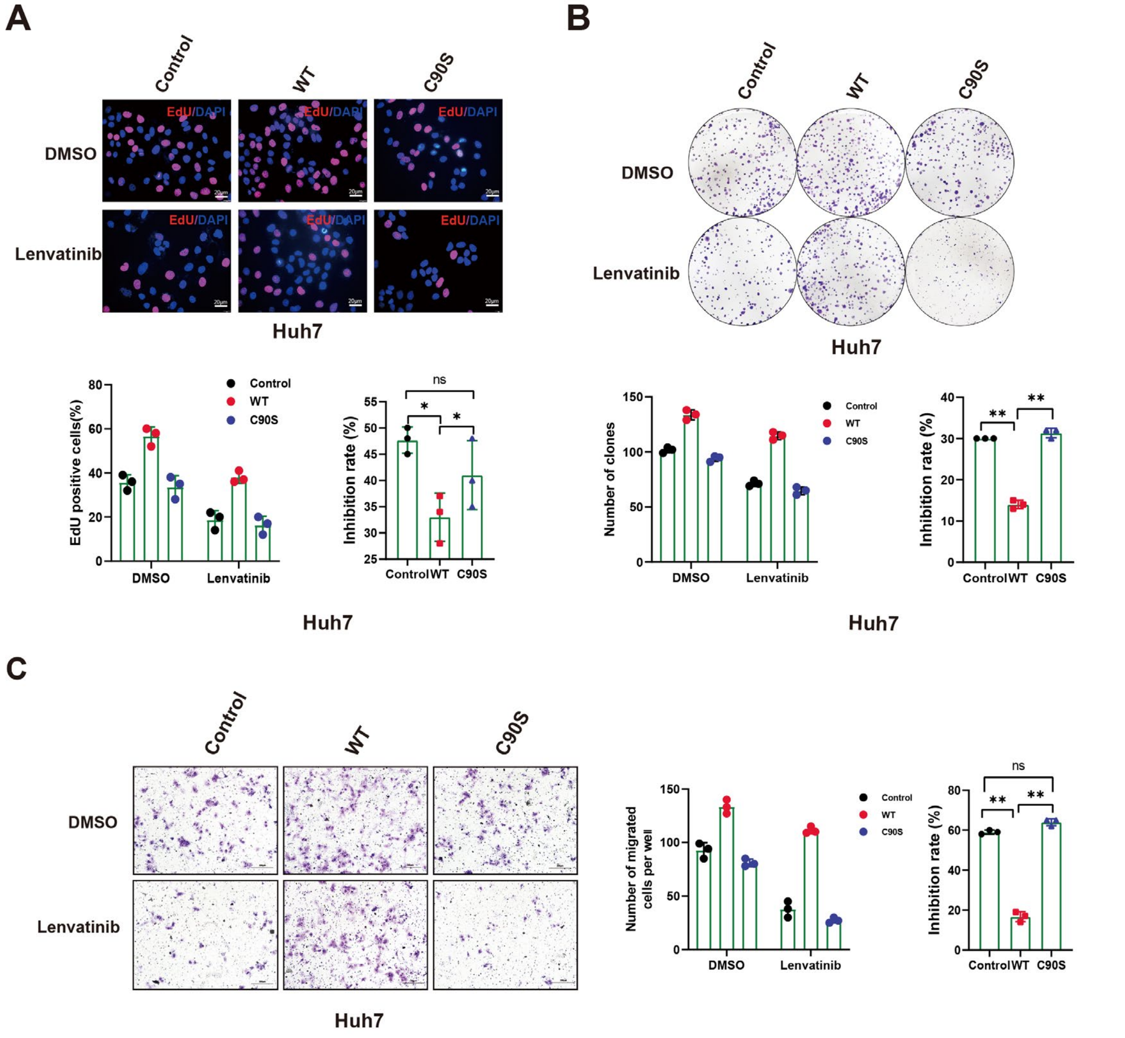

Fig. S2. Catalytically inactive mutation of USP1 fails to enhance Lenvatinib sensitivity in HCC. (A&B) Inhibition rate of Lenvatinib (10 μM) on proliferation in Huh7 cells transfected with Control, WT, and C90S USP1 plasmids, assessed by EdU staining and colony formation assays. **(C)** Inhibition rate of Lenvatinib (10 μM) on migration in Huh7 cells transfected with Control, WT, and C90S USP1 plasmids, evaluated by EdU staining and colony formation assays. **, $P<0.01$; *, $P<0.05$.

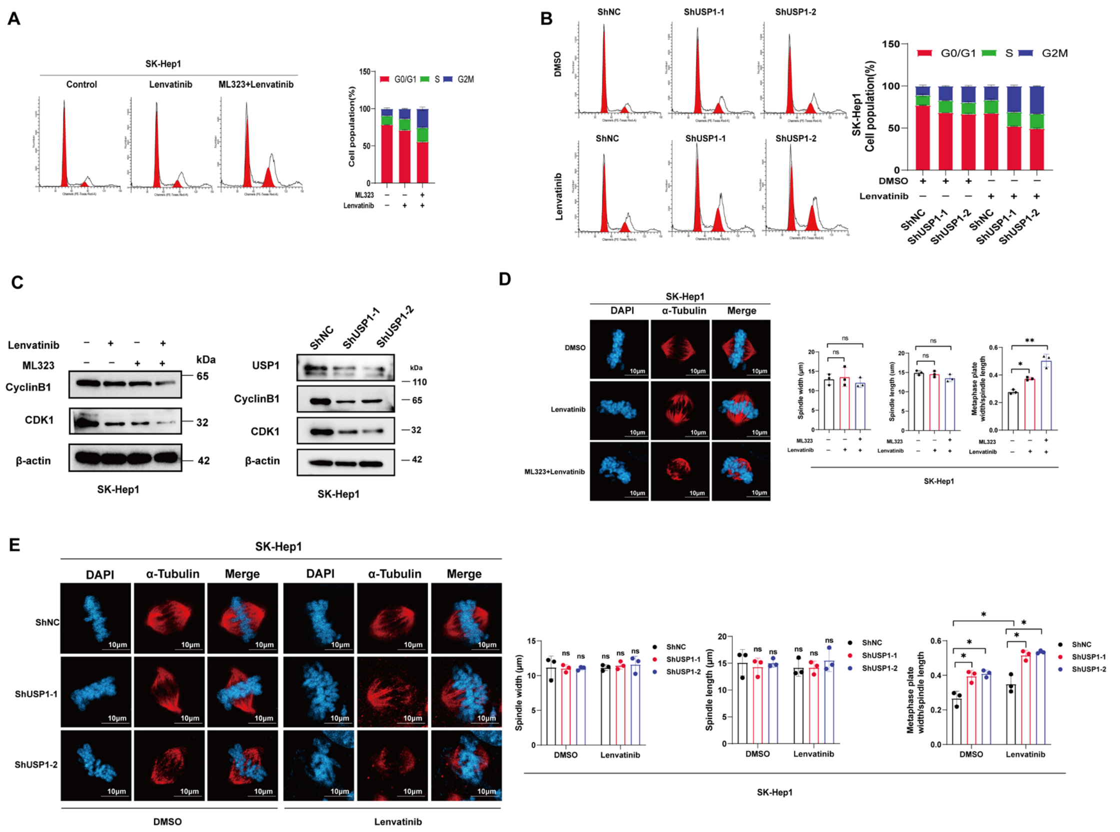

Fig. S3. Genetic or pharmacological depletion of USP1 induces mitotic arrest and chromosome misalignment. (A&B) Flow cytometry analysis showing cell cycle distribution in different treatment groups of SK-Hep1 cells. **(C)** Immunoblotting analysis of Cyclin B1 and CDK1 expression in SK-Hep1 cells under different treatments. **(D&E)** Chromosome misalignment in SK-Hep1 cells, pre-synchronized to metaphase, observed under different treatments. α-Tubulin is shown in red, and DAPI in blue. **, P<0.01; *, P<0.05.

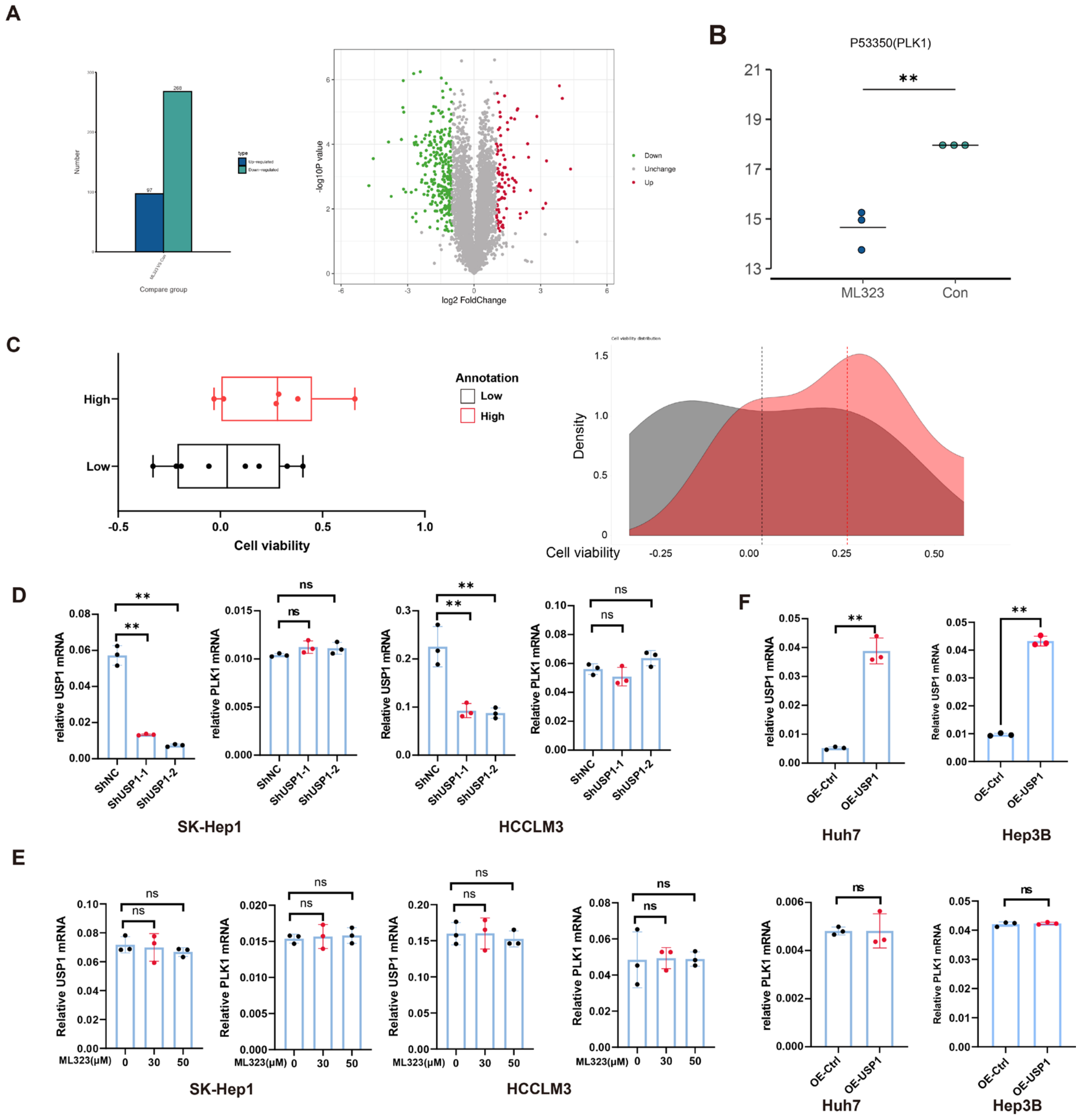

Fig. S4. USP1 has minimal effects on PLK1 at the mRNA level, which correlates with Lenvatinib sensitivity. **(A)** Bar chart and volcano plot showing differentially expressed proteins between control and ML323-treated cells. **(B)** Mass spectrometry-based identification of PLK1 expression in control and ML323-treated cells. **(C)** Lenvatinib sensitivity in 18 hepatoma cell lines with high or low PLK1 expression, as identified in the DeepMap-Drug database. **(D-F)** mRNA expression levels of USP1 and PLK1 in SK-Hep1 and HCCLM3 cells transfected with ShNC or ShUSP1/2 plasmids, and in Huh7 and Hep3B cells treated with OE-Ctrl or OE-USP1 plasmids. **, $P < 0.01$.

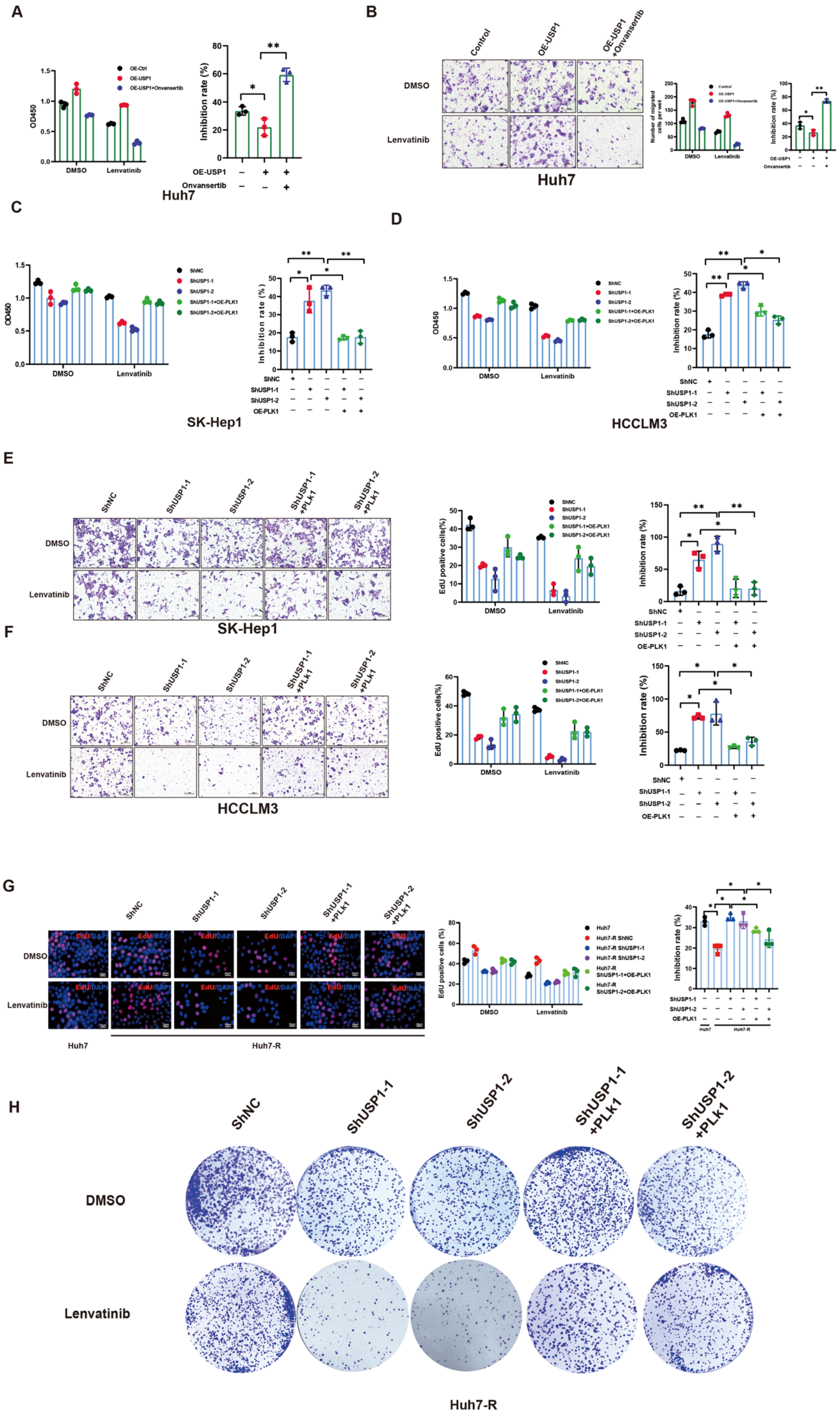

Fig. S5. PLK1 mediates USP1-induced Lenvatinib resistance in HCC cells. (A&B) CCK-8 and transwell assays showing the inhibition rate of Lenvatinib in Huh7 cells treated with Onvansertib (5 μM) following transfection with OE-USP1 plasmids. **(C-G)** The inhibition rate of Lenvatinib (10 μM) in SK-Hep1 and HCCLM3 cells co-transfected with ShUSP1 and OE-PLK1 plasmids, assessed by CCK-8, EdU staining, and transwell assays, respectively. **(H)** Colony formation assays conducted on Lenvatinib-resistant Huh7-R cells with indicated treatments. **, P < 0.01; *, P < 0.05.

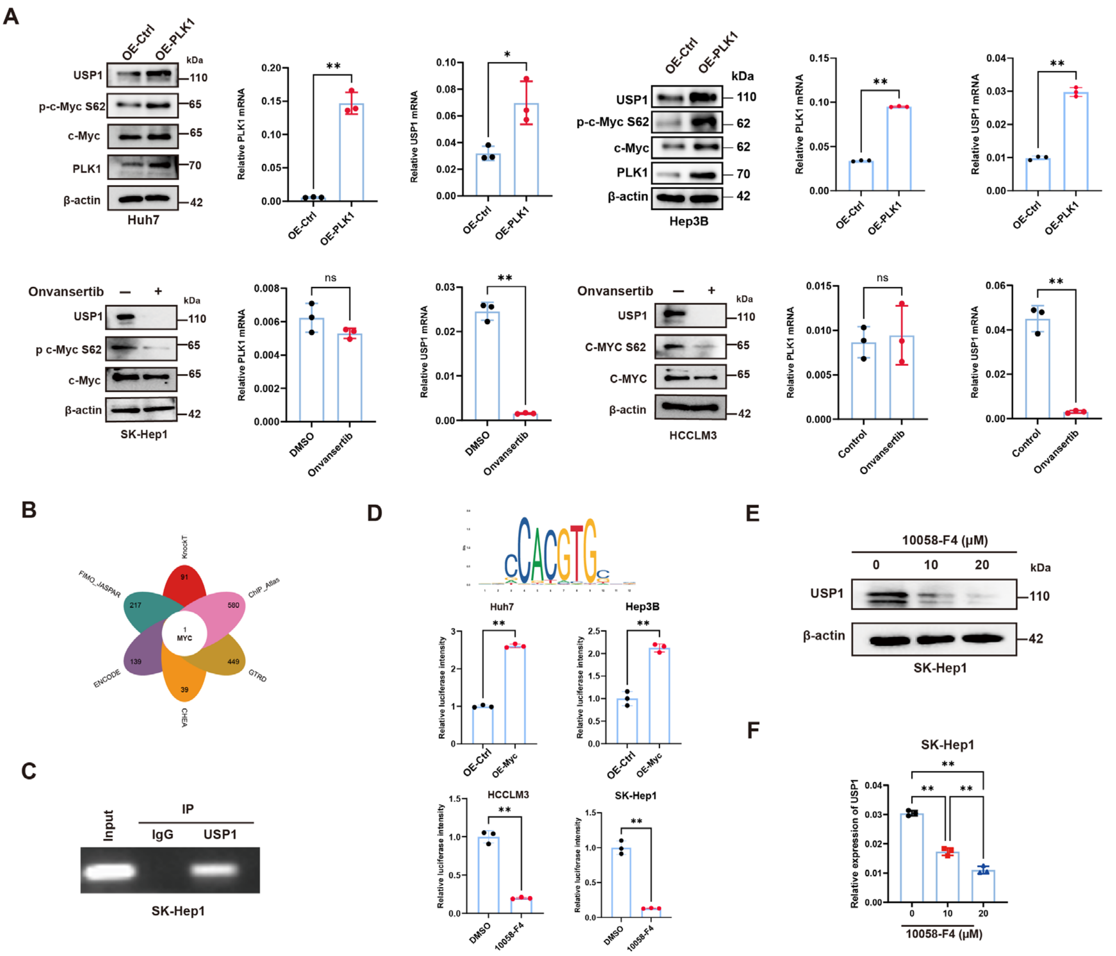

Fig. S6. USP1/PLK1/c-Myc forms a positive feedback axis. (A) The transcriptional and protein levels of USP1 and PLK1 in HCC cells with PLK1 overexpression or Onvansertib treatment were detected by western blotting and RT-qPCR. **(B)** Six transcription factor databases were combed to predict potential transcription factors of USP1. **(C)** ChIP assay was performed to detect the binding of c-Myc to the USP1 promoter. **(D)** Putative c-Myc–binding motif within the USP1 promoter and the effects of c-Myc overexpression or 10058-F4 treatment on USP1 promoter activity were evaluated using a dual-luciferase reporter assay. **(E&F)** The inhibitory effects of 10058-F4, a c-Myc inhibitor, on USP1 at both protein and mRNA levels. **, P < 0.01; *, P < 0.05.

DAD: 220

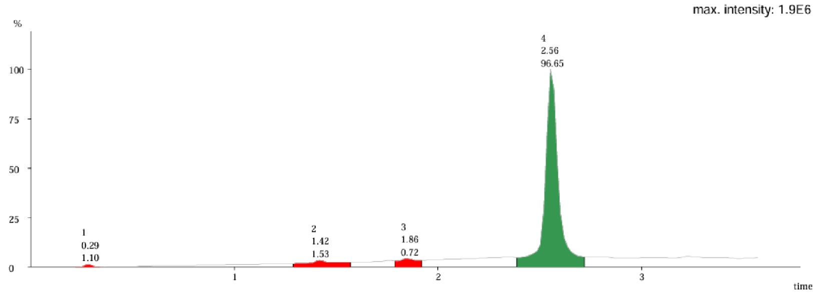

Peak_ID	Peak	Area	Area%	Height	Time	Mass Found
1	0.22 0.34	1.E3	1.1	3.E4	0.29	
2	1.29 1.57	2.E3	1.53	3.E4	1.42	
3	1.79 1.92	9.E2	0.72	2.E4	1.86	
4	2.39 2.72	1.E5	96.65	2.E6	2.56	566.14

MS ES+ :567.14

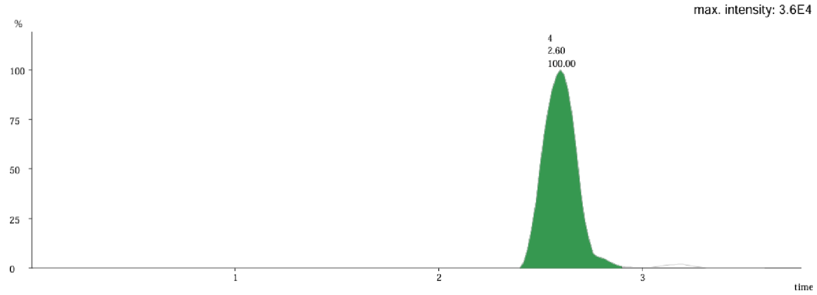

Peak_ID	Peak	Area	Area%	Height	Time	Mass Found
4	2.40 2.90	7.E3	100	4.E4	2.60	566.14

MS: ES+

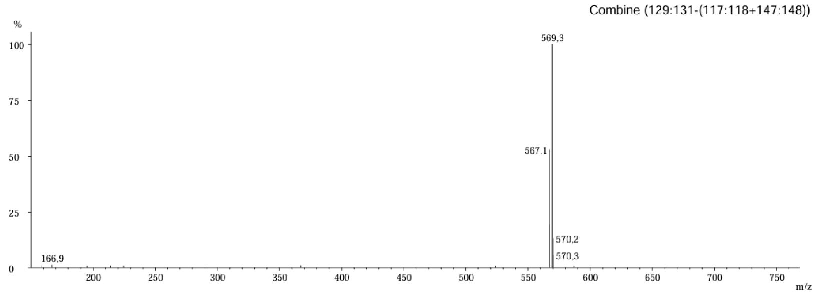

Peak_ID	Compound	Time	Mass found
4	Found	2.60	566.1400

Fig. S7. LC-MS spectra of NTUZLB-001. The characterization of NTUZLB-001.

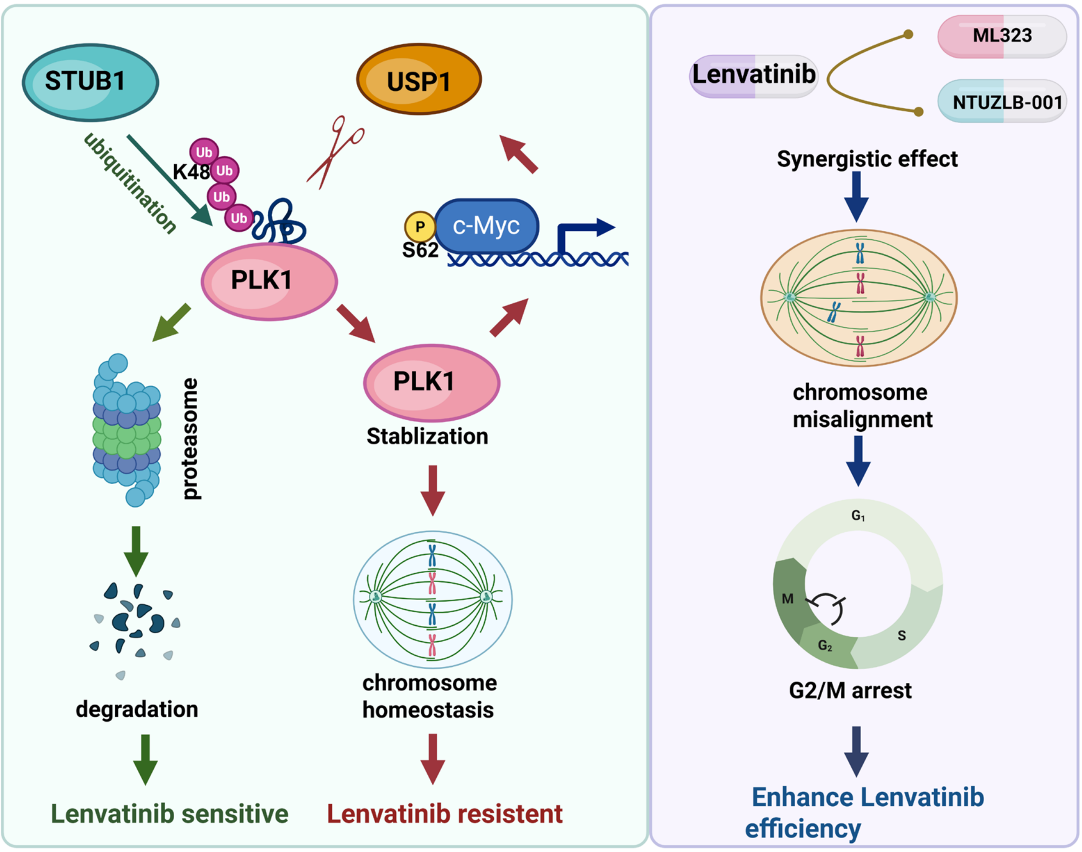

Fig. S8. The mechanism graph of this study.

Table S1. The sequences of gene-specific primers used for RT-qPCR assays

Gene		Sequence
β-Actin	Forward Primer	AGAACTGGCCCTTCTTGGAGG
	Reverse Primer	GTTTTTATGTTCCTCTATGGG
USP1	Forward Primer	CCAATGAGAGCGGAAGGAGG
	Reverse Primer	CACCAATTATATCTAGACCAAAGCC
PLK1	Forward Primer	CGAGTTCTTTACTTCTGGCT
	Reverse Primer	TATTGAGGACTGTGAGGGGC
STUB1	Forward Primer	TCAAGGAGCAGGGCAATCGT
	Reverse Primer	CAGCGGGTTCCGGGTGAT
