# Supplementary material for: USP1 driven mitotic dysregulation and PLK1 stabilization confer Lenvatinib resistance in hepatocellular carcinoma
Source: J Exp Clin Cancer Res. 2026 Mar 13;45:87. doi: 10.1186/s13046-026-03683-w (PMC13045141; doi:10.1186/s13046-026-03683-w)

Figure 1

C

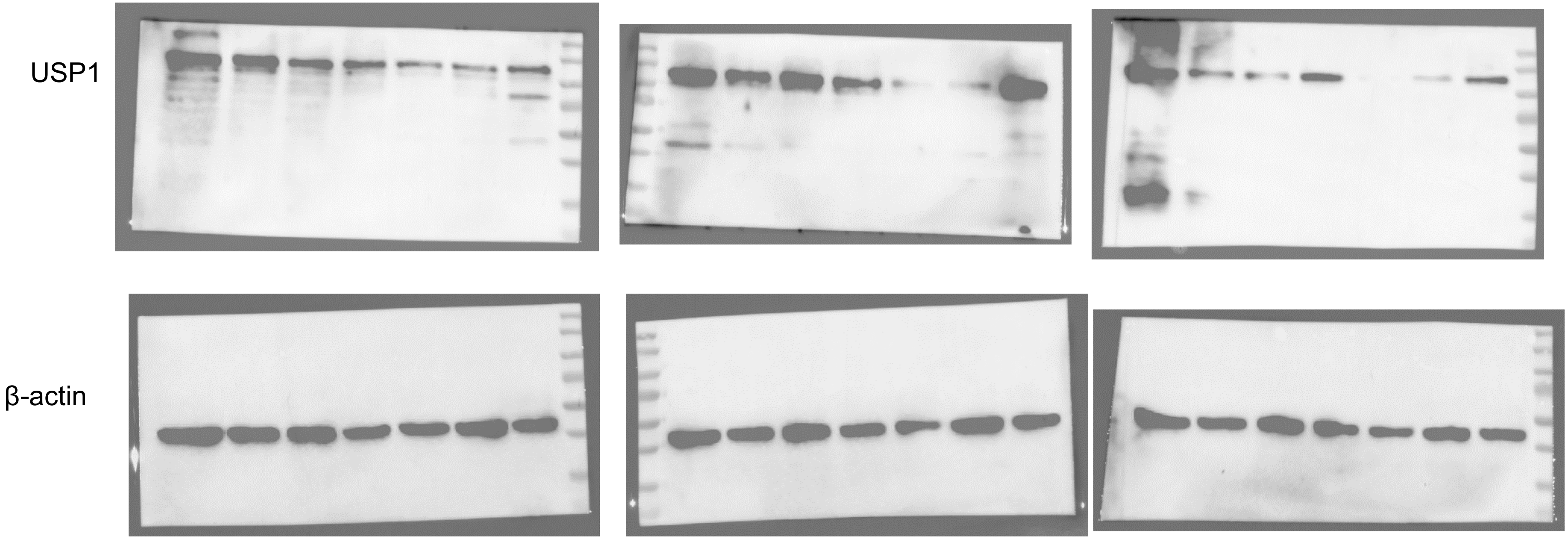

Figure S1A

USP1

HCCLM3

β-actin

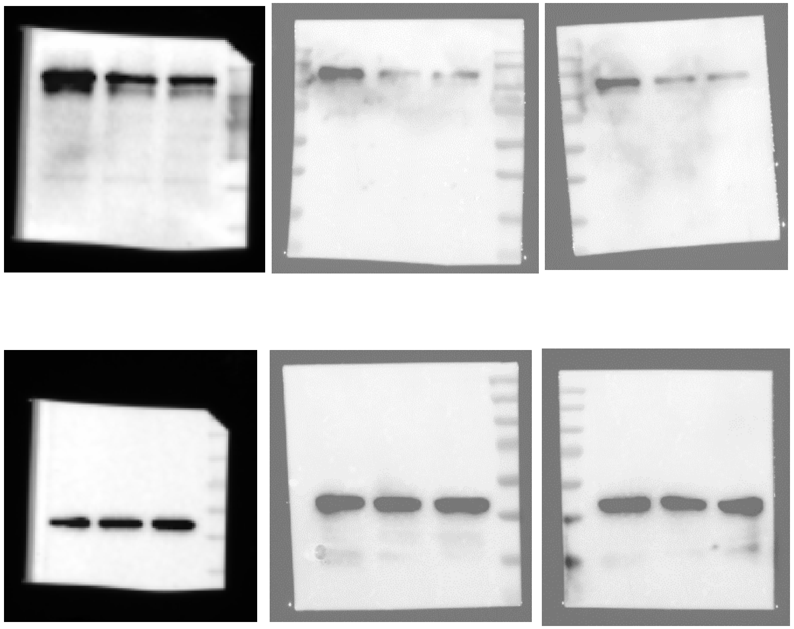

Figure S1A

SK-Hep1

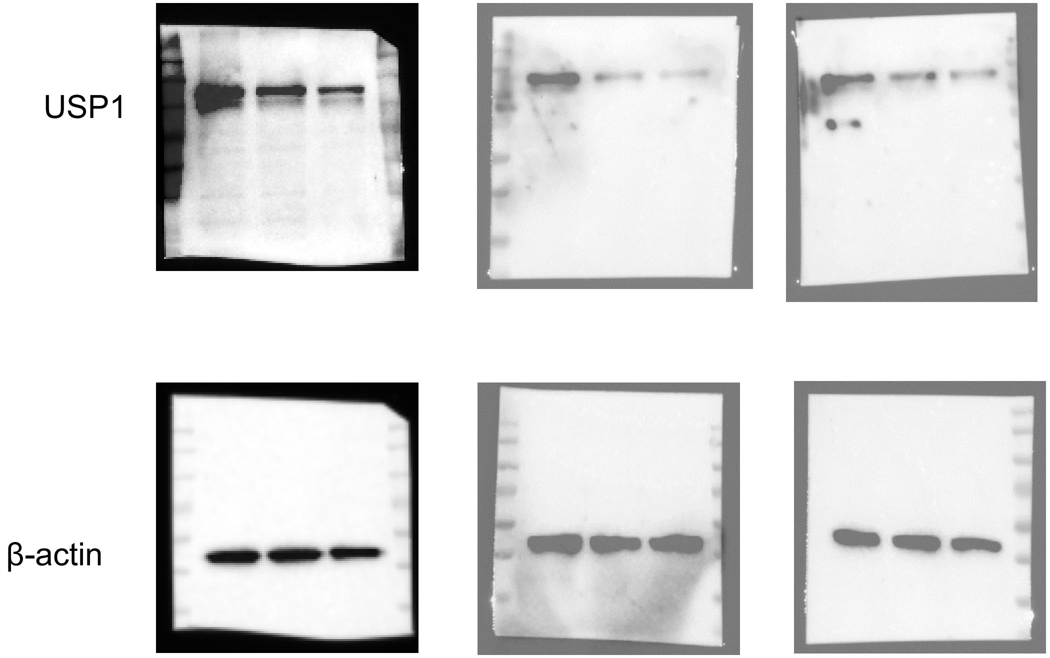

Figure S1A

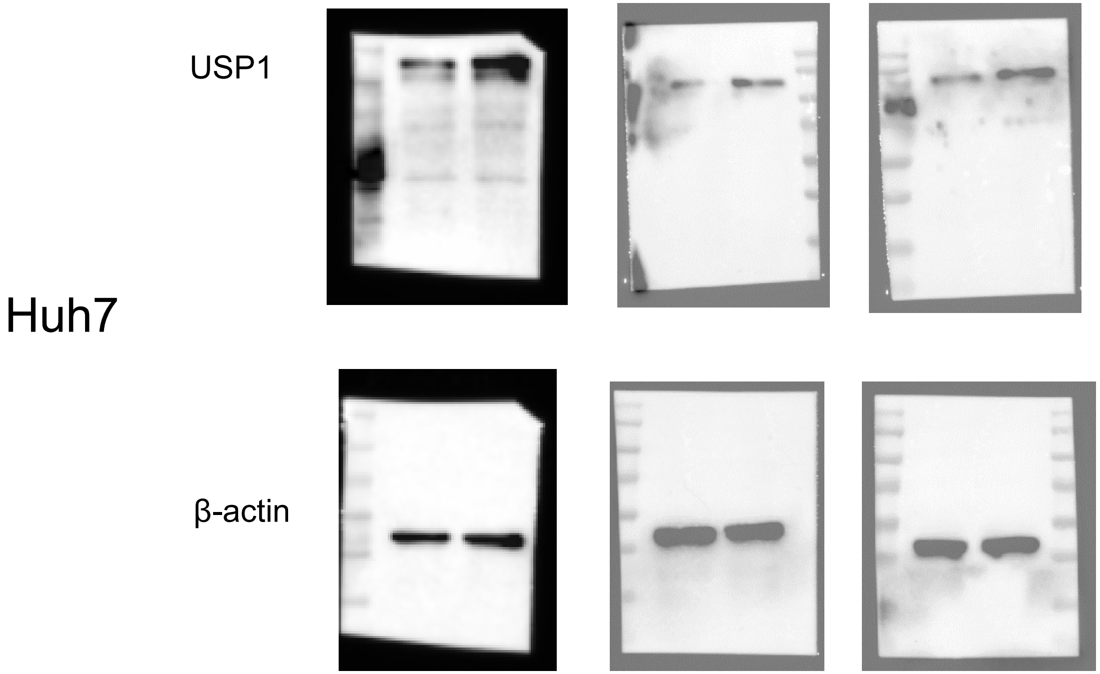

Figure S1A

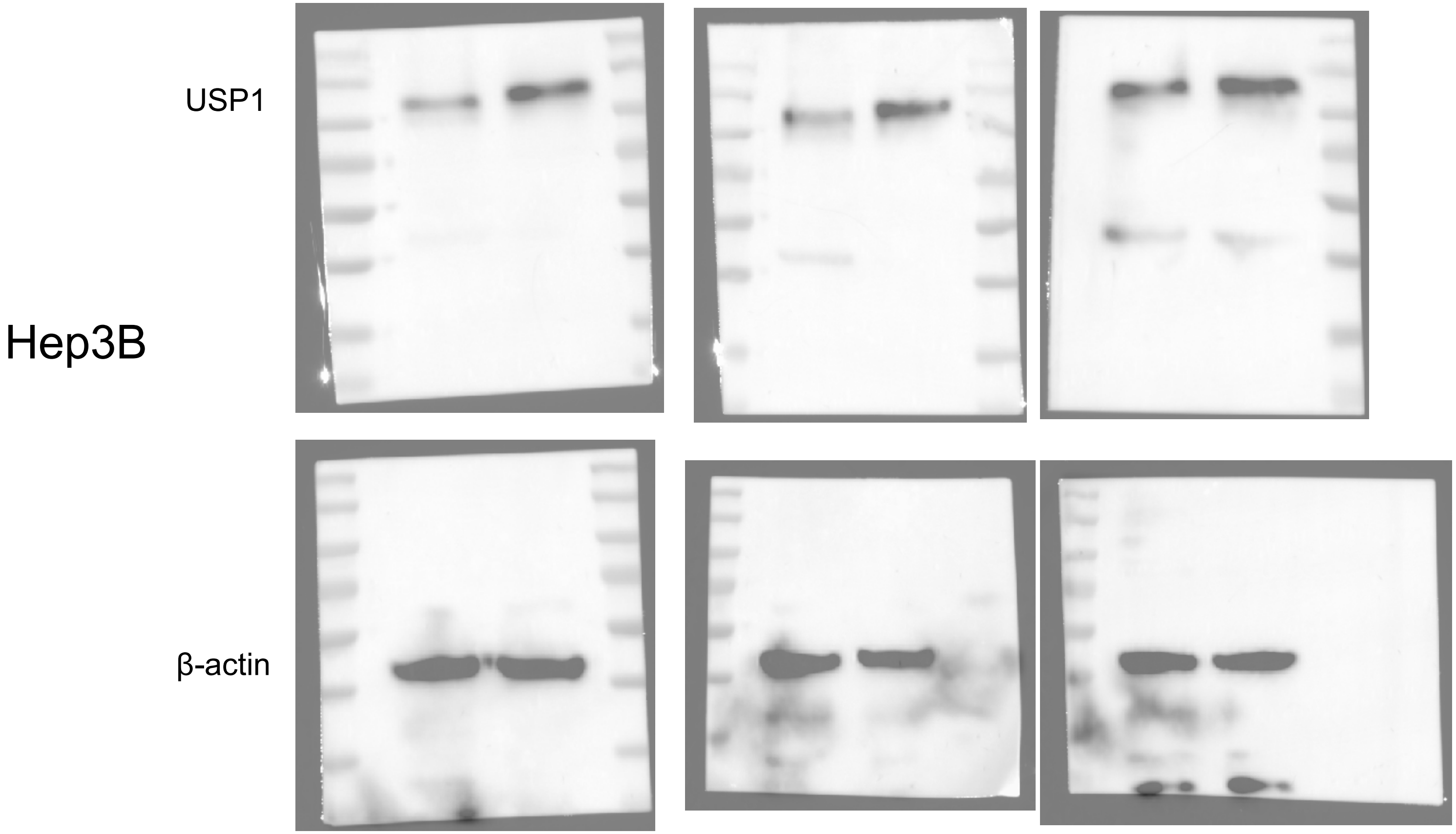

Figure S1I

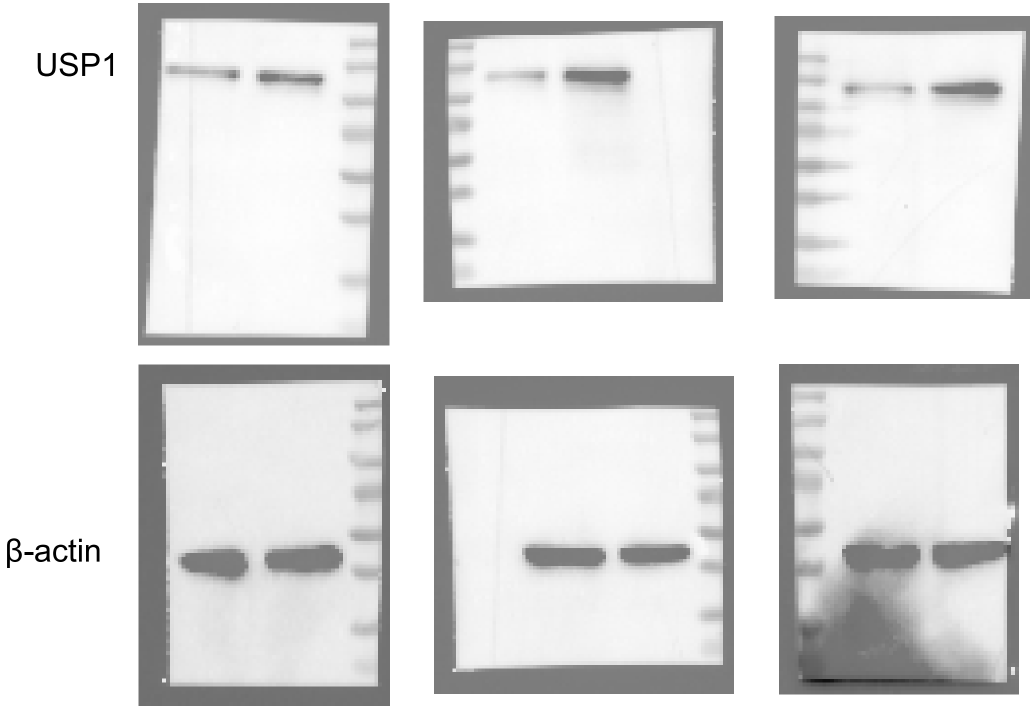

Figure S1I

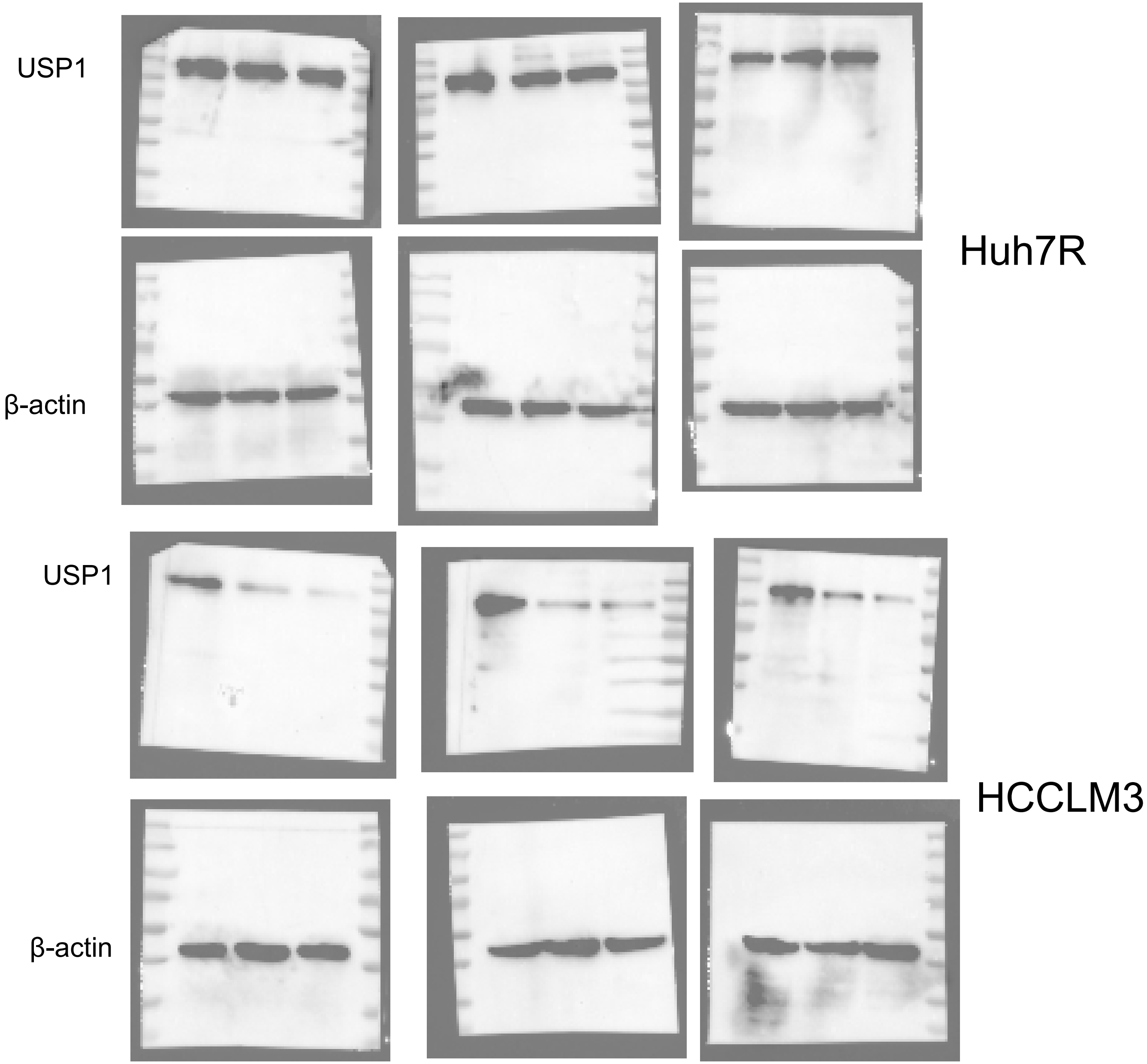

Figure 2J

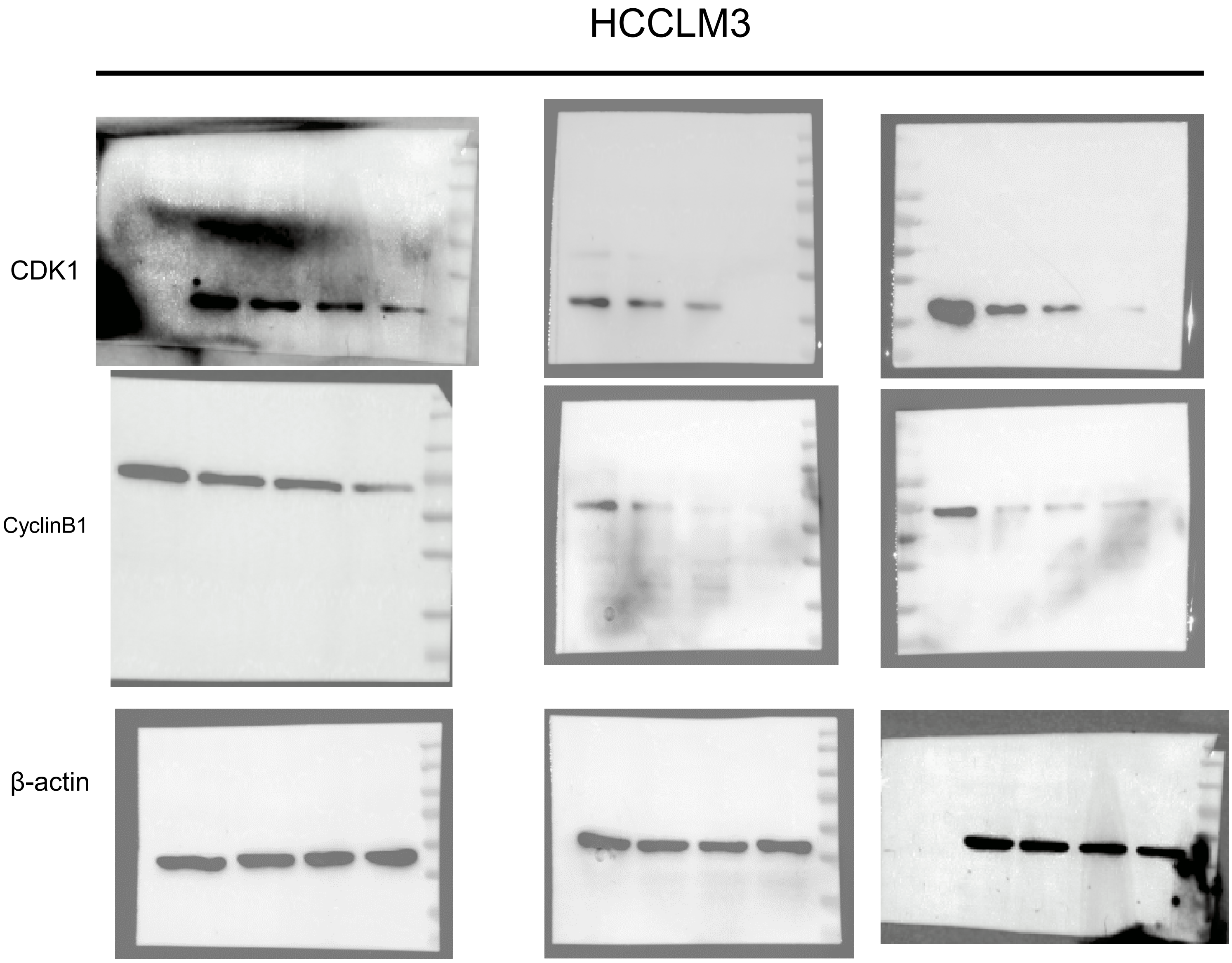

Figure 2J

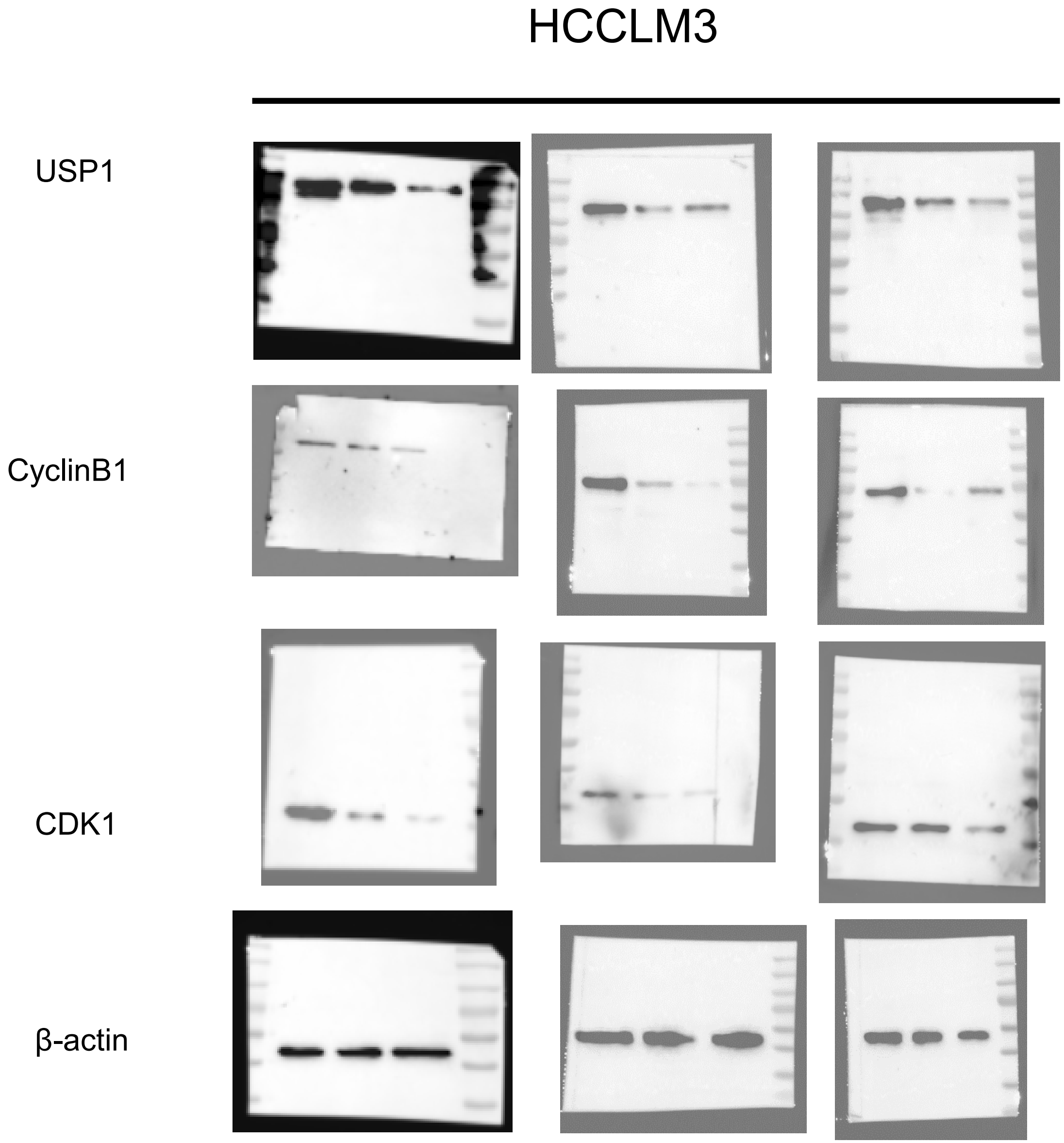

Figure 2J

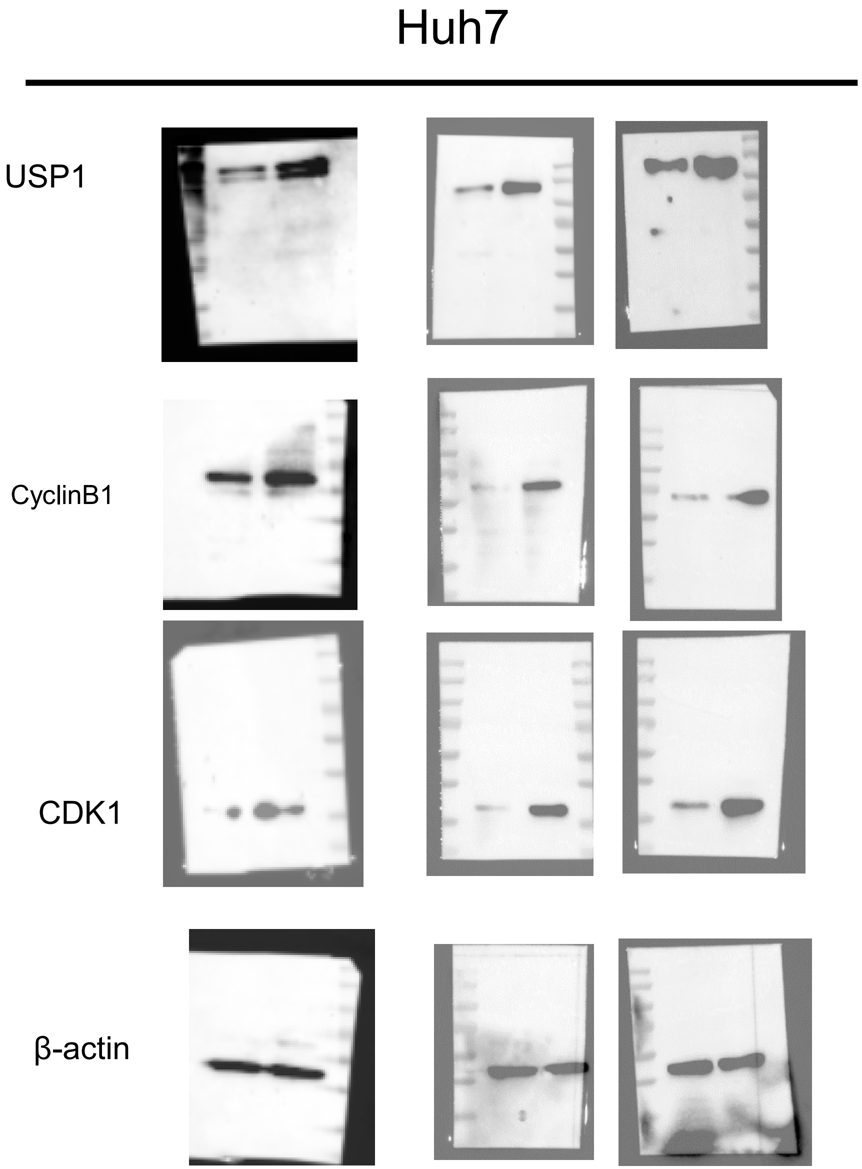

Figure 2J

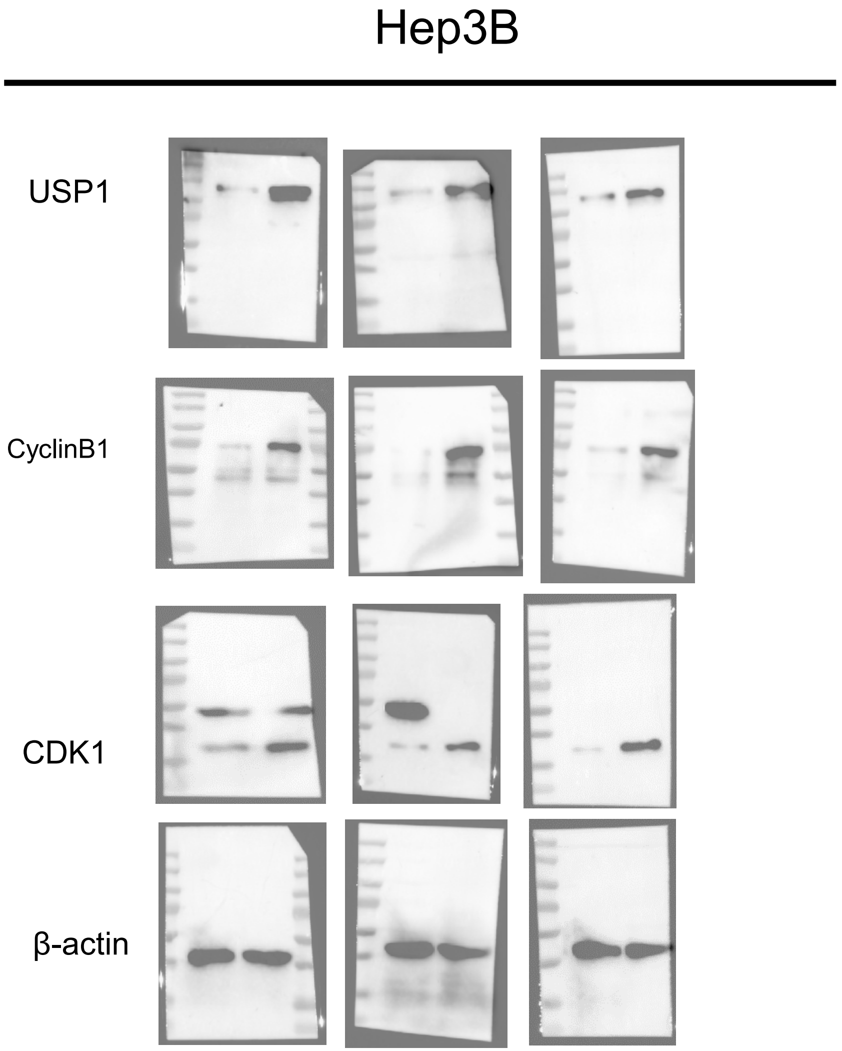

Figure S3C

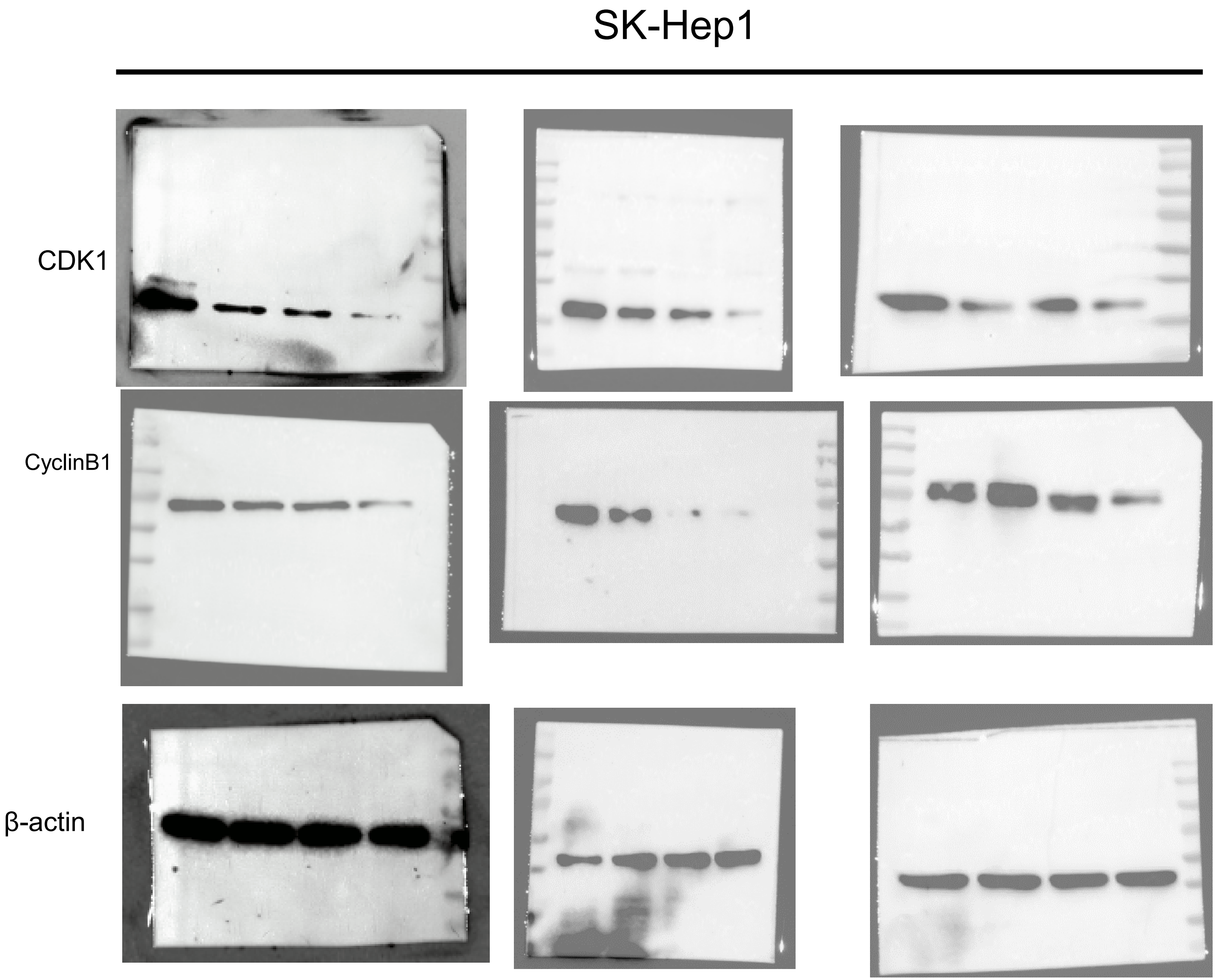

Figure S3C

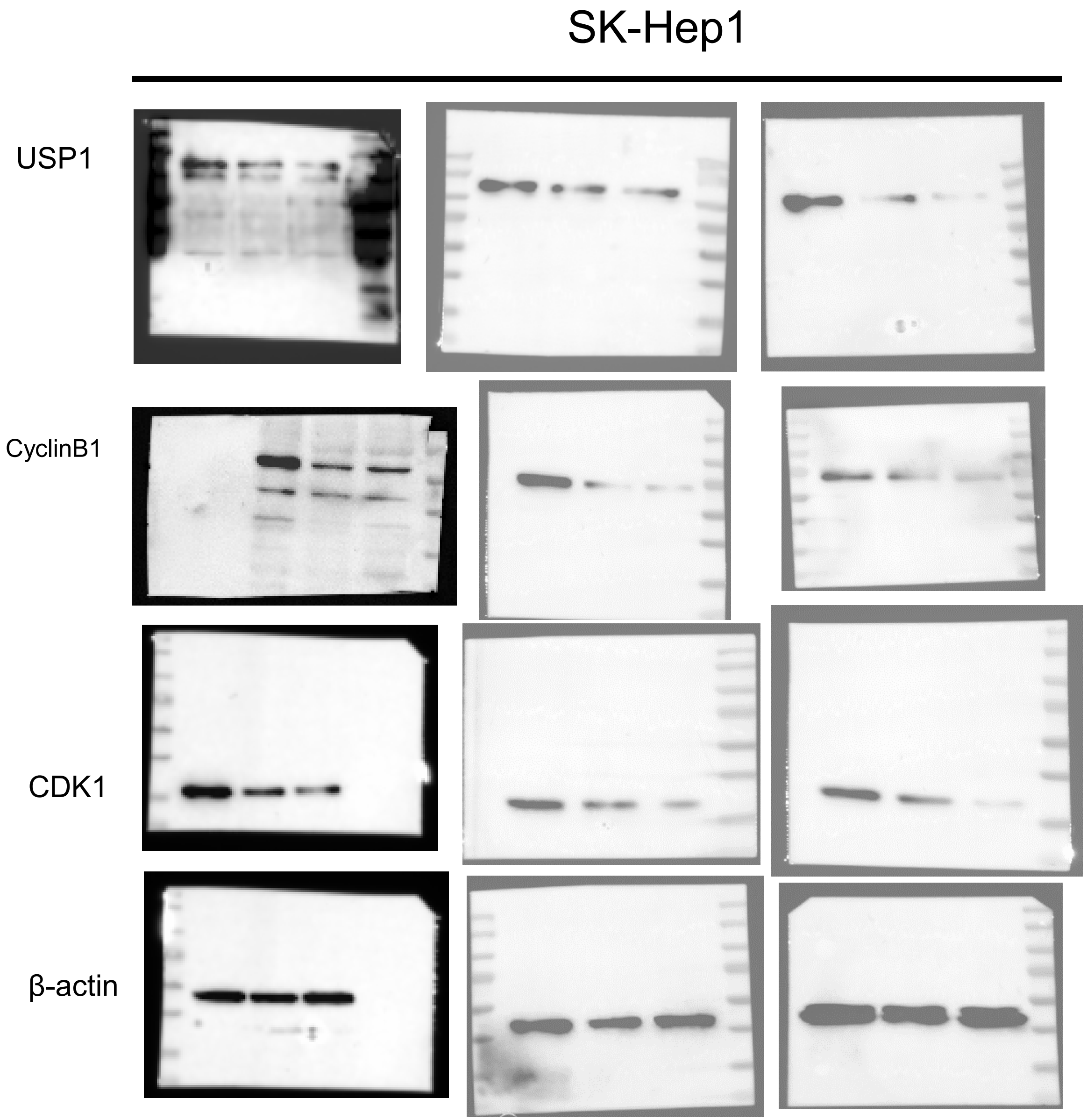

Figure 3 C

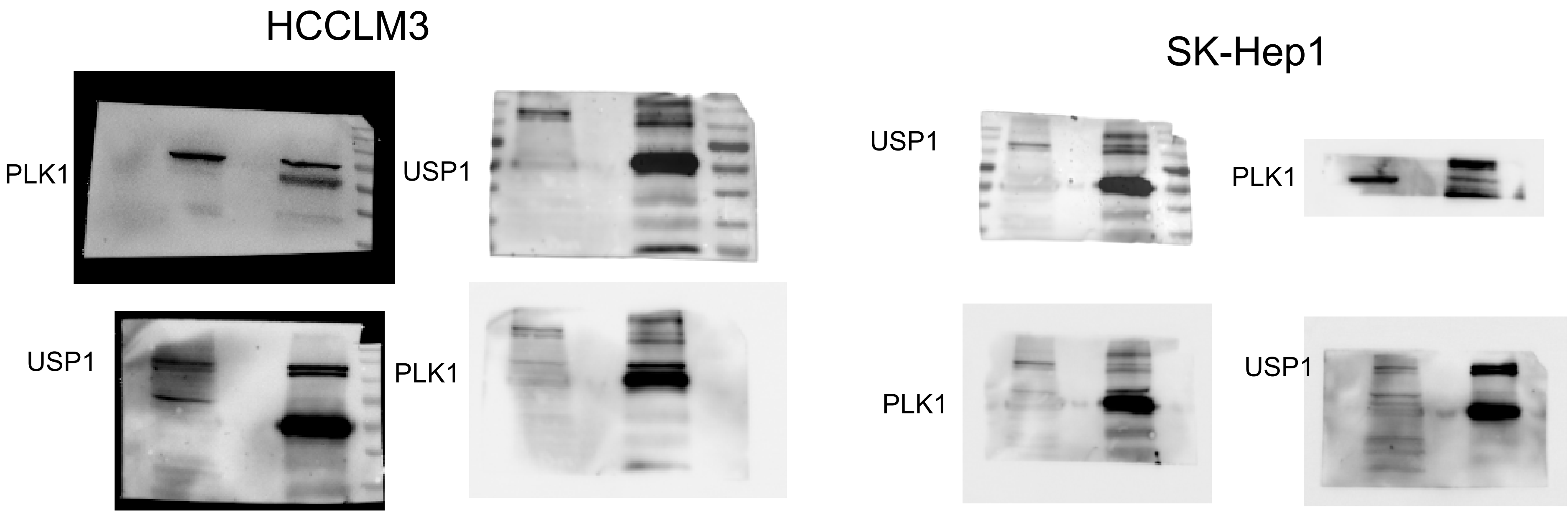

Figure 3 D

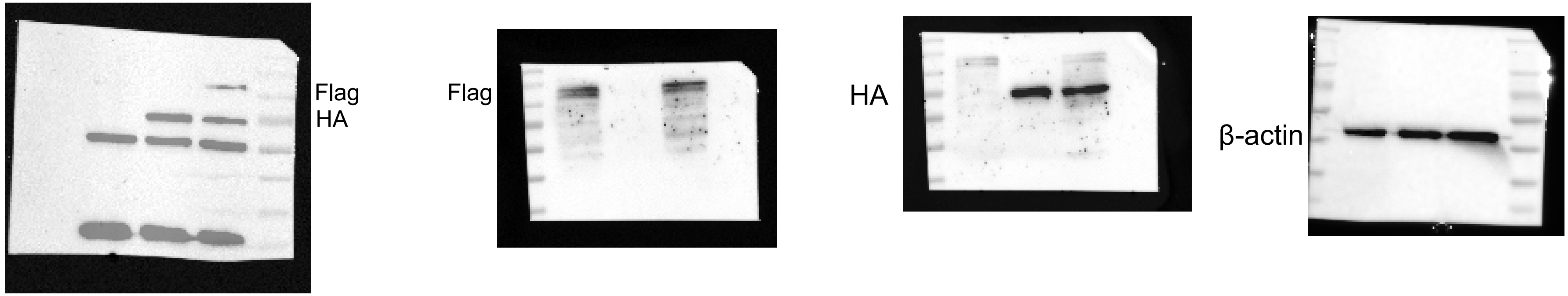

Figure 3 F

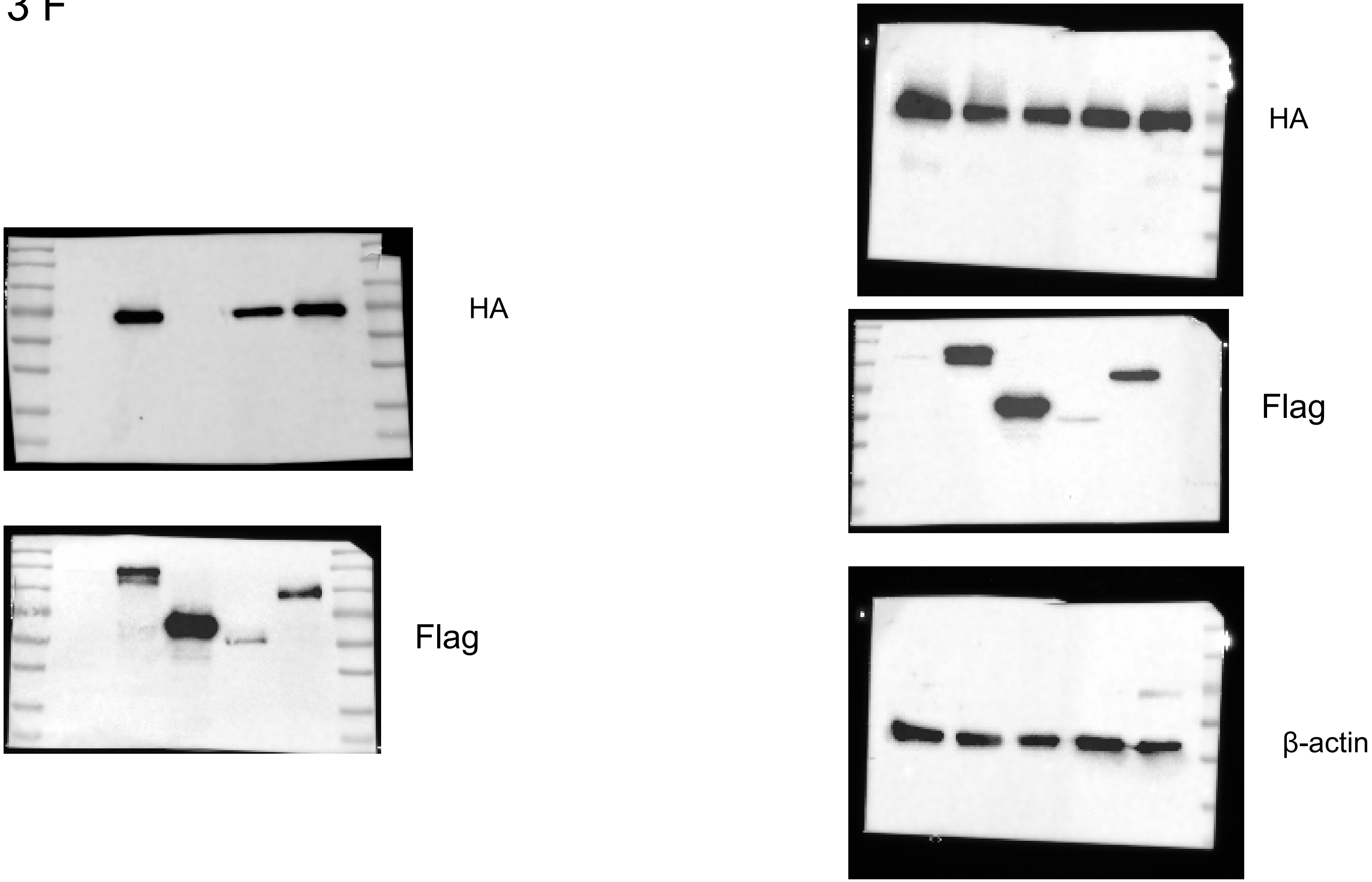

Figure 3 G

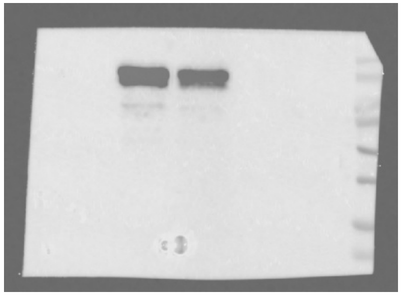

Flag

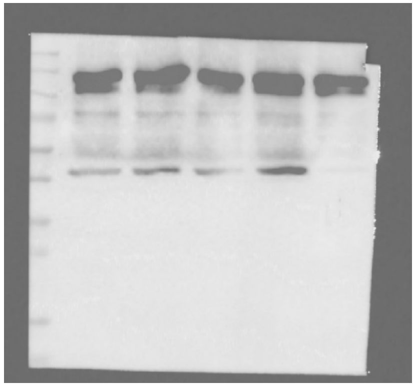

Flag

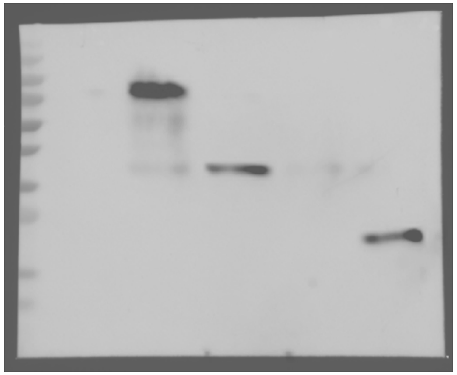

HA

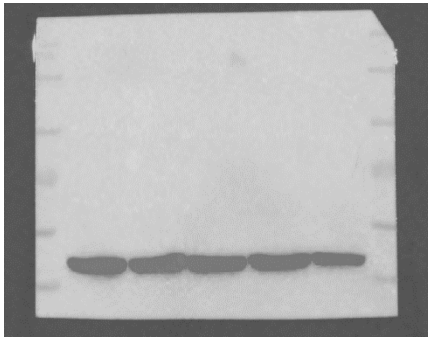

β-actin

Figure 3 H

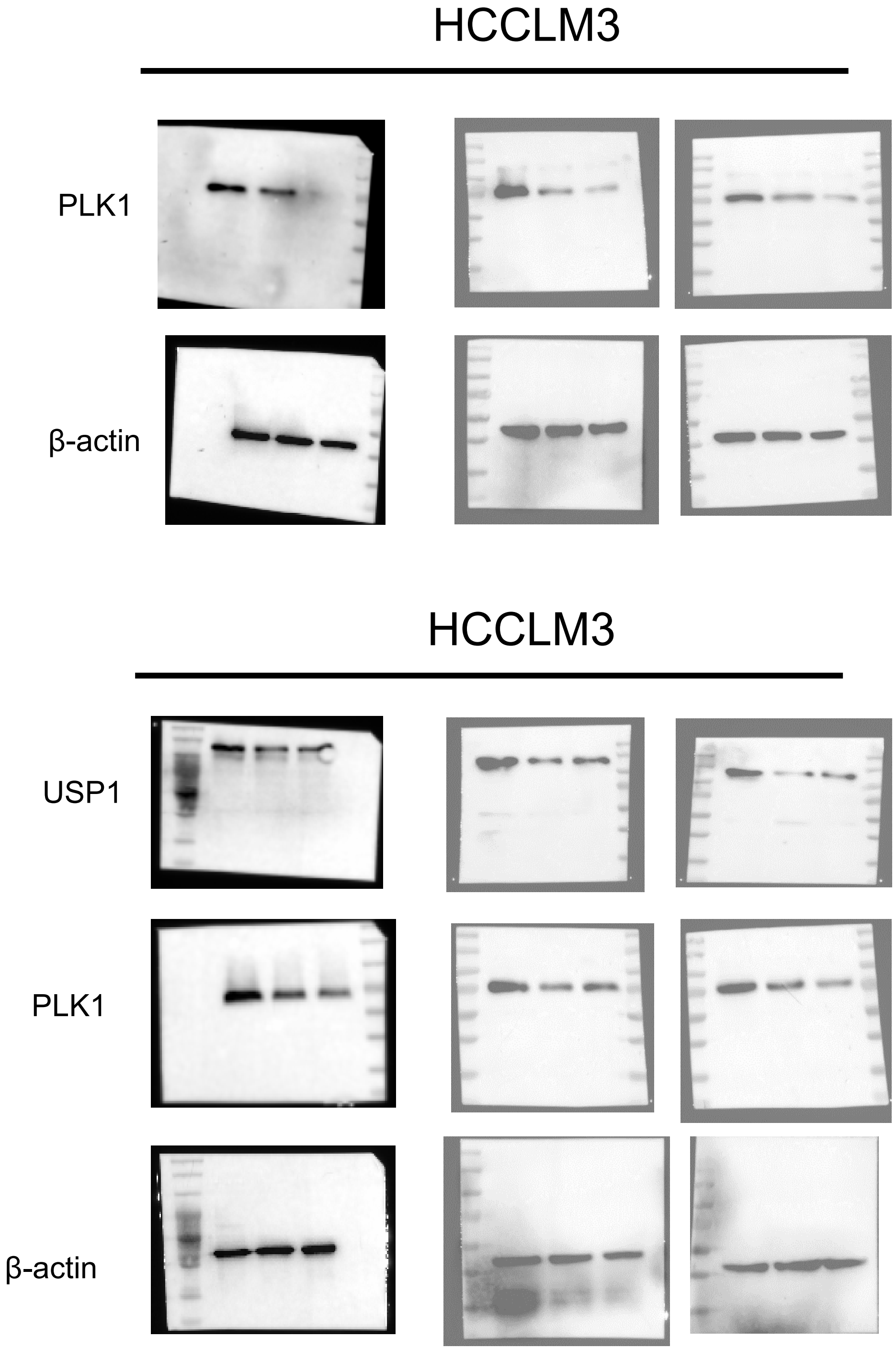

Figure 3 H

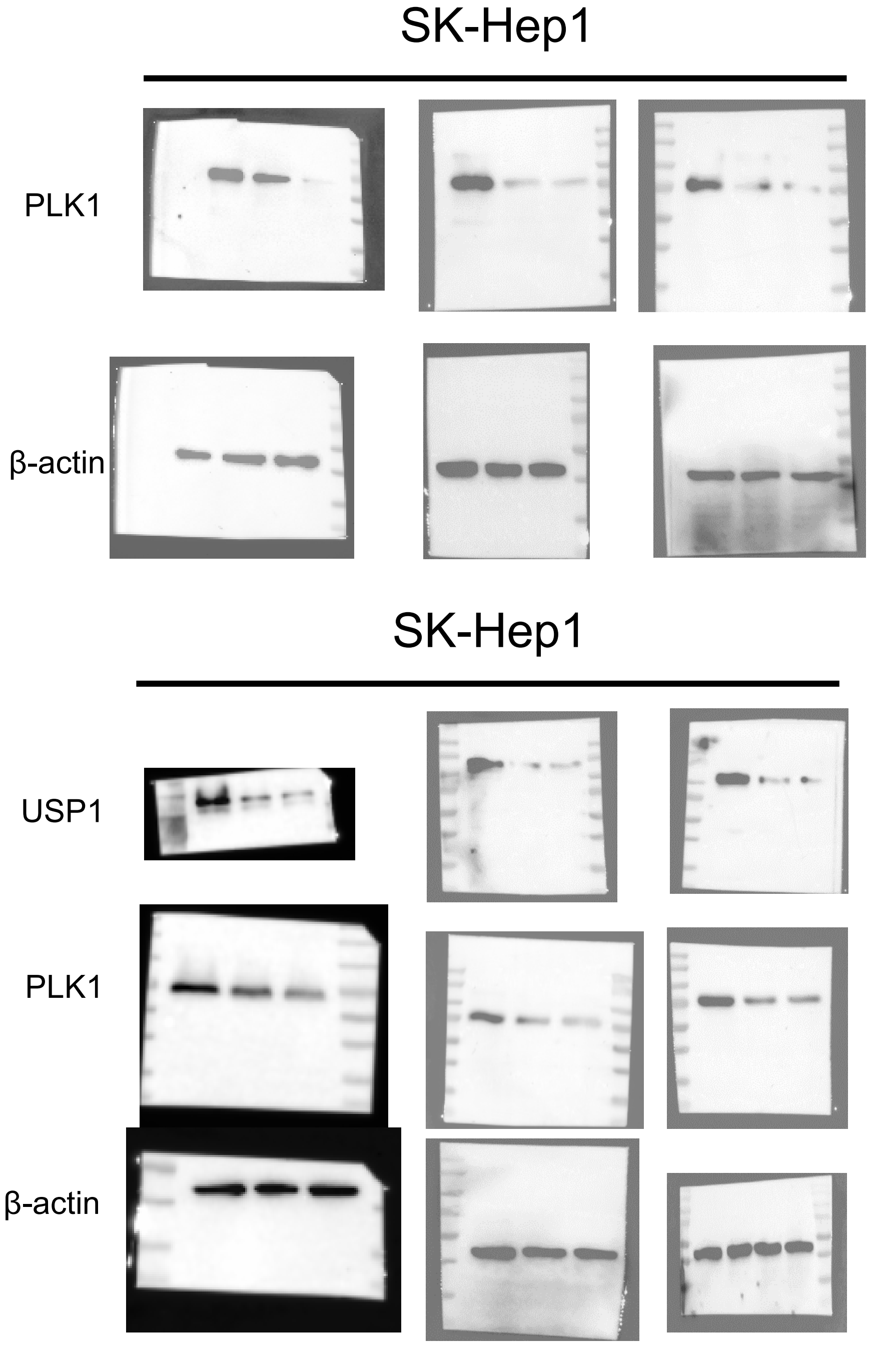

Figure 3 H

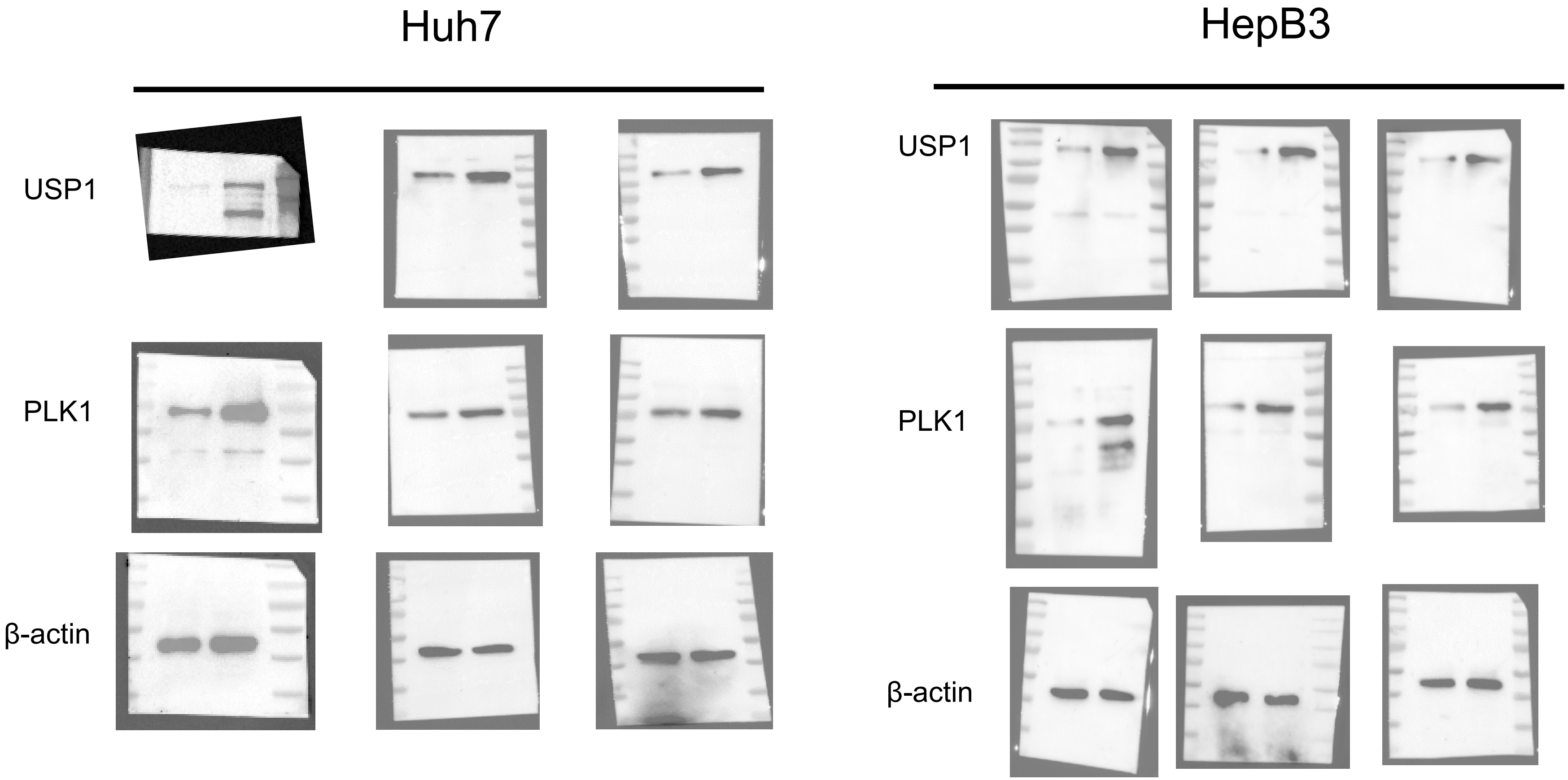

Figure 4A

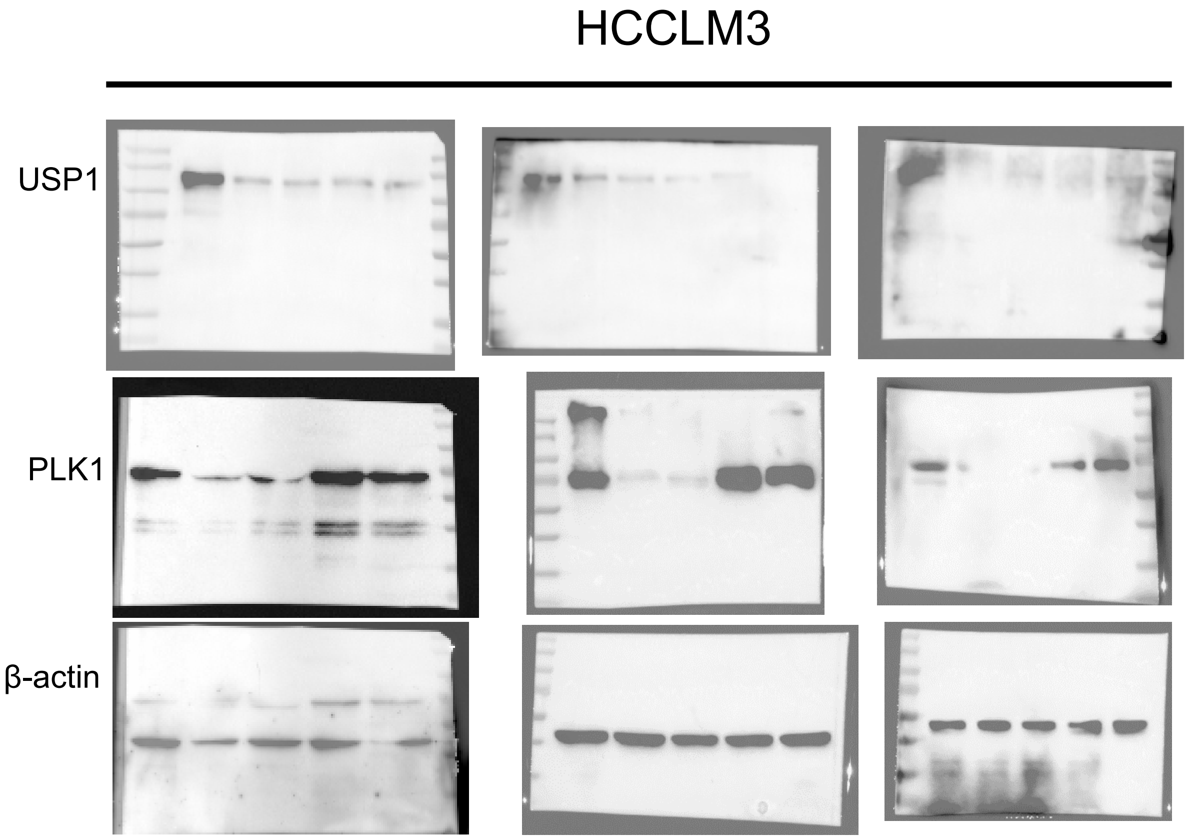

Figure 4A

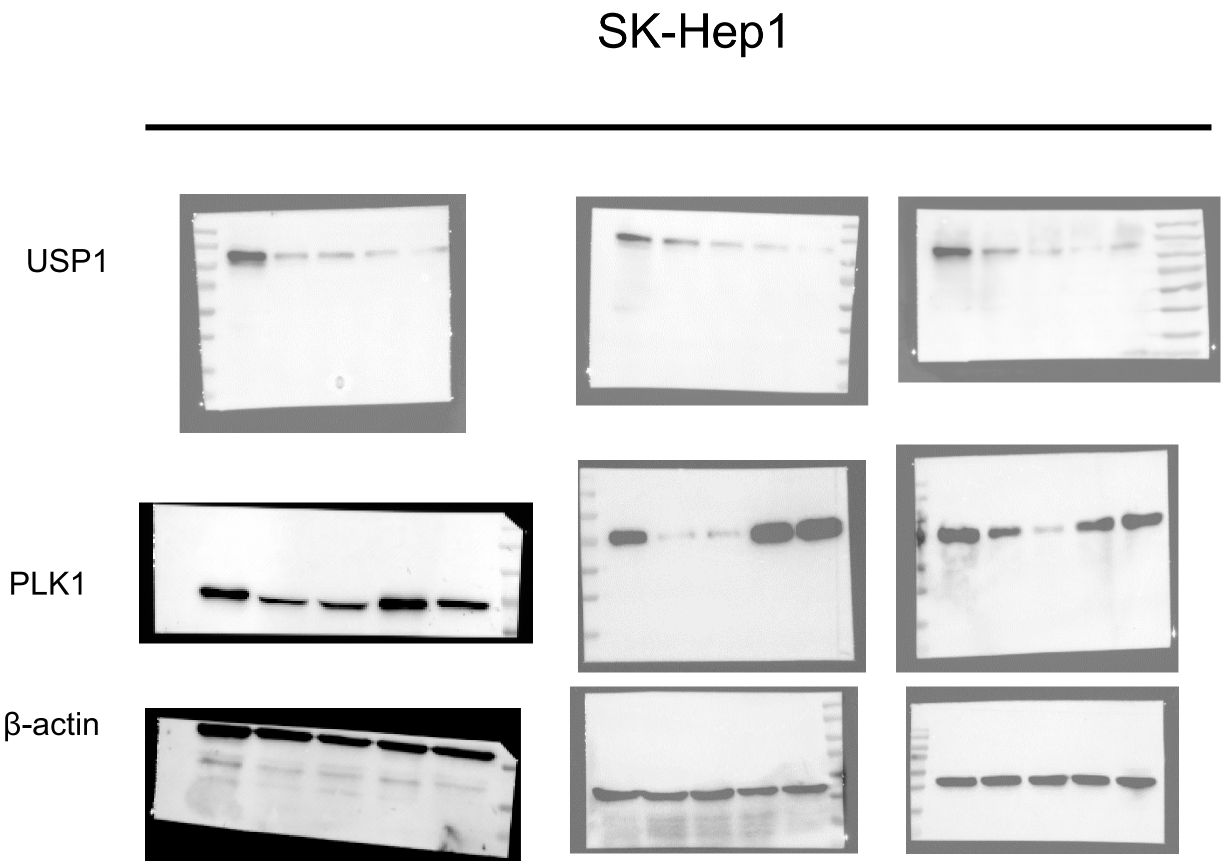

Figure 4B

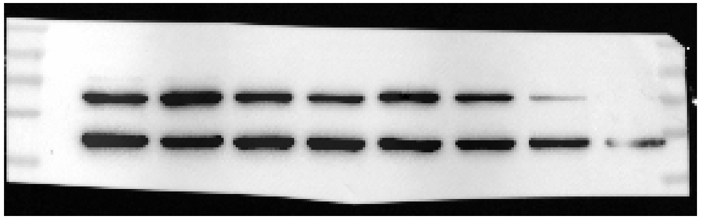

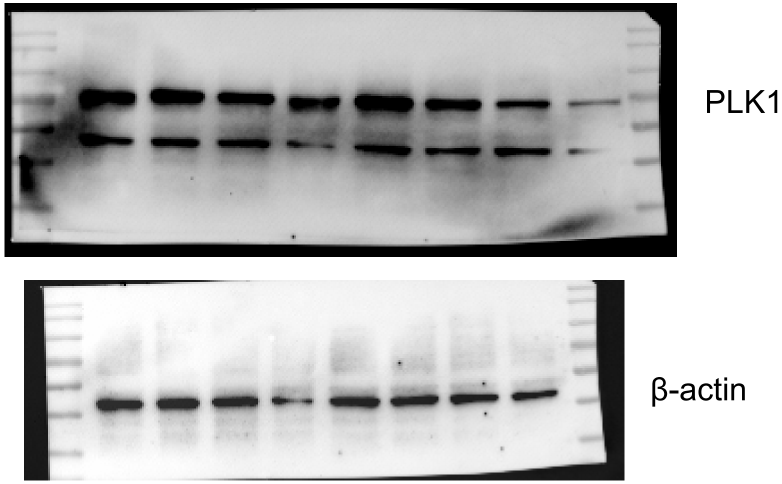

Figure 4C

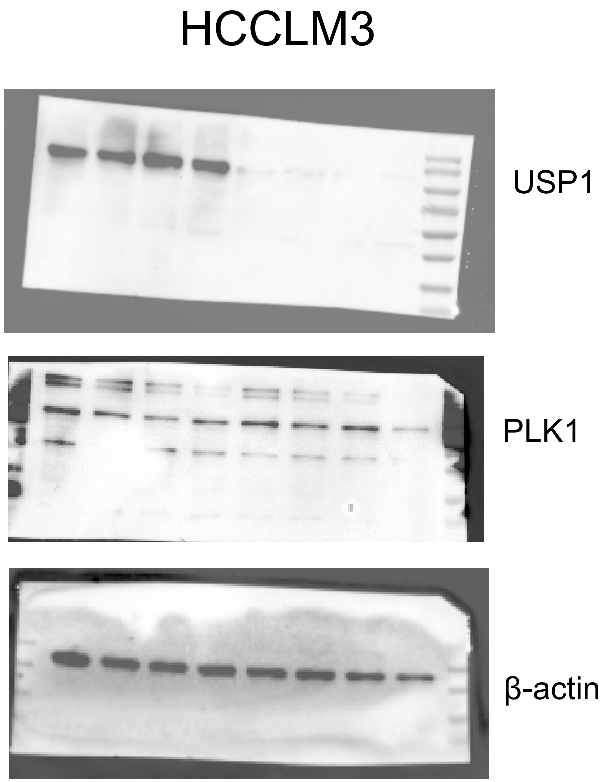

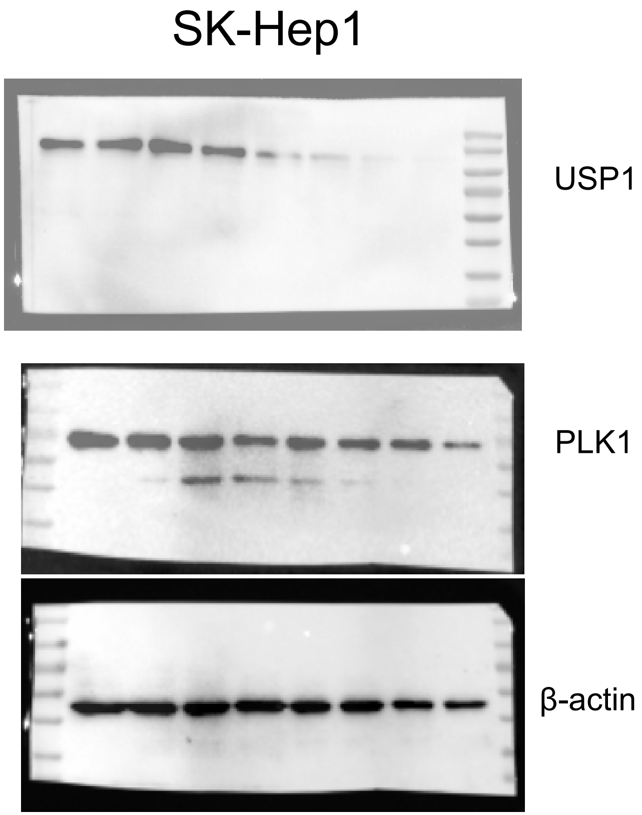

Figure 4C

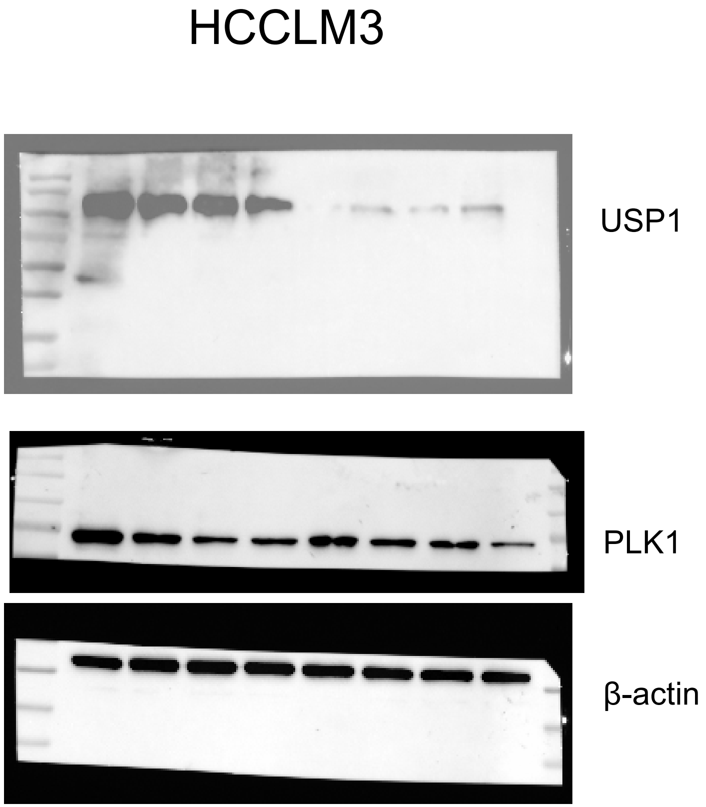

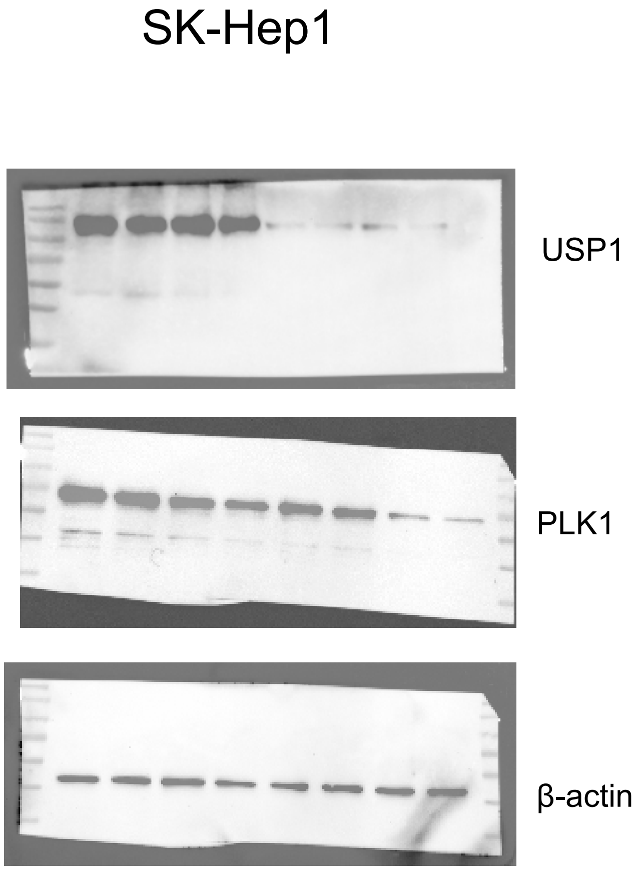

Figure 4D

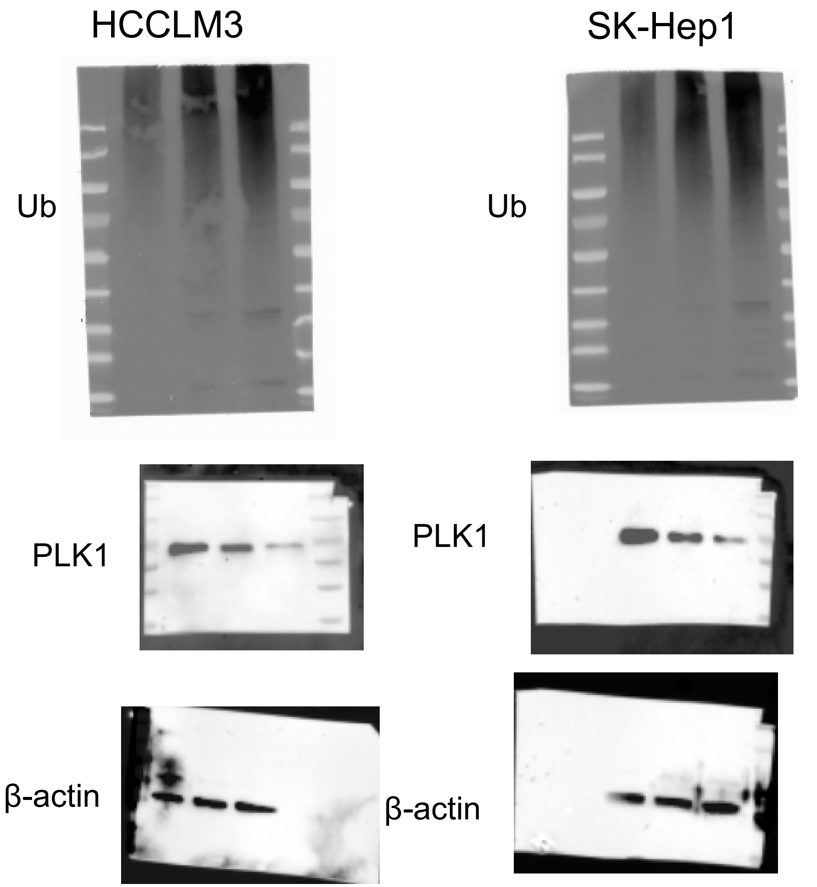

Figure 4E

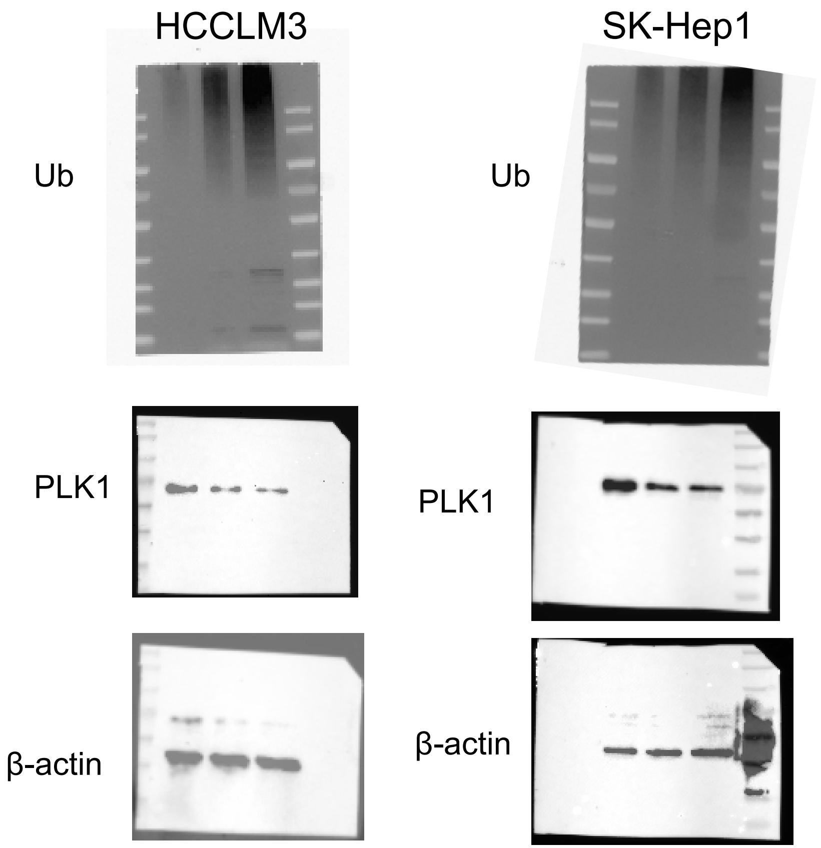

Figure 4F

Huh7

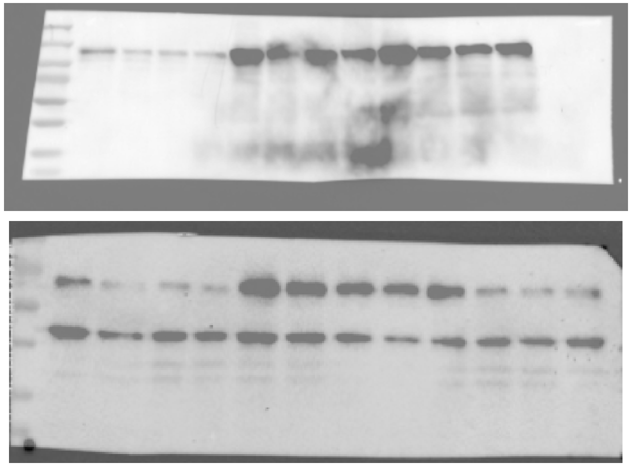

USP1

PLK1

β-actin

Hep3B

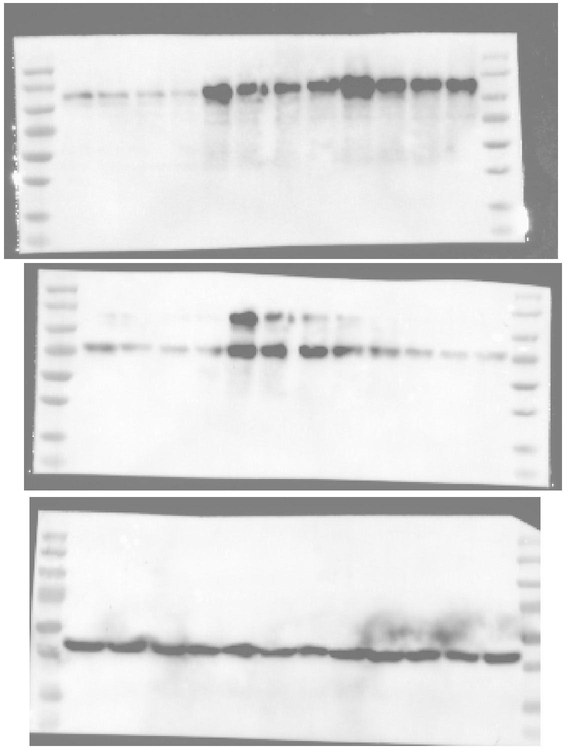

USP1

PLK1

β-actin

Figure 4G

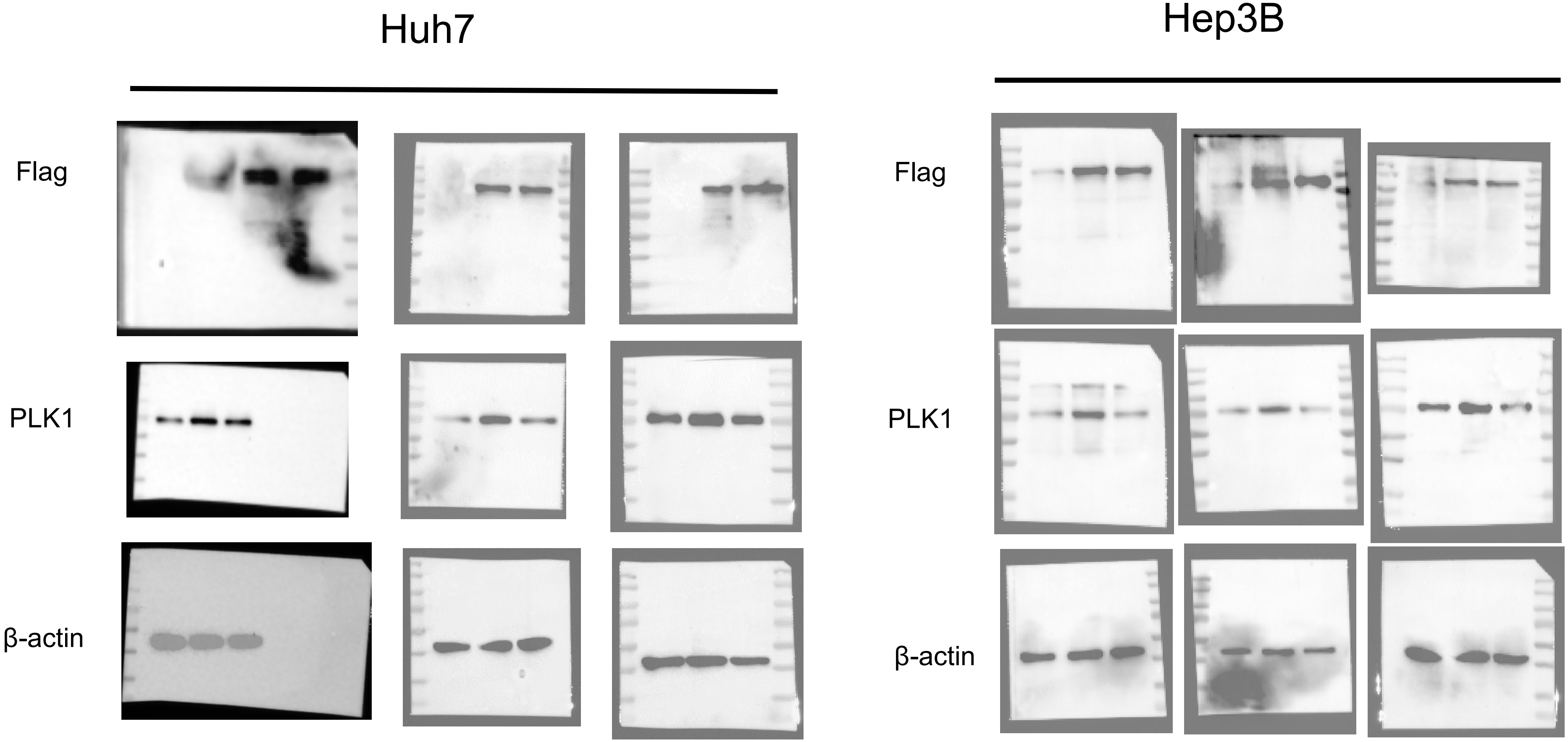

Figure 4H

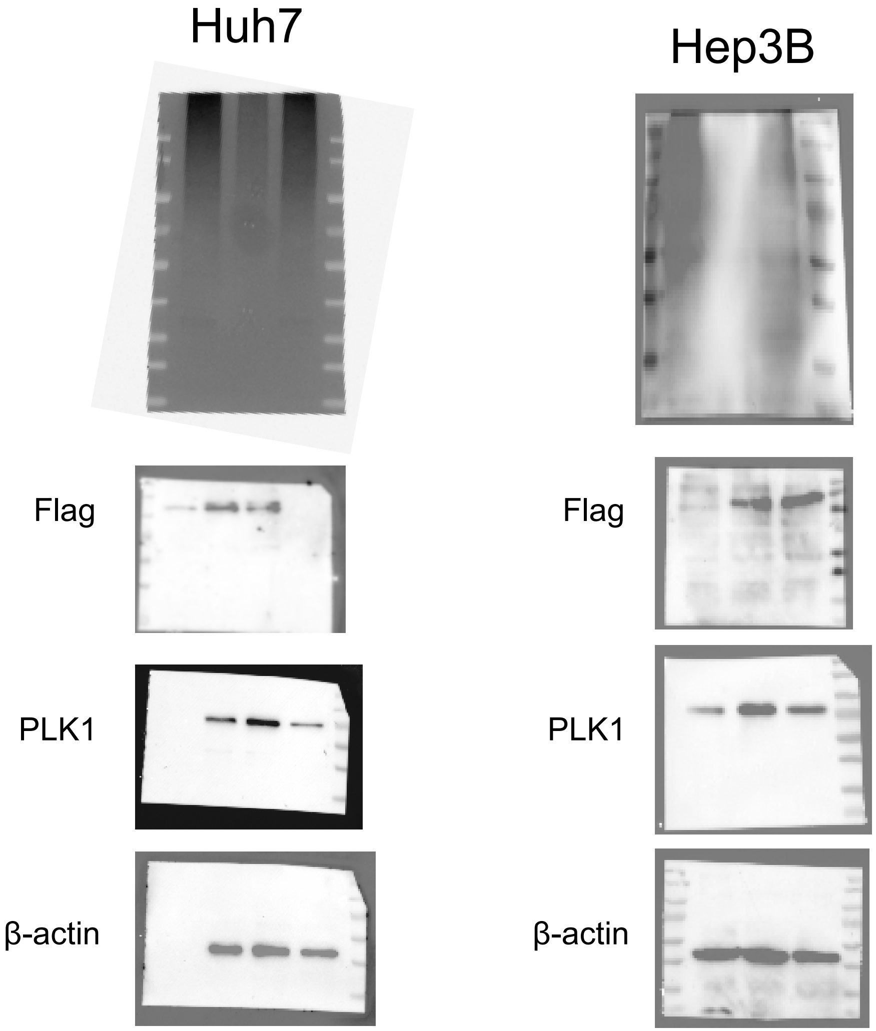

Figure 4 I

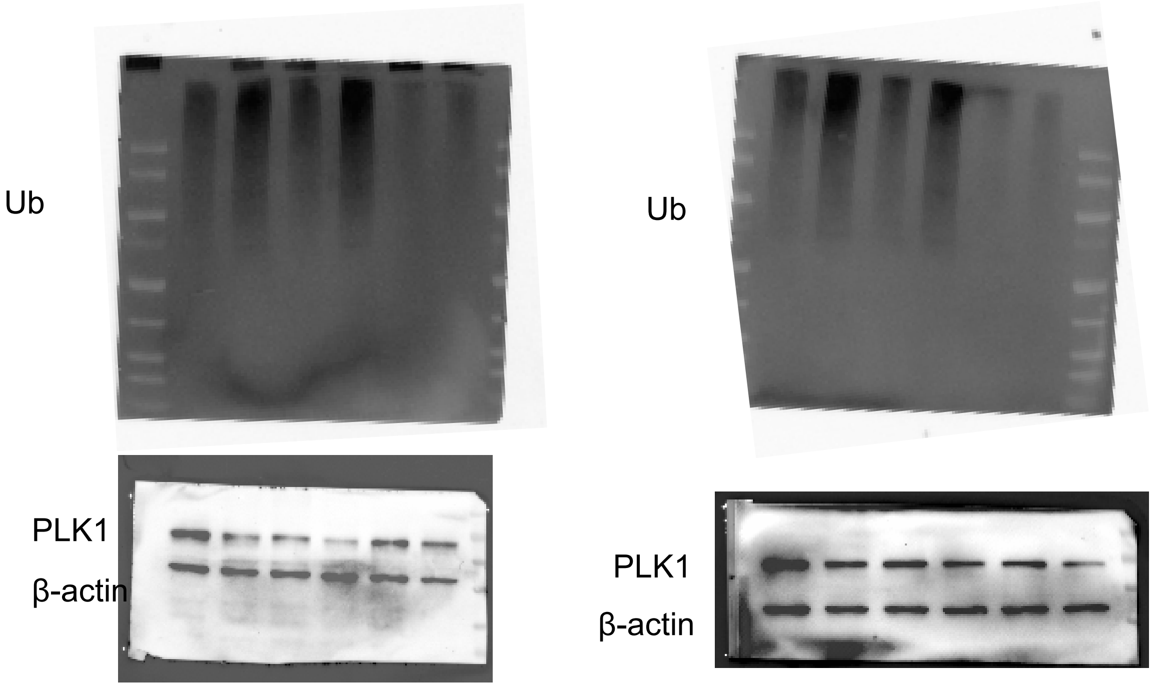

Figure 4 J

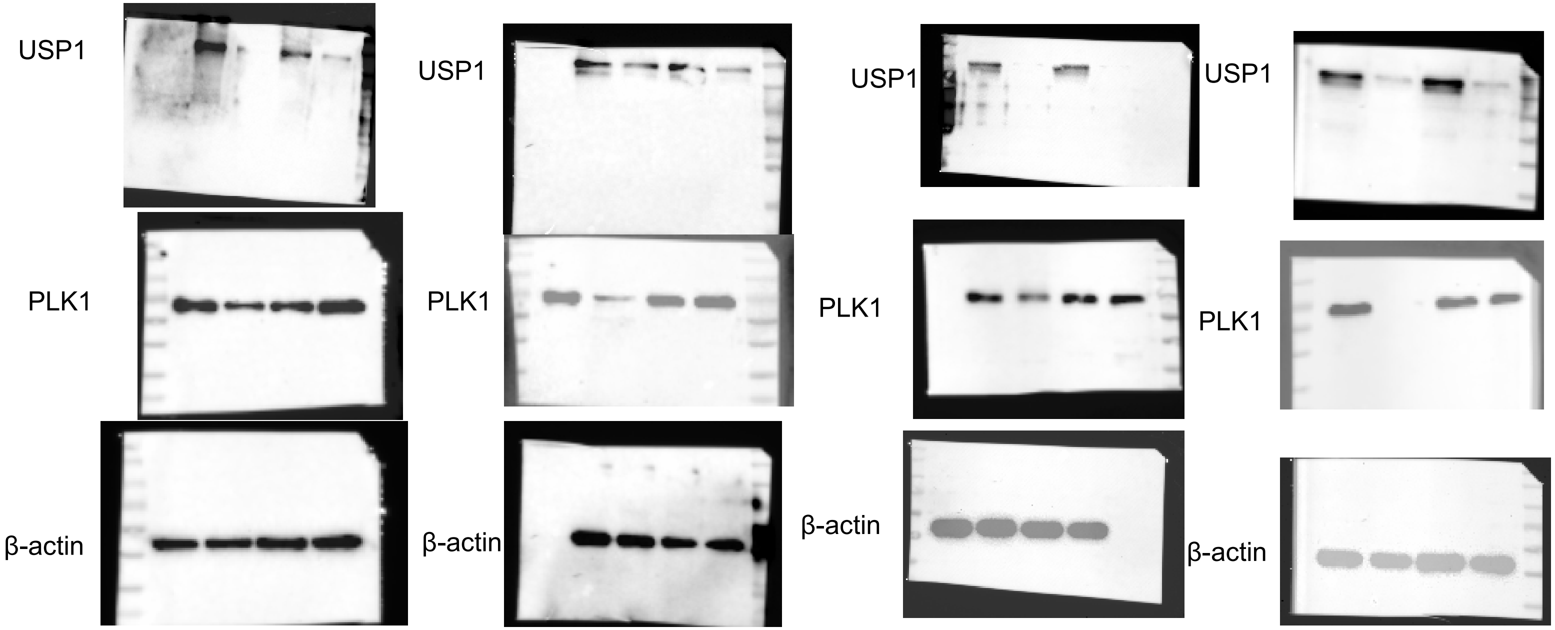
USP1
PLK1
β-actin
USP1
PLK1
β-actin
USP1
PLK1
β-actin
USP1
PLK1
β-actin

Figure 5 D

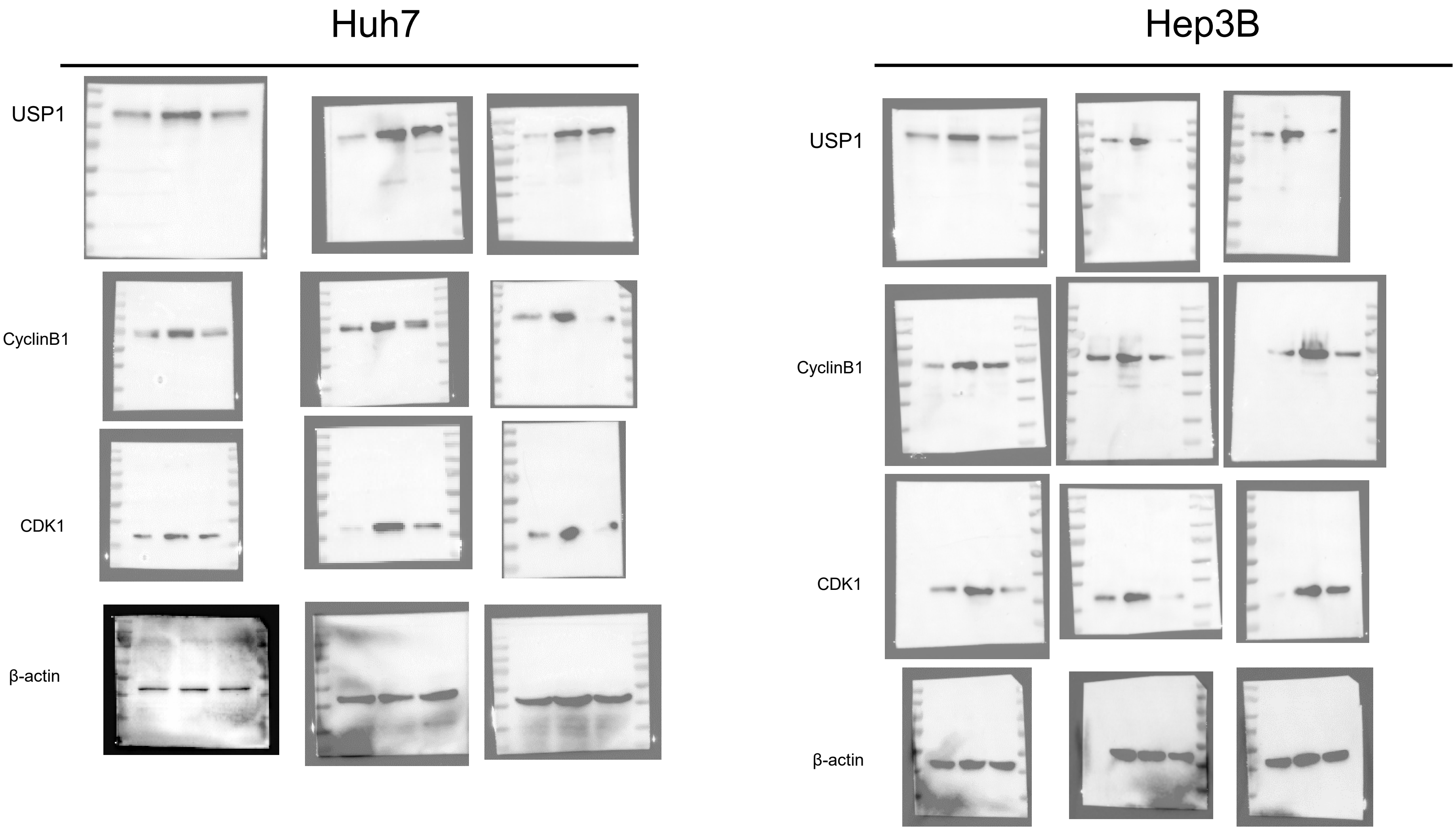
Huh7
Hep3B
USP1
CyclinB1
CDK1
β-actin
USP1
CyclinB1
CDK1
β-actin

Figure 5 I

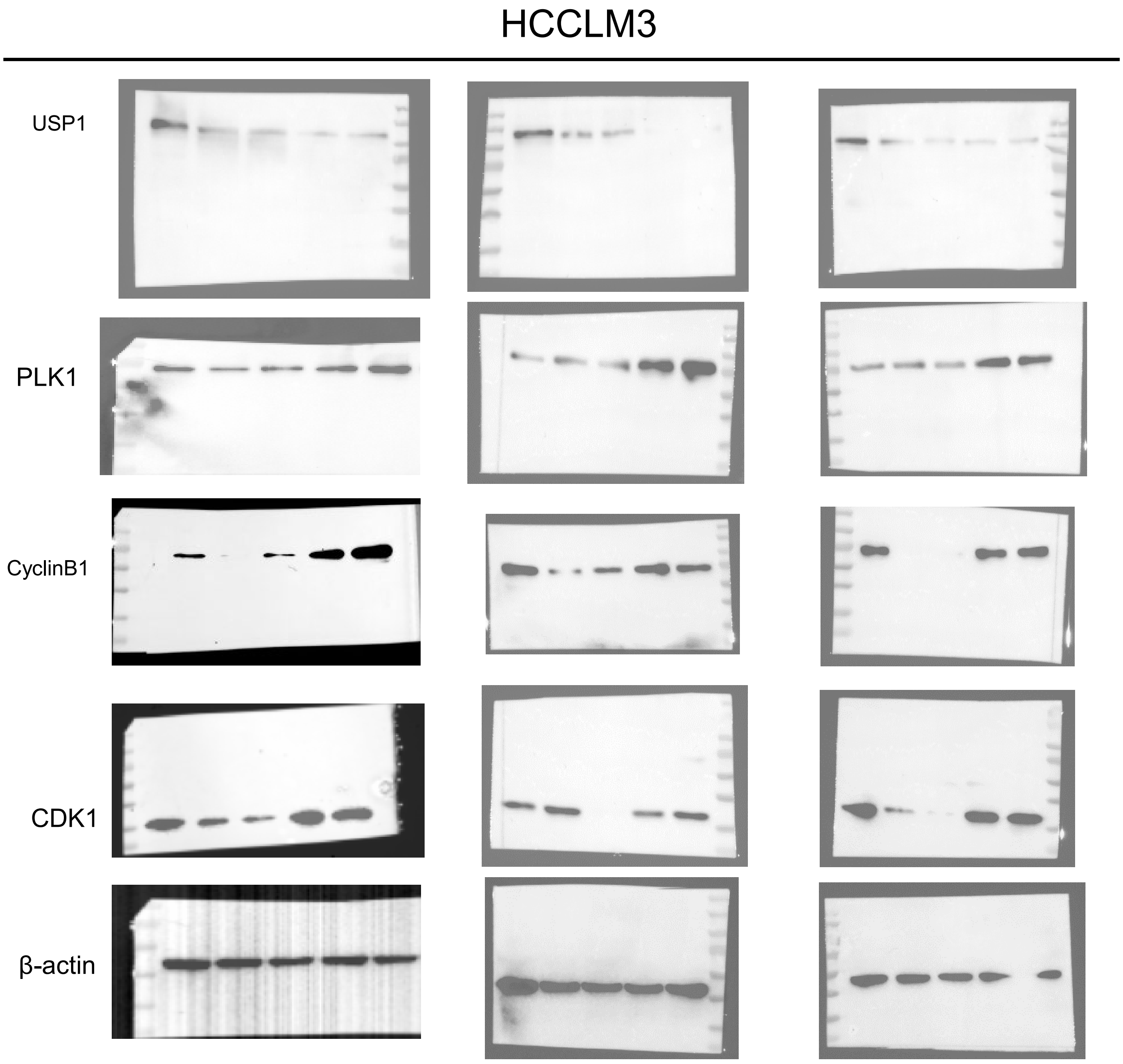

Figure 5 I

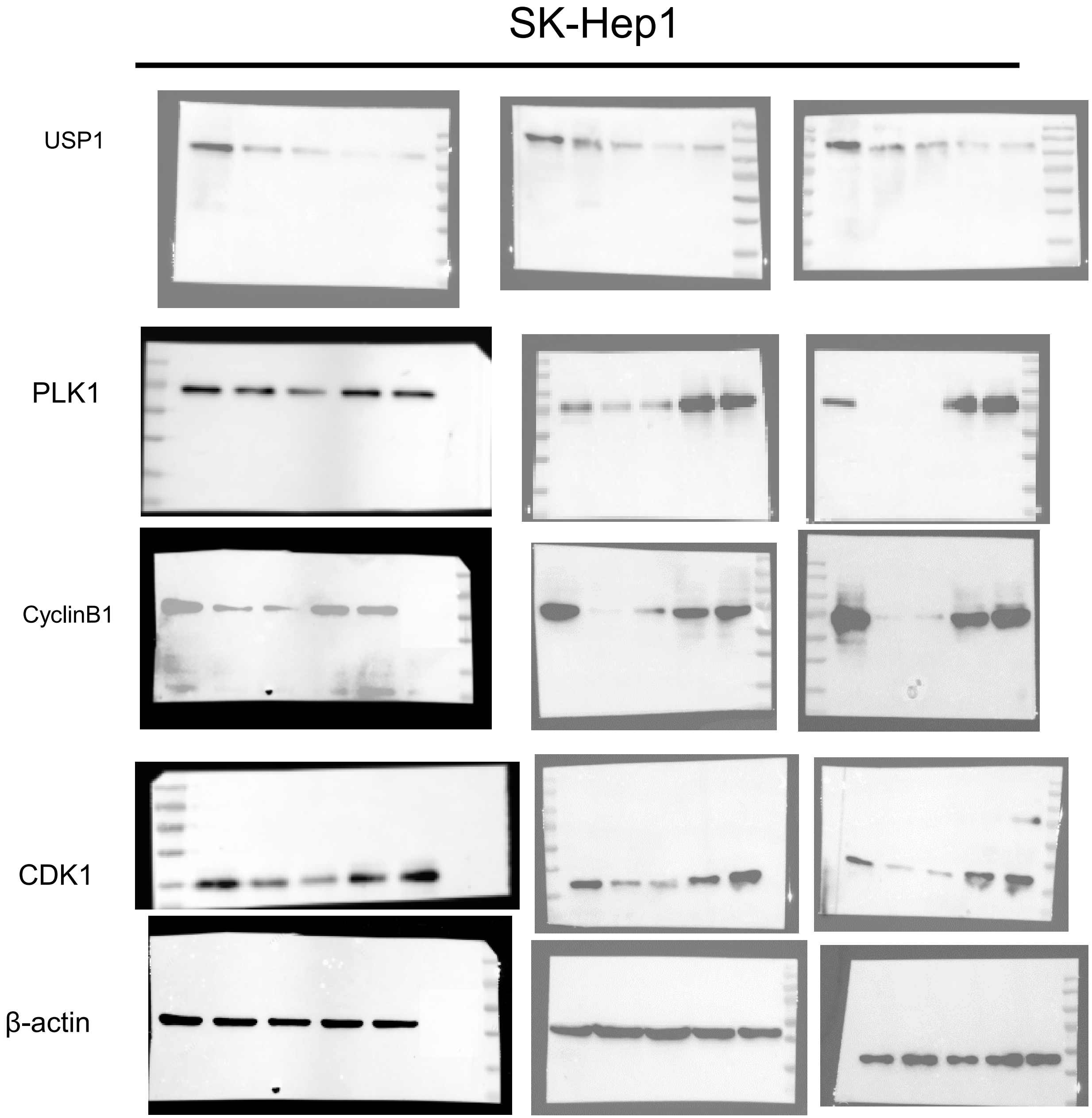

Figure 6 B

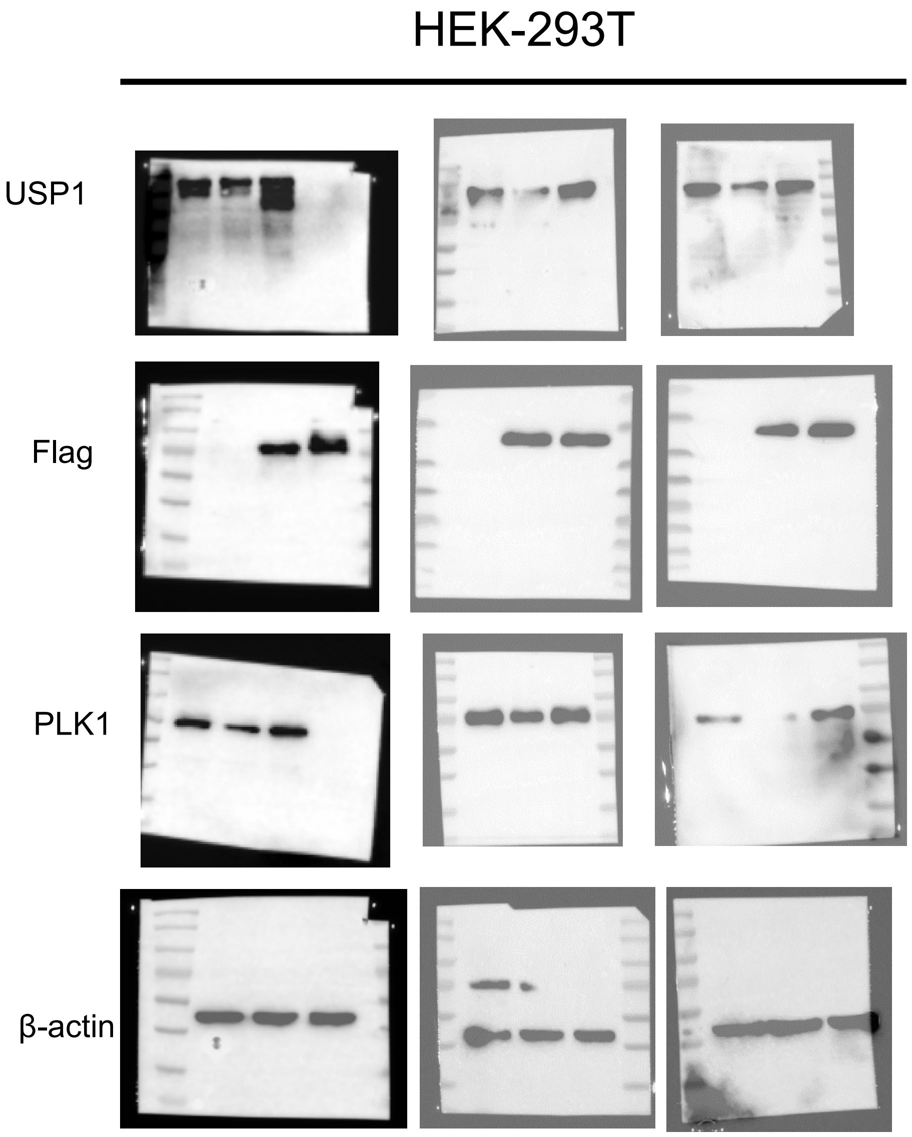

Figure 6 D

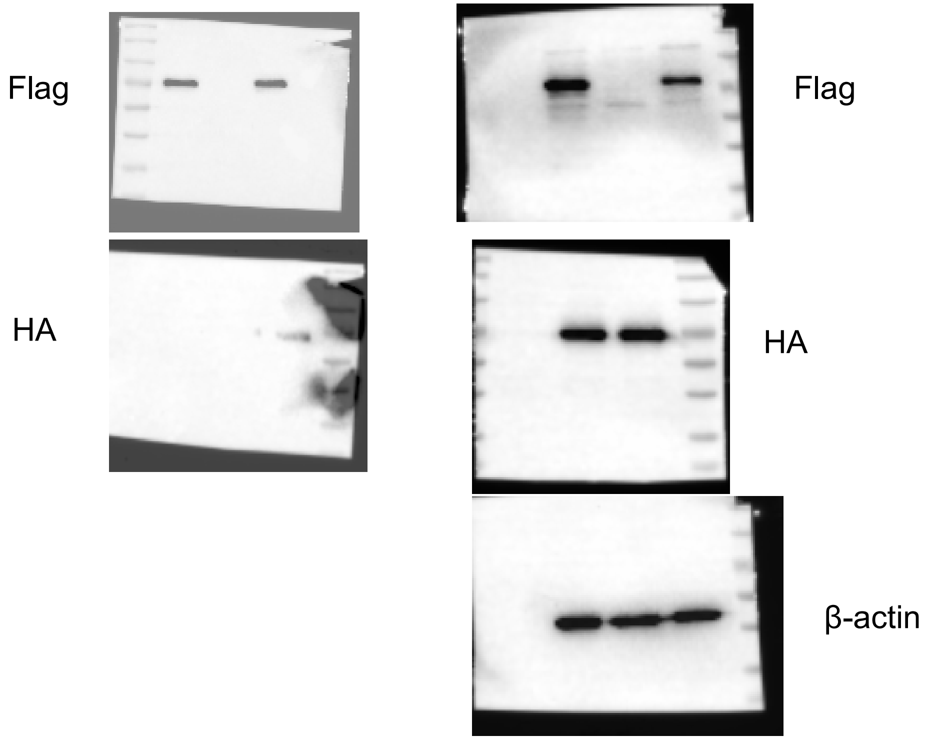

Figure 6 F

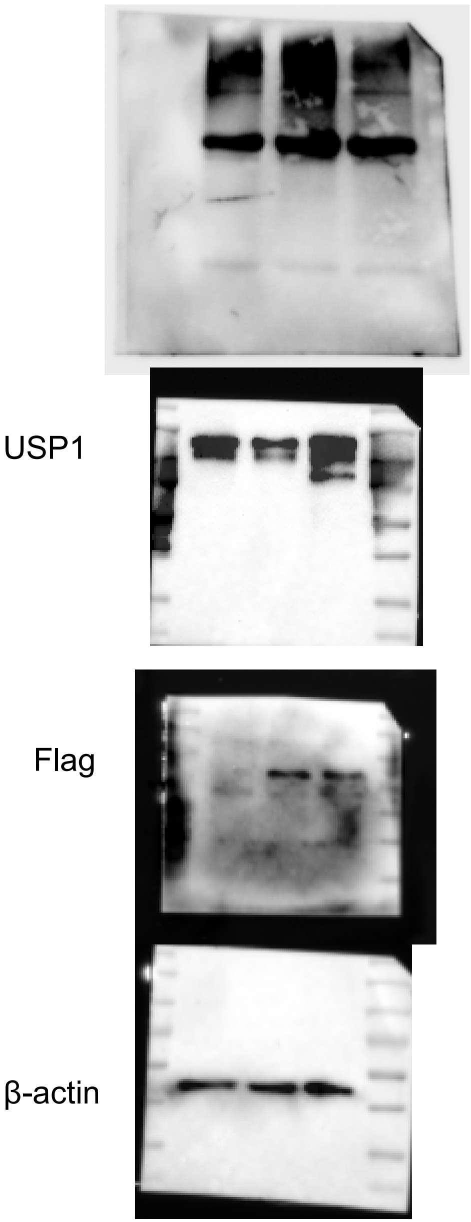

Figure 6G

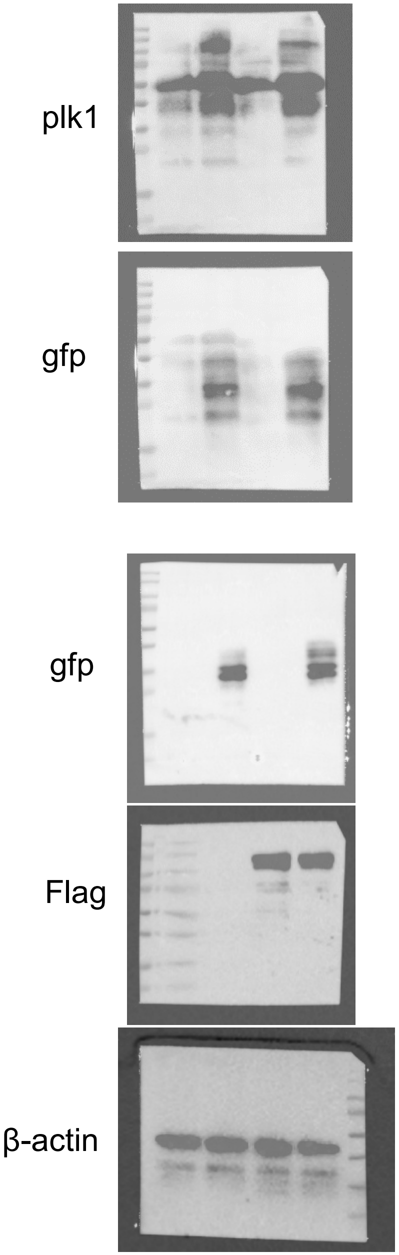

Figure 6H

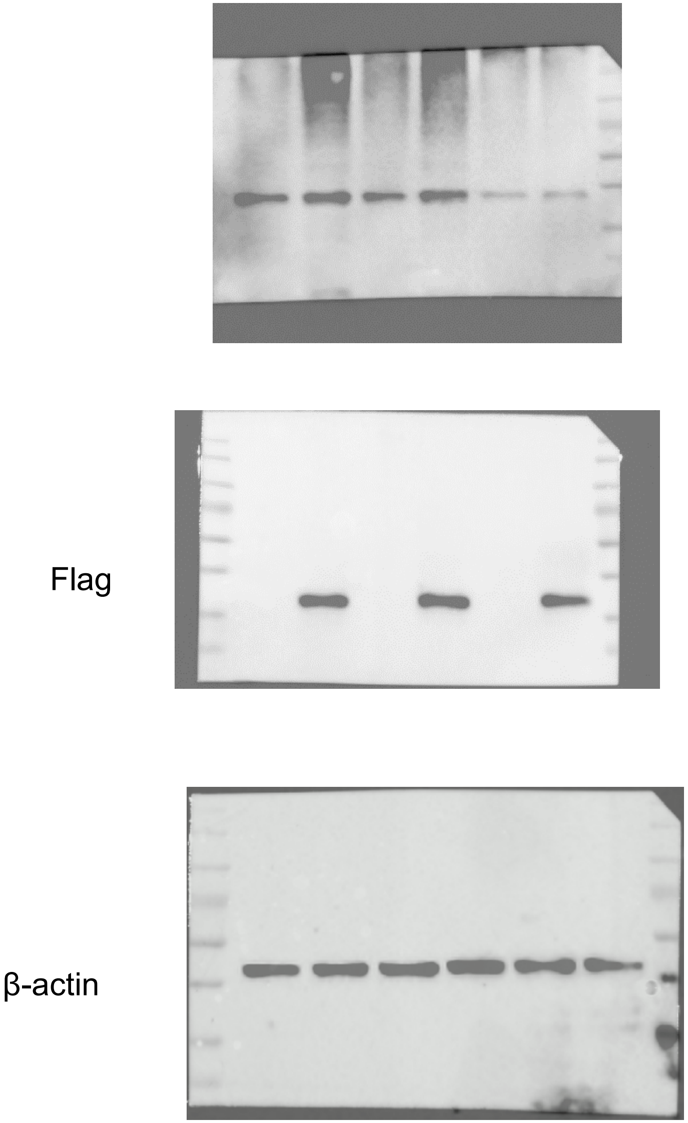

Figure 7 A

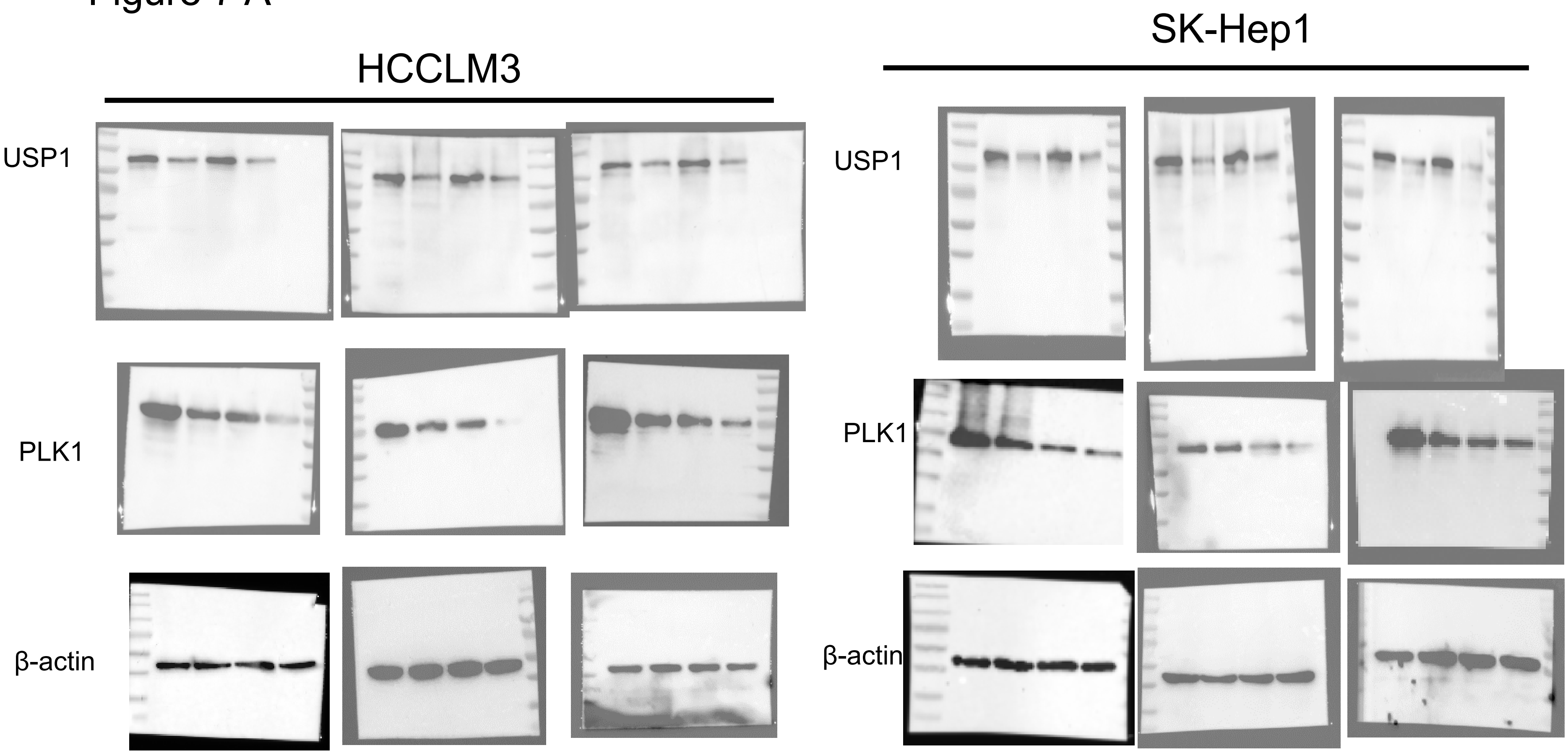

Figure 7 B

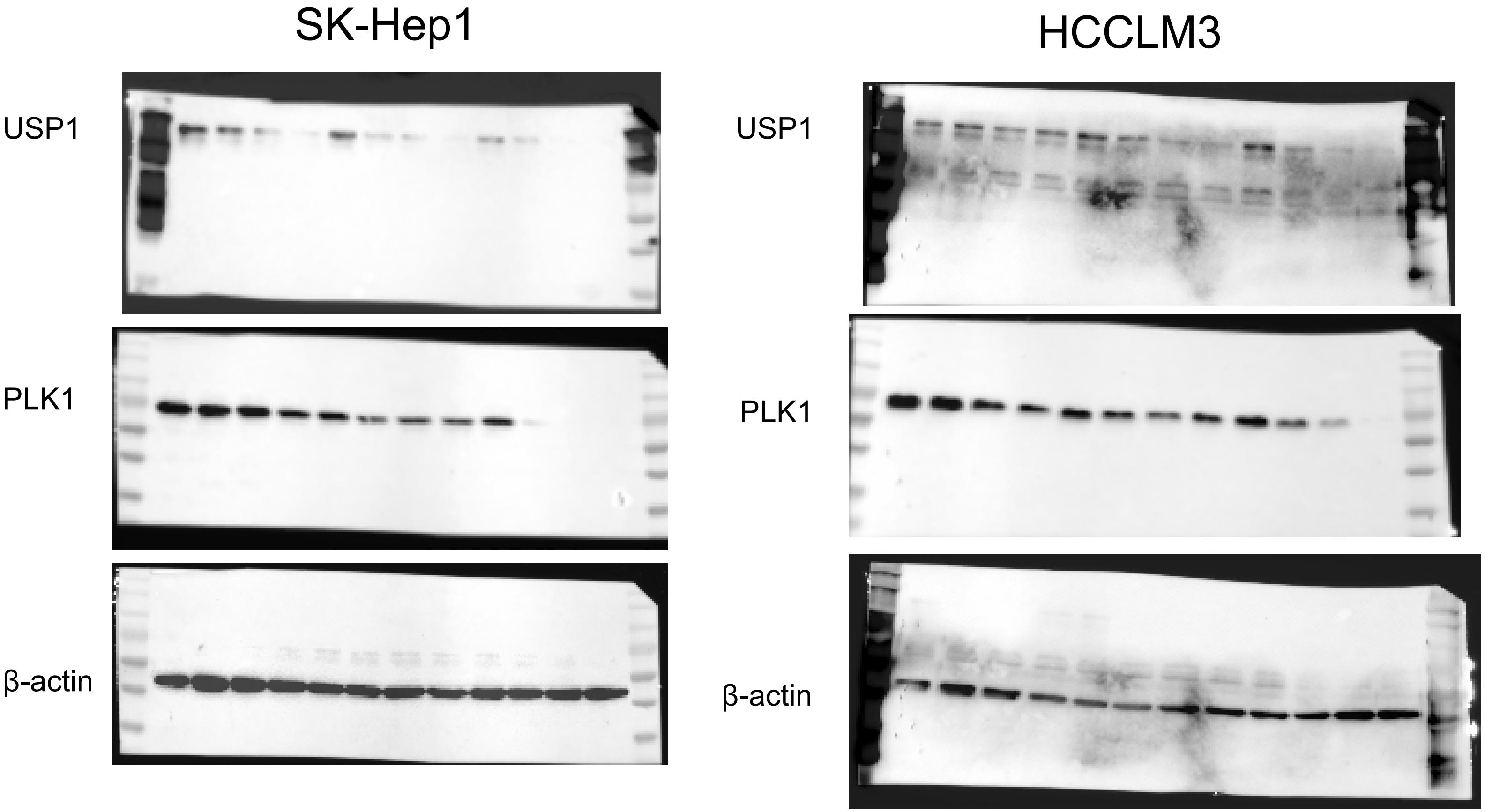

Figure 8D

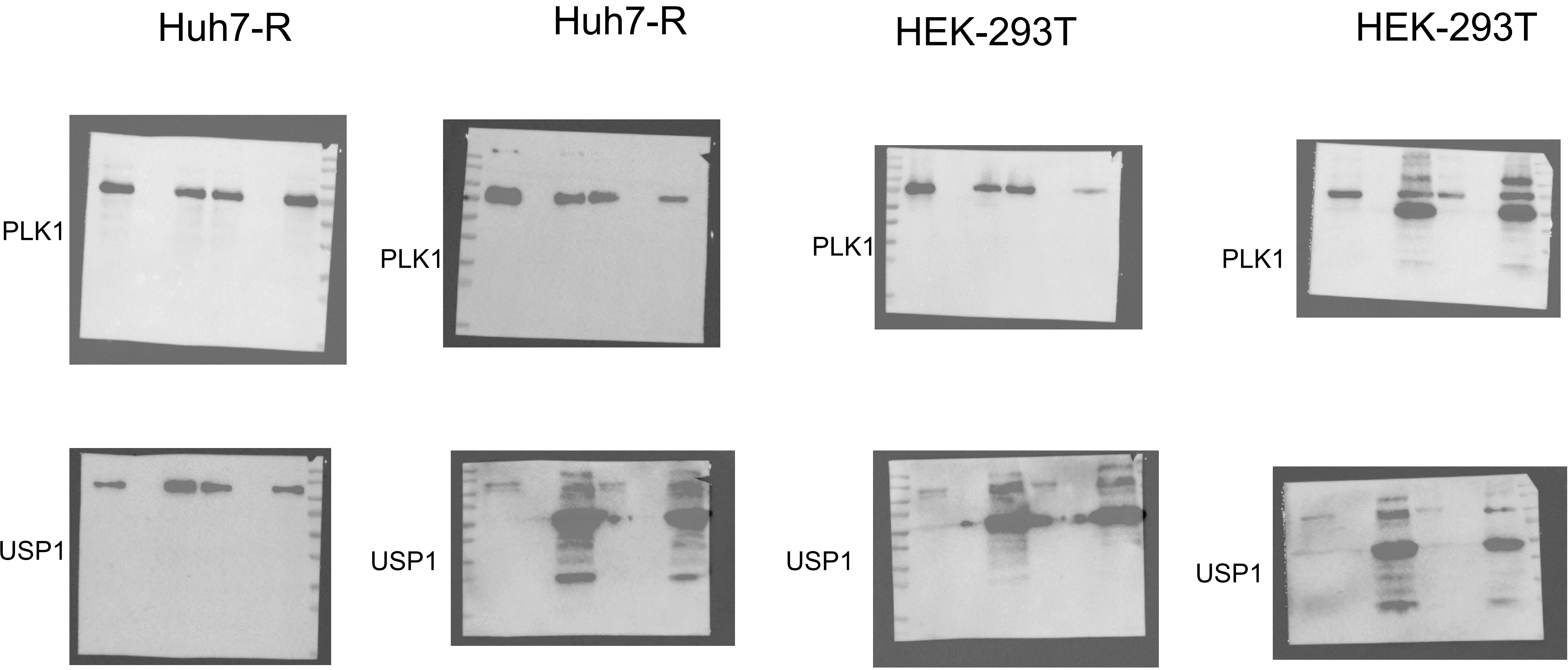
Huh7-R
Huh7-R
HEK-293T
HEK-293T
PLK1
PLK1
PLK1
PLK1
USP1
USP1
USP1
USP1

Figure 8E

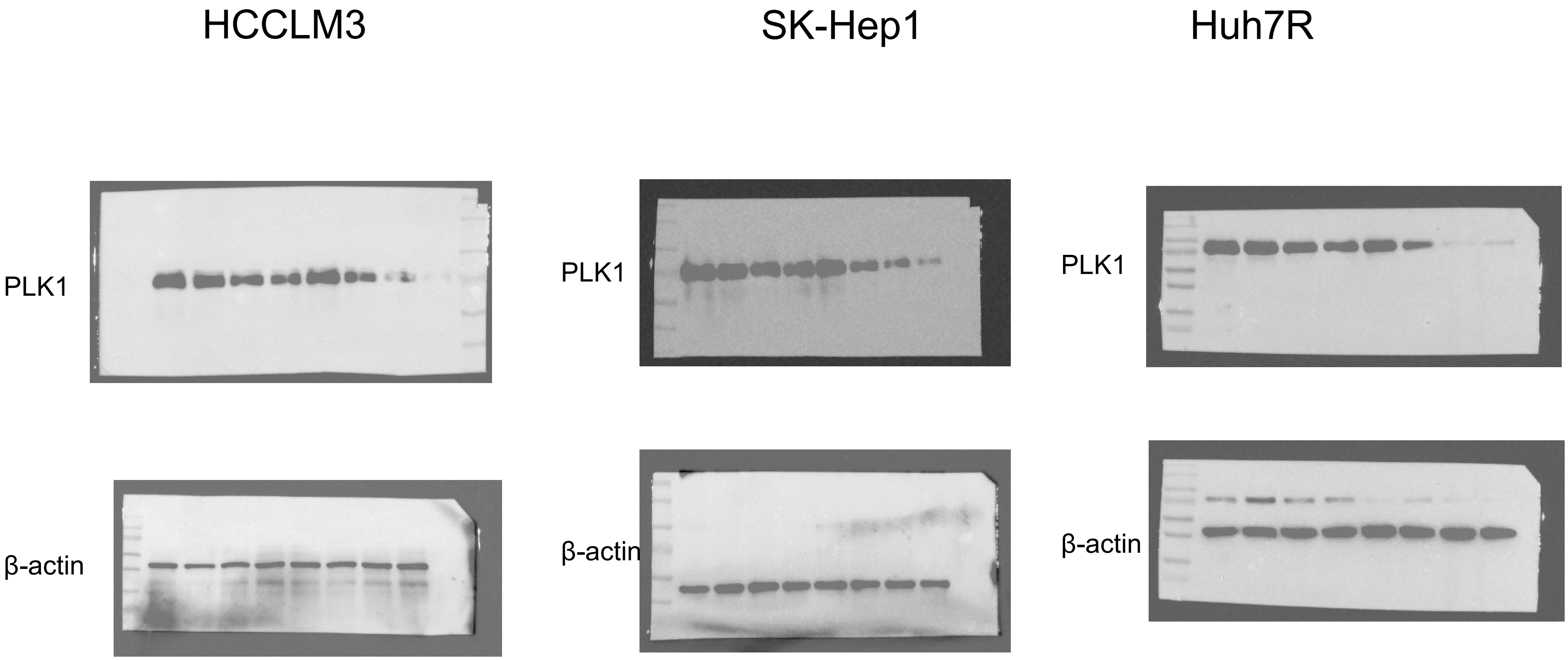

Figure 8F

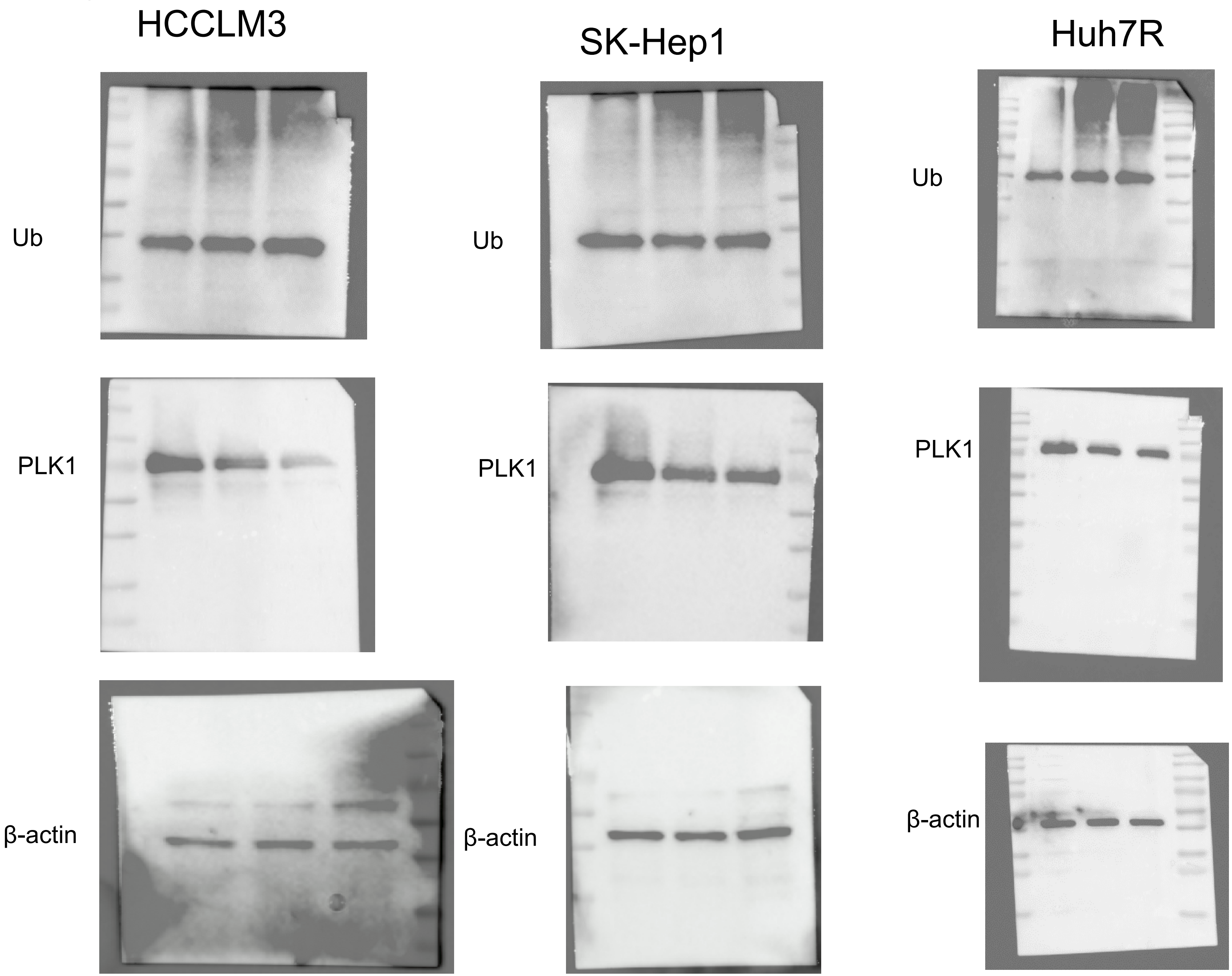
HCCLM3
SK-Hep1
Huh7R
Ub
Ub
Ub
PLK1
PLK1
PLK1
β-actin
β-actin
β-actin

Figure S6A

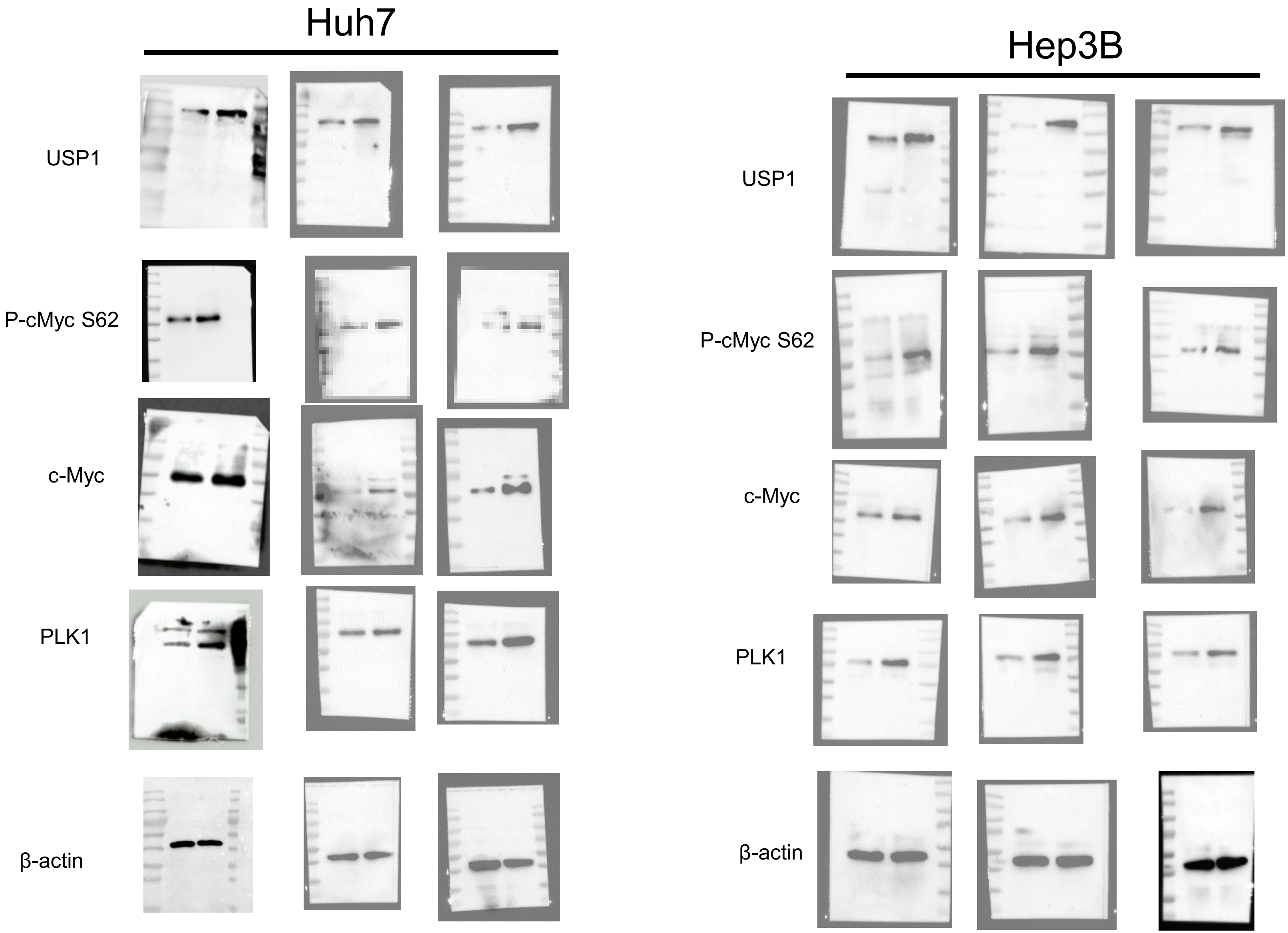

Figure S6A

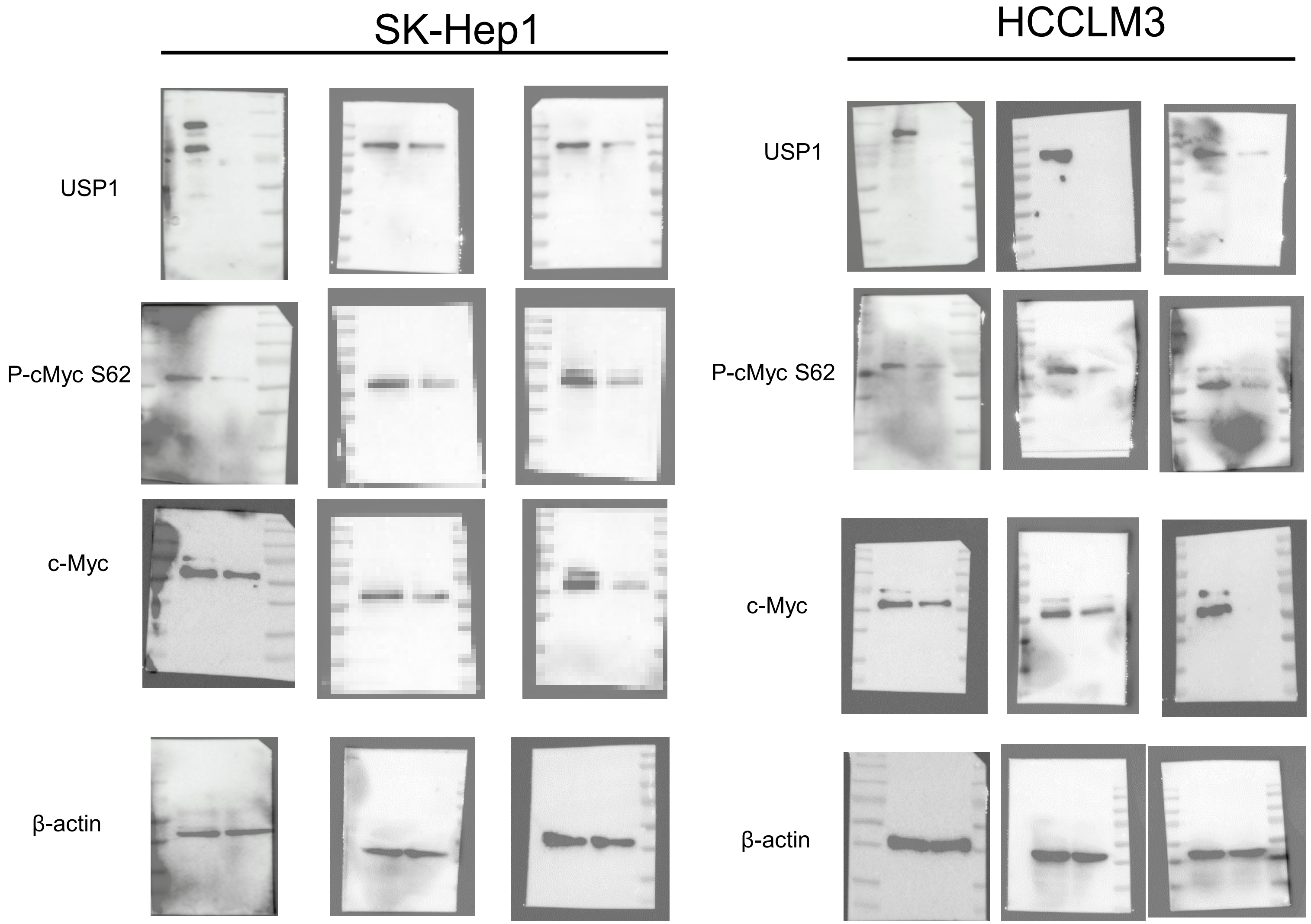

SK-Hep1
HCCLM3
USP1
P-cMyc S62
c-Myc
β-actin
USP1
P-cMyc S62
c-Myc
β-actin

Figure S6

E

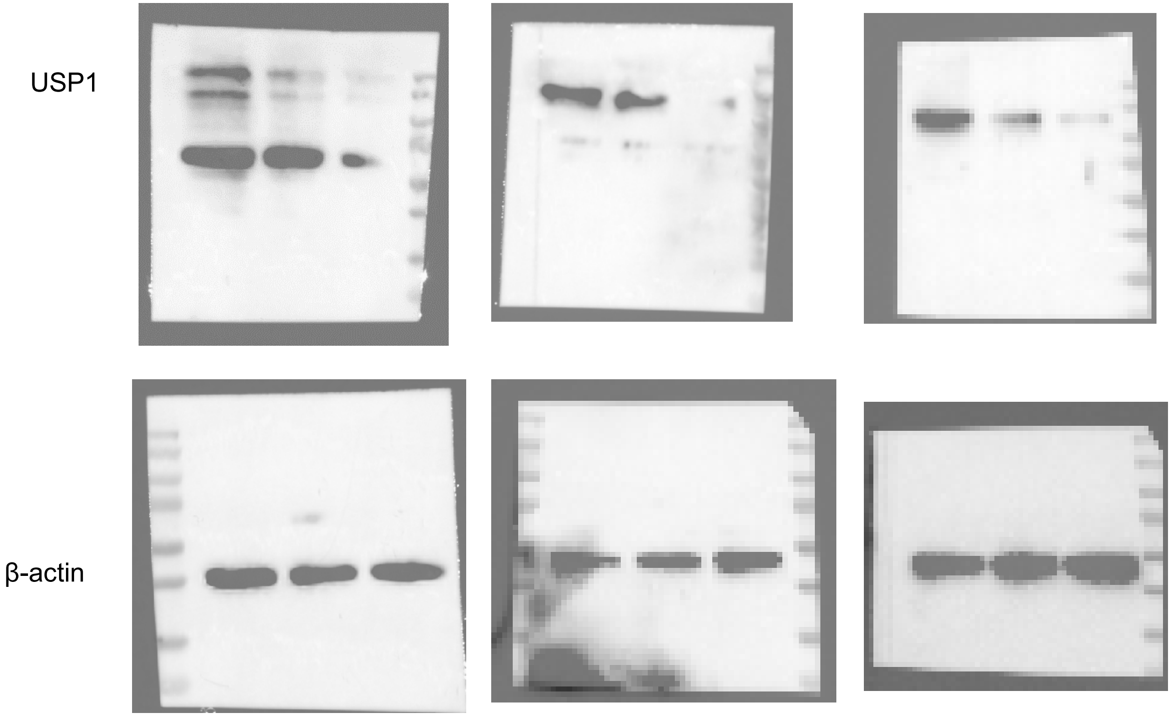

Supplement: Supplementary file 2 — Supplementary Material 2. [file 13046_2026_3683_MOESM2_ESM.pdf]
